# Depletion of activated macrophages with a folate receptor-beta-specific antibody improves symptoms in mouse models of rheumatoid arthritis

**DOI:** 10.1186/s13075-019-1912-0

**Published:** 2019-06-07

**Authors:** Yingwen Hu, Bingbing Wang, Jiayin Shen, Stewart A. Low, Karson S. Putt, Hans W. M. Niessen, Eric L. Matteson, Linda Murphy, Clemens Ruppert, Gerrit Jansen, Stephen J. Oliver, Yang Feng, Dimiter S. Dimitrov, Cheryl Nickerson-Nutter, Philip S. Low

**Affiliations:** 1 Department of Chemistry, Purdue University, 560 Oval Drive, West Lafayette, IN 47907 USA; 2 Institute for Drug Discovery, Purdue University, West Lafayette, IN 47907 USA; 3 Department of Pathology and Cardiac Surgery, ACS, Amsterdam UMC, location VUMC, Amsterdam, The Netherlands; 4 Division of Rheumatology, and Department of Health Sciences Research, Mayo Clinic, Rochester, MN USA; 5 Department of Biochemistry and Molecular Biology, Mayo Clinic College of Medicine, Rochester, MN USA; 6 Justus-Liebig University Giessen, Department of Internal Medicine, Biomedizinisches Forschungszentrum Seltersberg, Giessen, Germany; 7 Department of Rheumatology, Amsterdam Rheumatology and Immunology Center, VU University Medical Center, Amsterdam, The Netherlands; 8 Department of Medicine, New York University School of Medicine, New York, NY USA; 9 Protein Interactions Section, Laboratory of Experimental Immunology, Cancer and Inflammation Program, Center for Cancer, National Cancer Institute-Frederick, National Institutes of Health, Frederick, MD 21702 USA; 10 Center for Antibody Therapeutics, University of Pittsburgh, Pittsburgh, PA 15216 USA; 11 HuLow Medical, Northbrook, IL USA

**Keywords:** Folate receptor beta, Activated macrophages, Inflammatory disease, Autoimmune disease, Rheumatoid arthritis

## Abstract

**Objectives:**

Most therapies for autoimmune and inflammatory diseases either neutralize or suppress production of inflammatory cytokines produced by activated macrophages (e.g., TNFα, IL-1, IL-6, IL-17, GM-CSF). However, no approved therapies directly target this activated subset of macrophages.

**Methods:**

First, we undertook to examine whether the folate receptor beta (FR-β) positive subpopulation of macrophages, which marks the inflammatory subset in animal models of rheumatoid arthritis, might constitute the prominent population of macrophages in inflamed lesions in humans. Next, we utilized anti-FR-β monoclonal antibodies capable of mediating antibody-dependent cell cytotoxicity (ADCC) to treat animal models of rheumatoid arthritis and peritonitis.

**Results:**

Human tissue samples of rheumatoid arthritis, Crohn’s disease, ulcerative colitis, idiopathic pulmonary fibrosis, nonspecific interstitial pneumonia, chronic obstructive pulmonary disease, systemic lupus erythematosus, psoriasis, and scleroderma are all characterized by dramatic accumulation of macrophages that express FR-β, a protein not expressed on resting macrophages or any other healthy tissues. A monoclonal antibody to FR-β accumulates specifically in inflamed lesions of murine inflammatory disease models and successfully treats such models of rheumatoid arthritis and peritonitis. More importantly, elimination of FR-β-positive macrophages upon treatment with an anti-FR-β monoclonal antibody promotes the departure of other immune cells, including T cells, B cells, neutrophils, and dendritic cells from the inflamed lesions.

**Conclusions:**

These data suggest that specific elimination of FR-β-expressing macrophages may constitute a highly specific therapy for multiple autoimmune and inflammatory diseases and that a recently developed human anti-human FR-β monoclonal antibody (m909) might contribute to suppression of this subpopulation of macrophages.

**Electronic supplementary material:**

The online version of this article (10.1186/s13075-019-1912-0) contains supplementary material, which is available to authorized users.

## Background

Inflammatory and autoimmune diseases impose an enormous socio-economic burden on Western countries [1–3], with ~ 50–70 million people suffering an inflammatory disease and many patients finding little relief with current therapies [3–6]. The design of new medicines to treat these autoimmune diseases has focused largely on suppression of cytokine signaling with the expectation that inhibition of an essential cytokine’s function will disrupt a signaling network required for maintenance of the inflammatory state. Most current therapies achieve their therapeutic benefits by neutralizing TNFα (infliximab, etanercept, adalimumab, certolizumab, and golimumab), IL-1 (anakinra, rilonacept, and canakinumab), IL-6 (tocilizumab, siltuximab, and sarilumab), IL-12 (ustekinumab), IL-17 (secukinumab, brodalumab, ixekizumab), or granulocyte-macrophage colony-stimulating factor (mavrilimumab). Because activated monocytes/macrophages constitute the main source of these cytokines [7], the concept that eliminating/suppressing these activated myeloid cells might allow control of disease symptoms has often been entertained [8–13]. Indeed, animal studies suggest that nonspecific suppression of phagocytes with clodronate liposomes can mitigate symptoms of autoimmune and inflammatory diseases [9–11], albeit with toxic side effects. Unfortunately, no strategy for safely suppressing the activated subset of macrophages has ever been described in the literature.

To explore the goal of suppressing only activated macrophages in autoimmune diseases, a strategy was required that would deliver a therapeutic agent solely to pro- and anti-inflammatory macrophages without altering the larger population of quiescent or resting macrophages. In pursuit of this objective, we explored the possibility of directing our therapy solely towards folate receptor β (FR-β) expressing cells, since FR-β constitutes an isoform of FR that has only been found in myeloid cells (i.e., macrophages, monocytes, and some neutrophils [14, 15]) and is further thought to be limited to the activated pro- and anti-inflammatory subsets of these cells [14–31]. Based on this selectivity, FR-targeted imaging agents have been exploited to image autoimmune and inflammatory diseases in mice [16–20], rats [21], and humans [22, 23].

To exploit this selectivity, we have developed both a human anti-human (m909) and mouse anti-mouse (F3) FR-β specific monoclonal antibody (mAb) that exhibit no cross-reactivity towards other isoforms of the folate receptor. In the paper below, we provide the first evidence that FR-β-positive macrophages are abundant in virtually all autoimmune diseases, including rheumatoid arthritis, Crohn’s disease, ulcerative colitis, idiopathic pulmonary fibrosis, nonspecific interstitial pneumonia, chronic obstructive pulmonary disease, systemic lupus erythematosus, psoriasis, and scleroderma. We then show that antibody-dependent cell cytotoxicity (ADCC)-mediated elimination of FR-β-positive macrophages from inflammatory lesions suppresses the recruitment of other immune cells in murine models of both peritonitis and collagen-induced arthritis, leading ultimately to resolution of disease symptoms in both pathologies. Because depletion of this subset of macrophages occurs with no overt toxicity, we conclude that the FR-β positive subpopulation of macrophages constitutes an excellent target for treatment of autoimmune and inflammatory diseases and that use of the human anti-human FR-β monoclonal antibody could constitute a novel therapy for these diseases.

## Materials and methods

### Materials

Horseradish peroxidase (HRP)-streptavidin and EZ-Link Sulfo-NHS-LC-Biotin were purchased from Thermo Scientific (Madison, WI). Ethanol, xylene, hydrogen peroxide, and Tween-20 were obtained from Sigma-Aldrich (St. Louis, MO). Lung tissue samples from normal donors (31 samples), and patients with idiopathic pulmonary fibrosis (53 samples), nonspecific interstitial pneumonia (32 samples), or chronic obstructive pulmonary disease (40 samples) were kindly provided by Dr. Clemens Rupert (Justus-Liebig University Giessen). Tissue samples from patients with Crohn’s disease (12 samples), ulcerative colitis (9 samples), psoriasis (8 samples), scleroderma (4 samples), rheumatoid arthritis (30 samples), and systemic lupus erythematosus (6 samples) were generously supplied by Mayo Clinic (Rochester, MN). Use of tissue samples from Mayo Clinic was approved by the Mayo Clinic Institutional Review Board, which waived the need for consent of these stored samples (obtained in the context of clinical care). Use of all tissue samples was also approved by the Purdue University Institutional Review Board.

Anti-human IgG PE conjugate, anti-mouse IgG FITC, PE/Cy7 anti-CD11b (M1/70), APC anti-CD49b (DX5), Alexa Fluor® 488 anti-Ly-6G/Ly-6C (RB6-8C5), FITC anti-CD3 (17A2), PE anti-CD19 (1D3/CD19), Alexa Fluor® 647 anti-CD11c (N418), PE goat anti-mouse IgG (poly4053), recombinant human IL-4, recombinant human IFN-γ, recombinant human IL-10, and LEGENDplex™ Mouse Inflammation kit were purchased from Biolegend (San Diego, CA). Mice and mouse diet were purchased from Harlan Labs (Indianapolis, IN). CytoTox-ONE™ Homogeneous Membrane Integrity Assay kit and murine ADCC Reporter Bioassay were obtained from Promega (Madison, WI). Human M-CSF and human GM-CSF (Miltenyi Biotec; San Diego, CA), thioglycollate medium (Fisher Scientific; Chicago, IL), Cy3- and Cy-5 labeled F (ab’)_2_ fragments of goat anti-mouse antibody (Jackson Immunologic Laboratories; West Grove, PA), Hoechst Blue (Molecular Probes; Eugene, OR), anti-CD68 (Dako; Carpinteria, CA), Freund’s complete adjuvant (MP BioMedical; Santa Ana, CA), and PromoCell Monocyte Attachment Medium, RPMI, and FBS (VWR; Chicago, IL) were purchased from the indicated suppliers. A human anti-human FR-β mAb (m909) was generated against residues 23~236 of human FR-β as described previously [32]. For IHC studies, m909 was labeled with EZ-Link Sulfo-NHS-LC-Biotin according to manufacturer’s instructions (Thermo Scientific; Madison, WI). The specificity of biotinylated-m909 for human FR-β was established previously [33]. The mouse anti-mouse FR-β IgG2a mAb (F3) generated against the full-length protein has also been characterized elsewhere [24].

### Fluorescence microscopy

Acetone-fixed, frozen tissue sections or formaldehyde fixed cells were blocked with 5% BSA, stained with biotinylated m909 or unmodified F3, blocked with 10% human or mouse serum, stained with anti-CD68 mAb, blocked with 10% goat serum, and stained with Cy3-labeled F (ab’)_2_ fragments of a goat anti-mouse antibody or streptavidin-Cy-5 conjugate. All staining steps were followed by washing in PBS. Nuclei were stained with Hoechst Blue. Monochrome images were digitally captured (Retiga 1300C, Q-Imaging, Burnaby, Canada) on a multi-channel fluorescence microscope (BX51, Olympus). Pseudo-color application, and image overlays were performed using NIH ImageJ software.

### Immunohistochemistry

Tissue samples were deparaffinized with 3 changes of xylene, rehydrated in a series of ethanol dilutions (100%, 95%, then 70% ethanol), and rinsed well in running distilled water. Slides then were placed in a preheated DAKO Target Retrieval buffer for 40 min, then cooled in the same buffer for 20 min, followed by a 5-min rinse in running distilled water. Sections were then incubated with 3% H_2_O_2_ in ethanol for 5 min, followed by a protein blocking step for 5 min. Slides were rinsed well with Tris-buffered saline containing 0.05% Tween 20 (TBST) wash buffer and incubated for 30 min at room temperature with 0.7 mg/mL biotinylated-m909. Sections then were rinsed with TBST wash buffer followed by addition of HRP-streptavidin. After a 10-min incubation at room temperature, the slides were washed with TBST. Finally, slides were incubated in 3,3′-diaminobenzidine for 5 min at room temperature, counterstained with Modified Schmidts’s Hematoxylin for 5 min, and rinsed in tap water for 3 min. The samples then were dehydrated through graded alcohols, cleared in 3 changes of xylene, and mounted with a permanent mounting media in preparation for analysis by a pathologist. A semi-quantitative grading of the percentage of positively staining area and staining intensity was determined via pixel counting.

### Cell culture

All cells (CHO-β, RAW264.7, isolated PBMCs) were cultured in folate-deficient media supplemented with 10% FBS and 1% penicillin/streptomycin. Cells were maintained at 37 °C in a 95% air, 5% CO_2_ atmosphere and split in a ~ 1:8 ratio upon reaching confluence.

### Animal husbandry

The prophylactic treatment study was conducted in accordance with Bolder BioPATH IACUC approved policies and procedures in accordance with *The Guide for the Care & Use of Laboratory Animals (8th Edition)*. All other animal procedures were approved by the Purdue Animal Care and Use Committee in accordance with guidelines from the National Institutes of Health. Mice were housed in a humidity and temperature controlled room on a standard 12 h light/dark cycle. Food and water were provided ad libitum.

### Human PBMC isolation, differentiation, and activation

Human blood was obtained with understanding and written consent of each subject. The study methodologies were approved by the Purdue Institutional Review Board. Human peripheral blood mononuclear cells (PBMCs) were freshly isolated using a Ficoll-Paque PLUS gradient separation kit according to the manufacturer’s protocol (GE Healthcare; Marlborough, MA). To induce differentiation of isolated PBMCs, cells were resuspended in PromoCell Monocyte Attachment Medium and plated at 1 million cells/cm^2^. After a 2-h incubation at 37 °C, medium was replaced with RPMI-1640 supplemented with either 100 ng/ml human rGM-CSF (M1 polarization) or 20 ng/mL rM-CSF (M2 polarization). Cells then were incubated for an additional 6 days at 37 °C. To activate the differentiated myeloid cells, 100 U/mL IFN-γ and 1 ng/mL LPS were added to M1 macrophages and 20 ng/mL IL-4 was added to M2 macrophages.

### Antibody binding affinities

CHO cells that were transduced to express human FR-β (CHO-β), isolated PBMCs, and differentiated PBMCs were incubated with m909. RAW264.7 (mouse macrophage) cells were incubated with F3. All cells were incubated for 30 min at room temperature, washed with PBS three times, and incubated with either anti-human IgG PE or anti-mouse IgG FITC for 30 min at room temperature. 7-AAD was used as a live/dead cell marker. Cells were analyzed via flow cytometry (Accuri C6, BD Bioscience) and evaluated using Accuri C6 software. Three independent binding studies were averaged to yield the final curve.

### Analysis of antibody-dependent cell cytotoxicity (ADCC)

When analyzing m909-mediated ADCC, human PBMCs were used as effector cells. Target cells were CHO-β cells, or differentiated M1- or M2-like macrophages. Initially, various effector to target cell ratios were tested. A 100:1 ratio then was used for all subsequent experiments. Target cells were incubated in various concentrations of m909 antibody for 30 min at room temperature. Effector cells were added and co-incubated with target cells for 24 h. Lysis of target cells was measured by CytoTox-ONE™ Homogeneous Membrane Integrity Assay kit according to the manufacturer’s instructions (Promega; Madison, WI). Data from at least three independent studies were combined and analyzed. For F3 ADCC, a commercially available murine ADCC Reporter Bioassay was used according to manufacturer’s instructions (Promega; Madison, WI). RAW 264.7 cells were used as the target cell line, and either F3 or the nonspecific control IgG m102.4 antibody was used as the opsonizing agent. Data from at least three independent studies were combined and analyzed.

### Analysis of complement dependent cell cytotoxicity (CDC)

CHO-β cells were plated at a density of 50,000 cells/well into 96-well plates. m909 was added first, followed by the addition of human plasma diluted in RPMI1640 medium 10 min later. Cells were incubated for 3 h at 37 °C. Lysis of cells was measured by CytoTox-ONE™ Homogeneous Membrane Integrity Assay kit according to the manufacturer’s instructions (Promega; Madison, WI). Data from at least three independent studies were combined and analyzed.

### Thioglycollate model

Balb/c mice were injected intraperitoneally with 1 mL of a 3% *w*/*v* sterile thioglycolate medium (*n* = 6 per group). After 48 h, various concentrations of F3 antibody were administered via IP injection. Mice were euthanized 8, 24, 48, or 96 h later, and 10 mL ice cold PBS was injected into the peritoneum to facilitate collection of the peritoneal fluid. Peritoneal fluid was harvested with a syringe, and cells were pelleted by centrifugation at 400×*g* for 10 min at 4 °C. The cell pellet was resuspended in ice-cold RPMI1640 media and incubated for 30 min at room temperature with the following antibodies to determine immune cell types: PE/Cy7 anti-CD11b (M1/70), APC anti-CD49b (DX5), Alexa Fluor® 488 anti-Ly-6G (RB6-8C5), FITC anti-CD3 (17A2), PE anti-CD19 (1D3/CD19), Alexa Fluor® 647 anti-CD11c (N418), and PE goat anti-mouse IgG (poly4053). 7-AAD was used as a live/dead cell marker. Cells were analyzed via flow cytometry on an Accuri C6 (BD Bioscience) using Accuri C6 software for data acquisition and analyses.

### Arthritis prophylactic study

Bovine type II collagen (BBP) was dissolved in 0.01 N acetic acid at a concentration of 4 mg/mL. Equal volumes of collagen and Freund’s complete adjuvant with supplemental tuberculosis bacterium were emulsified until a bead of this material held its form when placed in water. On days 0 and 21, 6–7-week old male DBA/1 mice were anesthetized with isoflurane and administered 100 μl of the collagen mixture via intradermal injection. On day 18, prior to any evidence of paw swelling, mice were randomized into treatment groups based on body weight. F3 antibody (5 or 10 mg/kg), 10 mg/kg Enbrel, or vehicle control was administered IP 3× per week for a total of 8 doses. Arthritis score was evaluated for each paw using the following scoring system: 0 = no signs of arthritis, 1 = swelling and/or redness of the paw or one digit, 2 = two joints involved, 3 = more than two joints involved, and 4 = severe arthritis of the entire paw and all digits. Scores for each of the four paws were added to give the final arthritis score (0 to 16).

### Arthritis restorative study

Arthritis was induced in mice exactly as described above. Prior to evidence of paw swelling, mice were randomized into treatment and control groups. Body weight, arthritis score and paw swelling, as measured by calipers, were immediately recorded and then measured every other day for the healthy control group (designated day 0 for the healthy control). For the treatment groups, various concentrations of F3 antibody were administered IP 2× per week for a total of 4 doses once each individual mouse reached an average arthritis score value of ~ 2 (first day of treatment for each individual mouse was designated day 0). Weight, arthritis score, and paw swelling, as measured by calipers, were recorded every other day. Eighteen days post-antibody treatment, the presence of activated macrophages was determined using the FR-targeted radioimaging agent (^99m^Tc-EC20). Briefly, 1 mL of 15 mCi/mL sodium pertechnetate was added to 0.1 mg of EC20 and heated at 100 °C for 15 min. After diluting with saline, 100 μL of ^99m^Tc-EC20 (150 μCi; ∼75 nmol/kg of EC20) was injected via IP. After 4 h, mice were anesthetized with 3% isoflurane and imaged using a U-SPECT-II/CT/PET imaging system (MiLabs, Utrecht, Netherlands). A μCT 40 (Scanco, Wayne, PA) was then used to collect computed tomography (CT) images for bone density and quality. The feet were immersed in PBS during scanning to prevent dehydration. ImageJ software with the BoneJ addition was used to analyze the CT scans for bone density, total volume (TV), relative bone volume (BV/TV), trabecular thickness (Tb.Th), trabecular spacing (Tb.Sp), connectivity, connectivity density (Conn.D), and structure model index (SMI). Volumes of interest containing the medial metacarpal were isolated and analyzed using a custom script (see Additional file 2).

After imaging, mice were sacrificed, blood was collected, and joints were excised. Joint tissue then was fixed in 4% formaldehyde, decalcified, and embedded in paraffin. After 5-μm thick sectioning, tissues were mounted on slides and stained using hematoxylin–eosin (HE). Additional joint tissue was stained with anti-mouse-Armenian hamster IgG Alexa Fluor® 488 anti-CD11b antibody (10 μg/mL), anti-mouse-armenian hamster IgG Alexa Fluor® 647 anti-CD49b (5 μg/mL), anti-mouse-rat IgG2b Alexa Fluor® 488 anti-CD3 (10 μg/mL), anti-mouse-rat IgG2a Alexa Fluor® 647 anti-CD19 (5 μg/mL), anti-mouse-rat IgG2b Alexa Fluor® 594 anti-GR1 (2.5 μg/mL), anti-mouse-rat IgG2a Alexa Fluor® 594 anti-F4/80 (5 μg/mL), APC dye conjugated anti-FR-β antibody (100:1 dilution), and DAPI. These stained tissues were analyzed via fluorescence microscopy.

### F3 biodistribution

Arthritis was induced exactly as described above. Once paw swelling was evident (arthritis score = ~ 2), 50 mg/kg F3 antibody conjugated to AF647 was administered via IP injection. Whole animal fluorescence was measured at various times using a Caliper IVIS Lumina II Imaging station (PerkinElmer, Santa Clara, CA) coupled to an ISOON5160 Andor Nikon camera equipped with Living Image Software Version 4.0 (Oxford Instruments, Concord, MA).

## Results

### Accumulation of FR-β-positive macrophages in human inflammatory/autoimmune lesions

**Fig. 1:**
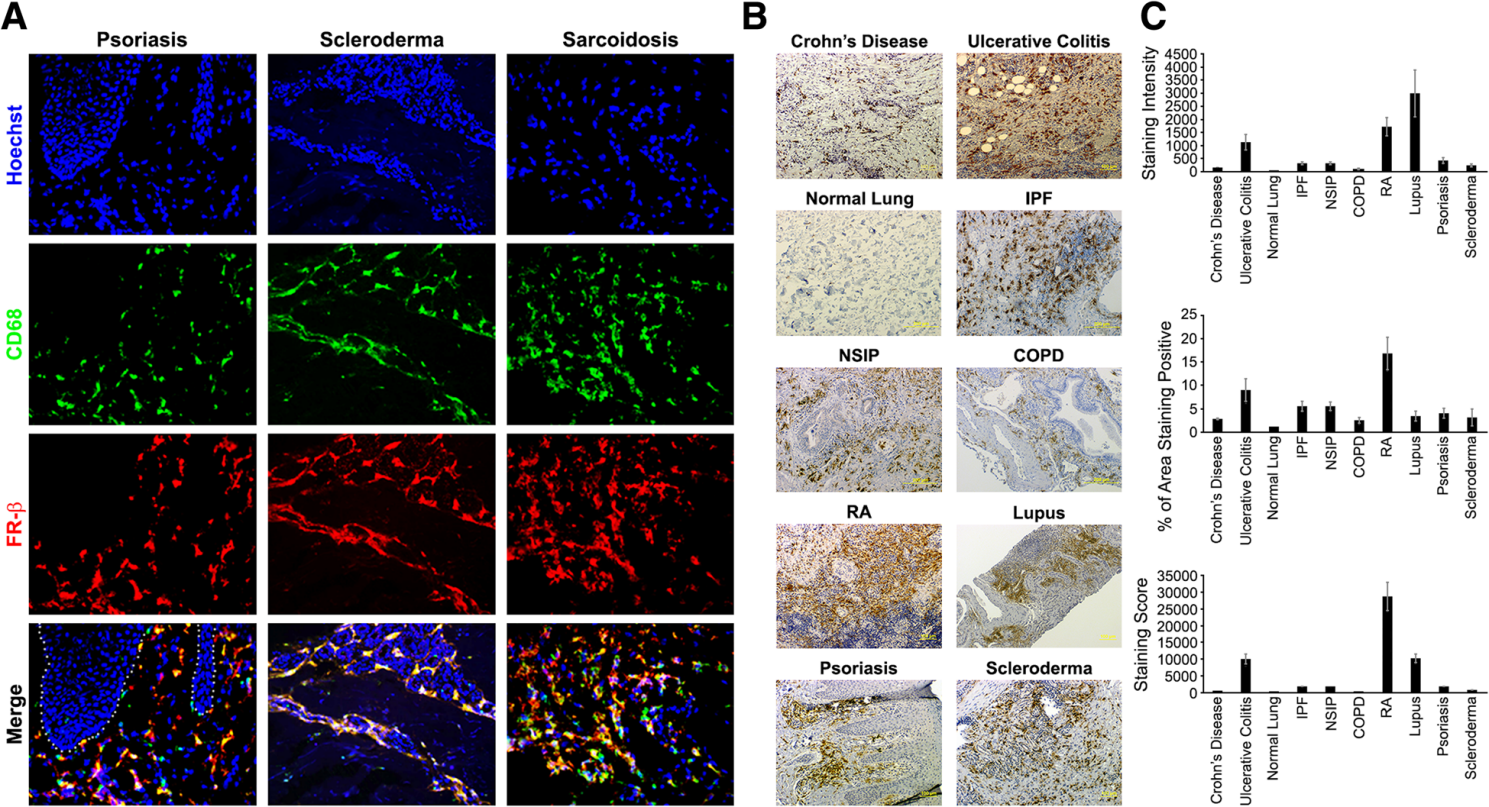
Staining of FR-β-positive macrophages in tissues from patients with inflammatory/autoimmune diseases. **a** Tissue sections from patients with psoriasis, scleroderma, and sarcoidosis were stained with Hoescht dye (DNA, blue channel), anti-CD68 antibody followed by Cy3 labeled secondary antibody (macrophages, false colored green), and anti-FR-β antibody followed by Cy5 labeled secondary antibody (FR-β, false colored red). Tissue sections were imaged via fluorescent microscopy at 400×. **b** Representative images of IHC staining for FR-β in human tissue sections from patients with Crohn’s disease (*n* = 12), ulcerative colitis (*n* = 9), normal lung (*n* = 31), idiopathic pulmonary fibrosis (IPF; *n* = 53), nonspecific interstitial pneumonia (NSIP; *n* = 32), chronic obstructive pulmonary disease (COPD; *n* = 40), rheumatoid arthritis (RA; *n* = 30), systemic lupus erythematosus (Lupus; *n* = 6), psoriasis (*n* = 8), and scleroderma (*n* = 4). **c** Images were analyzed and the mean staining intensity (gray value), percentage of the total area staining positive (% of pixels above threshold), and staining score (staining intensity multiplied by % of area staining positive) were plotted. Error bars represent SEM for all panels

Prior to testing the therapeutic benefit of eliminating FR-β-positive macrophages in animal models of inflammatory diseases, it was important to establish the extent of FR-β-positive macrophage infiltration in various human autoimmune/inflammatory diseases. For this purpose, a previously characterized [32, 33] human anti-human FR-β mAb (m909) that can bind either M1- or M2-FR-β-expressing macrophages [14, 15, 18] was initially utilized to stain tissues from three accessible inflammatory diseases via skin biopsy, namely, psoriasis, scleroderma, and sarcoidosis. As shown in Fig. 1a, FR-β staining was abundant in all three pathologies and consistently co-localized with the macrophage marker CD68 [34], i.e., confirming that FR-β is restricted to the macrophage/monocyte population [14, 15]. Next, m909 was used to stain a larger panel of tissue sections from many other human inflammatory and autoimmune diseases. As shown in Fig. 1b, all diseases tested exhibited prominent FR-β staining (additional IHC images can be viewed in Additional file 1: Figure S1-S23), suggesting that accumulation of FR-β-positive macrophages constitutes a common characteristic of most if not all autoimmune diseases. Although variability in FR-β staining score was observed among different diseases (Fig. 1c), with rheumatoid arthritis (RA) showing the highest and COPD displaying the lowest, all inflammatory diseases were found to exhibit FR-β staining. Therefore, we hypothesized that FR-β might constitute a useful marker for imaging and/or treating this class of diseases.

### Characterization of anti-FR-β mAb binding and ADCC activity in vitro

**Fig. 2:**
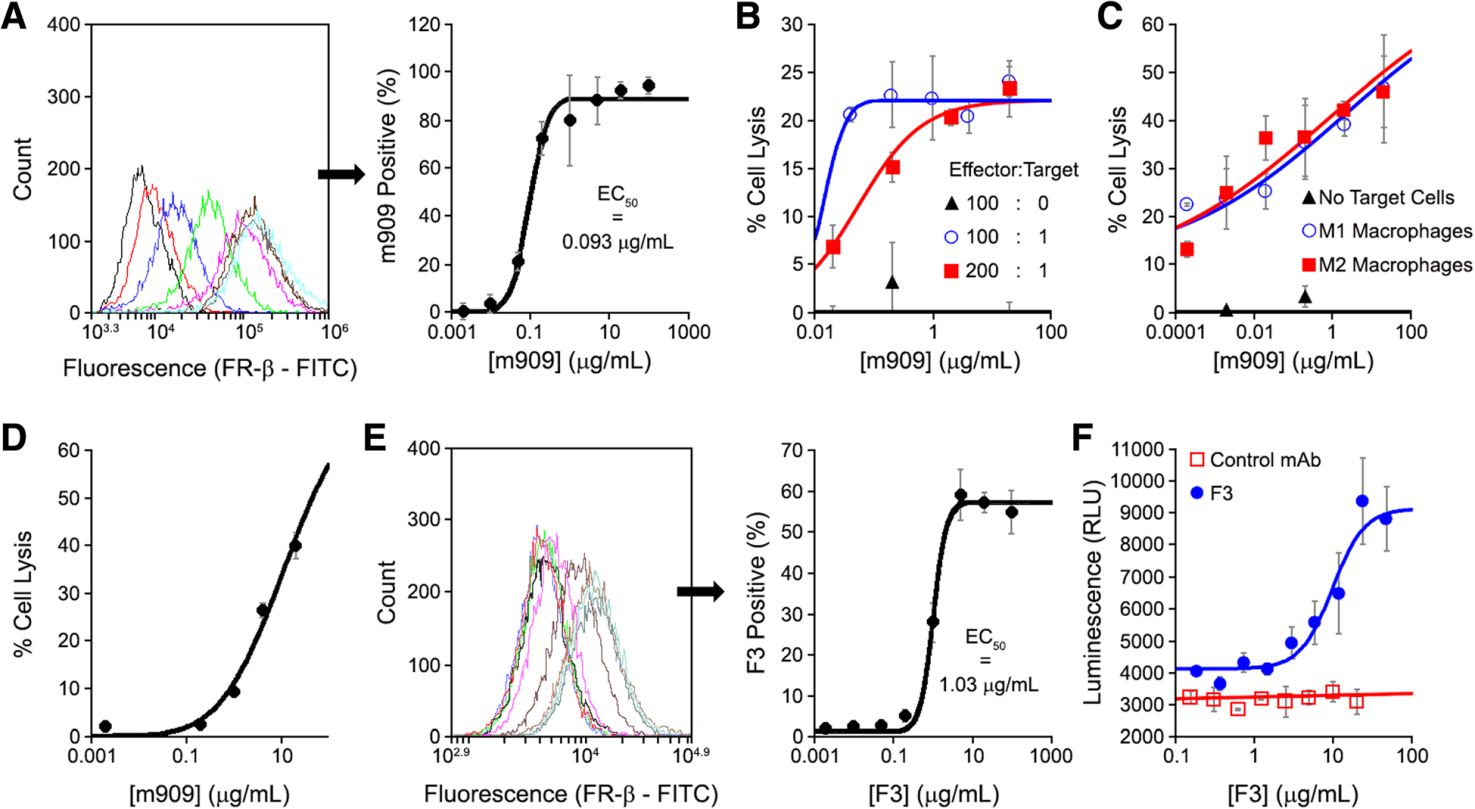
Characterization of anti-human FR-β (m909) and anti-mouse FR-β (F3) mAbs. **a** Shift in fluorescence intensity of CHO-β cells determined in the presence of increasing concentrations of m909 by flow cytometry. **b** m909-mediated ADCC of CHO-β cells incubated in the presence of different effector (human PBMCs) to target cell (CHO-β cells) ratios. ADCC-mediated CHO-β cell lysis was measured after 24 h by a LDH release assay. **c** m909-mediated ADCC of M1- or M2-like macrophages by PBMC effector cells. Isolated human PBMCs were differentiated into M1- or M2-like macrophages and activated to express FR-β as described in the Materials and methods section. After macrophage opsonization with m909 (target cells), freshly isolated human PBMCs (effector cells) were added to the macrophage culture, and ADCC-mediated lysis was measured after 24 h via LDH release assay. **d** m909-mediated CDC of CHO-β cells incubated in the presence of fresh human plasma. CHO-β cells, m909, and human serum diluted in RPMI1640 medium were incubated for 3 h at 37 °C. Cell lysis was determined via LDH release assay. **e** Shift in fluorescence intensity of RAW 264.7 cells determined in the presence of increasing concentrations of F3 by flow cytometry. **f** F3-mediated ADCC of RAW 264.7 cells using a commercially available murine ADCC luminescence reporter bioassay. All error bars represent standard deviation for all panels

Although multiple approaches might have been exploited for selective elimination of FR-β-positive macrophages, we elected to explore the use of an anti-FR-β mAb to mediate antibody-dependent cell cytotoxicity (ADCC) as an initial therapy for the above pathologies. As shown in Fig. 2a, m909 bound to FR-β-positive CHO cells (CHO-β cells) with half-maximal binding at 0.09 μg/mL (~ 0.6 nM). Moreover, in the presence of freshly isolated human peripheral blood mononuclear cells (PBMCs), m909 was able to mediate ADCC of CHO-β cells with an EC_50_ of ~ 0.01–0.06 μg/ml, depending on the effector to target cell ratio (Fig. 2b), i.e., similar to positive controls (Additional file 1: Figure S24). m909 was also able to promote ADCC in a more relevant assay of inflammation in which human PBMCs were differentiated and proliferated in vitro into M1- or M2-like macrophages and then activated to mimic the phenotypes observed in autoimmune lesions (see the “Materials and methods” section). As shown in Additional file 1: Figure S25, m909 bound to ~ 10%, 63%, and 82% of the total population of undifferentiated PBMCs and M1- and M2-differentiated macrophages, respectively. Moreover, as shown in Fig. 2c, increasing concentrations of m909 promoted little if any ADCC-mediated death of undifferentiated PBMCs (unactivated, low FR-β-expressing controls; black line), whereas similar increases in m909 caused dose-dependent ADCC when incubated with either M1- or M2-like macrophages (blue and red lines, respectively). Although no statistically significant difference in m909-mediated ADCC was observed between M1- and M2-like macrophages, ADCC of both activated macrophages was significantly different from undifferentiated PBMCs at every concentration tested (Additional file 1: Figure S26). Furthermore, results were statistically similar when ADCC was assayed in the presence of fetal bovine or human serum (Additional file 1: Figure S27), suggesting m909-mediated ADCC is not suppressed by serum as seen with other antibodies, at least in this assay system [35]. Finally, in addition to ADCC, m909 was also found to promote complement-dependent cytotoxicity (CDC) of CHO-β cells when incubated in the presence of human plasma but absence of PBMCs (Fig. 2d), mediating lysis of 50% of CHO-β cells at ~ 10 μg/ml m909. Taken together, these data demonstrate that m909 can target FR-β-positive macrophages and promote their opsonization, thereby mediating their elimination through both ADCC and CDC mechanisms.

We next undertook studies to determine whether the anti-mouse FR-β mAb counterpart of m909 (F3) behaves sufficiently similar to m909 in order to qualify it as a surrogate for in vivo evaluation of an anti-FR-β mAb in murine autoimmune/inflammatory disease models. Figure 2e demonstrates that F3 binds RAW264.7 cells (an FR-β expressing murine macrophage cell line; [36]) with half maximal binding at 1.03 μg/mL, i.e., ~ 10-fold weaker than m909. Figure 2f shows that the ability of F3 to promote ADCC of RAW 264.7 cells is also approximately tenfold weaker than m909. Therefore, successful elimination of FR-β-positive macrophages by F3 in murine models of autoimmune diseases would strongly support m909 as a potential therapeutic for treating autoimmune disease symptoms in humans.

### Evaluation of anti-FR-β mAb efficacy in a thioglycollate model of inflammatory disease

**Fig. 3:**
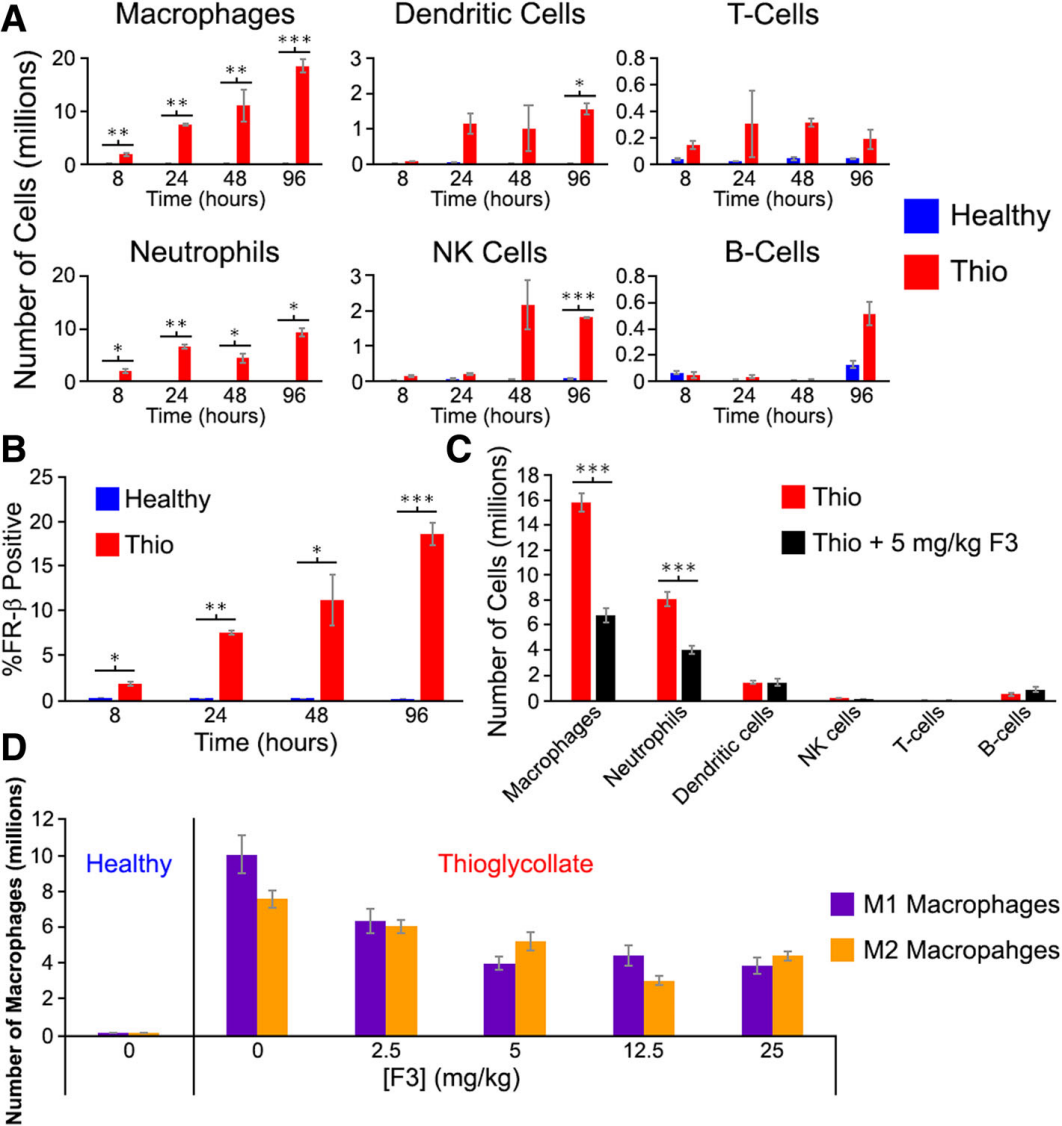
Effect of anti-mouse FR-β mAb (F3) treatment on peritoneal immune cell populations following induction of peritonitis with thioglycollate. **a** Mice were injected intraperitoneally with thioglycollate (Thio), and the immune cell populations were quantitated at the indicated times by flow cytometry. **b** The percentage of peritoneal macrophages that are FR-β positive is shown as a function of time in both healthy and thioglycollate-treated mice. **c** Thioglycollate was administered to mice as in panel **a**, and F3 (5 mg/kg) was injected intraperitoneally 48 h later. After an additional 48 h, the peritoneal cavity was washed and different immune cell populations were quantitated via flow cytometry. **d** Mice were treated and peritoneal fluid was examined as in panel **c** except various concentrations of F3 was administered. For all graphs, error bars represent SEM and *, **, and *** denote *p* values < 0.05, < 0.005, 0.0005, respectively for all panels

As an initial test to determine whether F3 promotes elimination of FR-β-positive macrophages in vivo, we examined the impact of F3 administration on a general model of inflammation in which thioglycollate was injected into the peritoneal cavities of mice to induce infiltration of immune cells [37, 38]. As shown in Fig. 3a, analyses of peritoneal lavages at different time points post-thioglycollate administration revealed a continuous influx of macrophages and neutrophils, followed by accumulation of dendritic and NK cells, and culminating with infiltration of T- and B-cells (Additional file 1: Figure S28). When the percentage of FR-β-positive (activated) macrophages was examined, healthy mice were observed to exhibit essentially no FR-β expressing myeloid cells, while the peritoneal fluid from mice treated with thioglycollate contained substantial numbers of these cells. More importantly, the fraction of macrophages that was FR-β^+^ was found to increase as inflammation progressed (Fig. 3b). Except for a small fraction of neutrophils, no other immune cell type displayed measurable expression of FR-β (Additional file 1: Figures S29-S30).

Next, to determine if administration of an anti-FR-β mAb might impact the numbers of infiltrating immune cells, F3 (5 mg/kg) was administered to mice 48 h after injection of thioglycollate, and after an additional 48 h, the numbers of immune cells isolated from the peritoneal cavity were analyzed. As shown in Fig. 3c and Additional file 1: Figures S31-S32, the numbers of both macrophages and neutrophils declined significantly upon treatment with F3, and this reduction was strongly dependent on F3 concentration with maximal efficacy achieved between 5 and 12.5 mg/kg (Fig. 3d). The reduction in numbers was essentially the same for both M1 and M2 macrophages with a decrease of ~ 50% at doses > 5 mg/kg. In contrast, the levels of dendritic, NK, T cells, and B cells did not change significantly, at least at this early time point. Taken together, these data confirm that administration of an anti-FR-β mAb can promote a concentration- and time-dependent elimination of FR-β-positive myeloid cells. However, whether this suppression of activated macrophages would impact the etiology of a more relevant inflammatory disease model would require further investigation.

### Murine model of RA exhibits similar FR-β expression to human RA

**Fig. 4:**
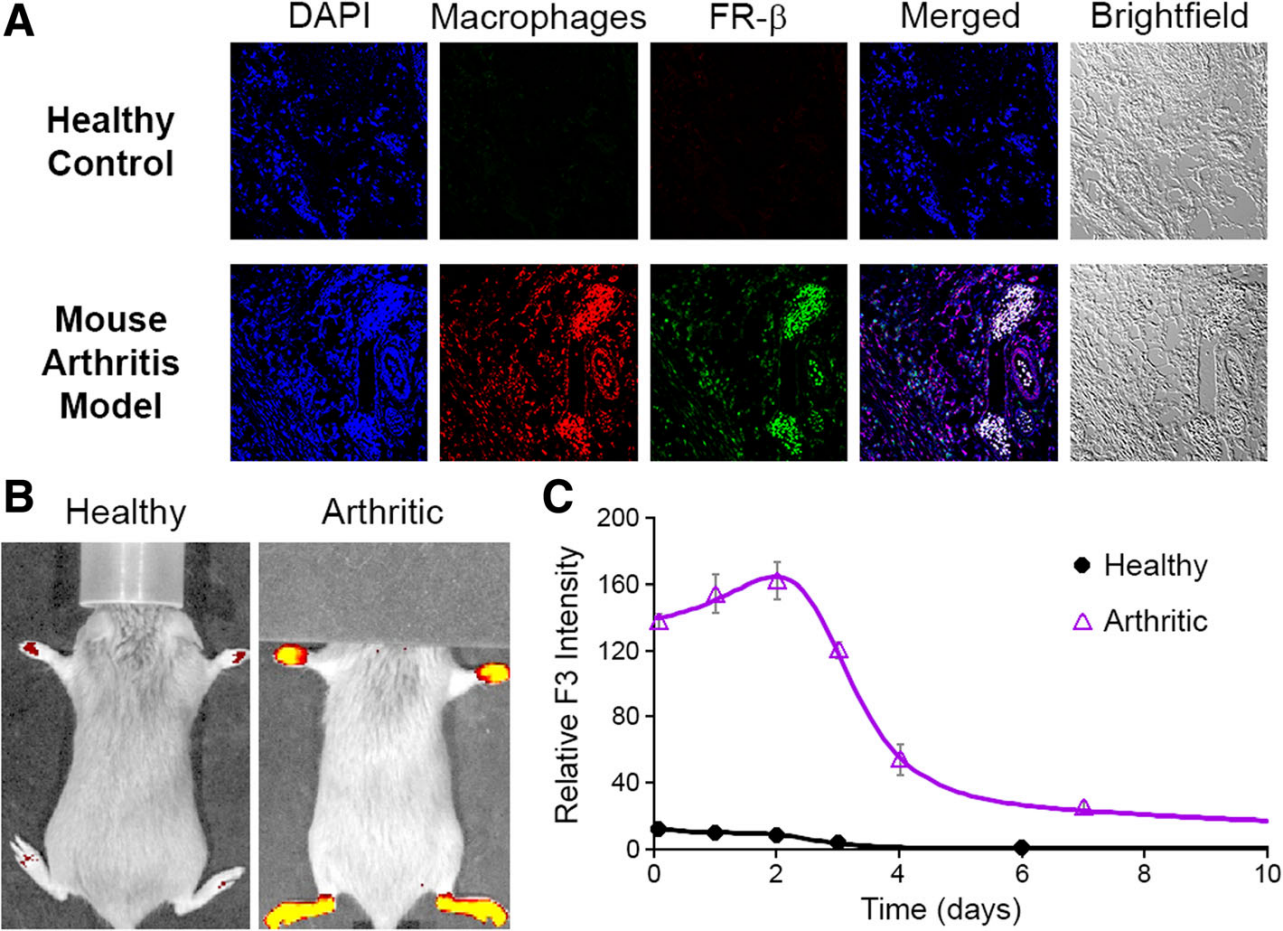
Characterization of F3 binding to FRβ-expressing macrophages in arthritic joints**. a** Two weeks after initial appearance of symptoms in collagen-induced arthritic mice, arthritic joints were sectioned, stained with DAPI dye (nucleus, blue), AF594 dye conjugated anti-F4/80 antibody (macrophage marker, red), and APC dye conjugated anti-FR-β antibody F3 (FR-β, false colored green), and then imaged via fluorescent microscopy. **b** Mice with collagen-induced arthritis were injected intraperitoneally with AF647-conjugated F3 (50 mg/kg) when the average arthritis score of all 4 paws reached ~ 2. Whole animal fluorescence was imaged at various times post-F3 injection (representative image at 2 days post F3 administration) and paw fluorescence was quantitated as indicated (**c**)

Because rheumatoid arthritis (RA) was found to be the inflammatory disease characterized by the largest infiltration of FR-β-positive macrophages in humans (Fig. 1b), we next examined whether the collagen-induced model of RA in mice might be similarly characterized by infiltration of FR-β expressing macrophages. As shown in Fig. 4a, F4/80 expressing macrophages were abundantly present in the synovial membranes of joints from arthritic mice, but not in corresponding joints from healthy mice. Moreover, the vast majority of macrophages isolated from collagen-induced arthritic mice co-stained positive for FR-β, i.e., similar to RA in humans. These data confirm that collagen-induced arthritic mice can recapitulate the most relevant properties of RA for validating F3 as a surrogate for predicting m909 behavior in humans.

To test whether F3 can accumulate in arthritic joints where it can mediate ADCC/CDC, AlexaFluor 647-labeled F3 was injected intraperitoneally into collagen-induced arthritic mice and the location of F3 fluorescence was imaged as a function of time. As shown in Fig. 4b and Additional file 1: Figure S33, AlexaFluor 647 fluorescence was concentrated in the arthritic feet/ankles of diseased mice, but not healthy mice, consistent with binding to the targeted cell population. Moreover, retention of this fluorescence in affected feet for > 3 days suggests that F3 could co-localize with FR-β-positive macrophages long enough to mediate ADCC and that dosing of F3 every 2–3 days should maintain the mAb near its maximum level within the inflamed feet.

### Efficacy of F3 in a collagen-induced arthritis murine model

**Fig. 5:**
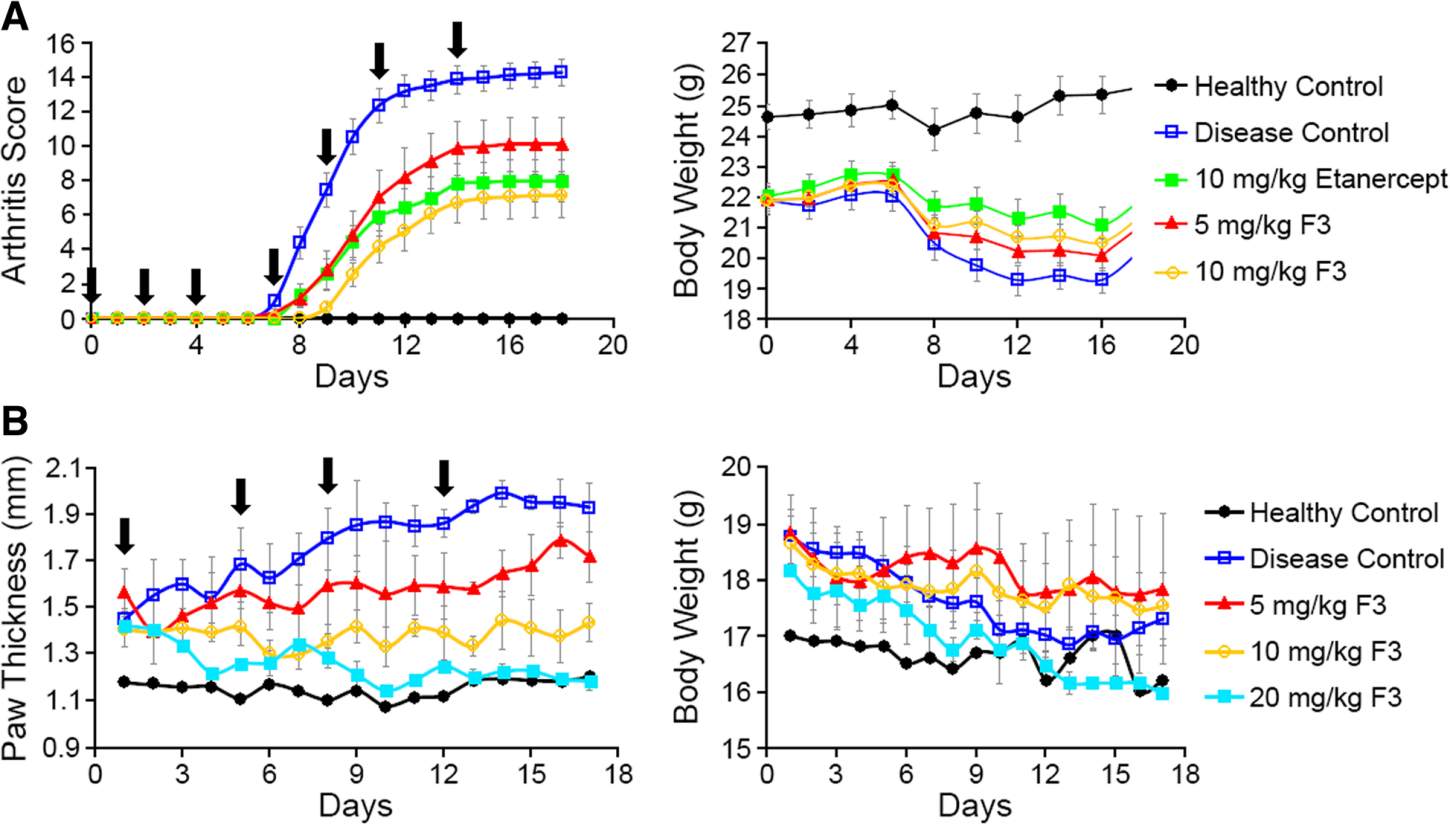
Evaluation of the ability of different concentrations of F3 to treat collagen-induced arthritis in mice. Arthritis was induced as described above (*n* = 12 per group), and either F3 (5 or 10 mg/kg) or etanercept (10 mg/kg) was injected intraperitoneally 18 days later (day 0 on graph) before signs of arthritis were evident. Treatments were administered 3×/week (arrows) for a total of 8 doses. **a** Arthritis scores and body weights were determined every other day. **b** The study in panel **a** was repeated except administration of the indicated concentrations of F3 (2×/week; see arrows) was initiated only after arthritis scores reached ~ 2. Error bars represent SEM for all panels

With an optimal dosing frequency of F3 determined, a multi-dose prophylactic study was then conducted to determine the efficacy of F3 in a less challenging collagen-induced arthritis model. For this purpose, mice (*n* = 12 mice per group) were treated with various concentrations of F3 or 10 mg/kg etanercept (Enbrel, i.e., an FDA-approved biologic for treatment of moderate to severe RA) 18 days after induction of arthritis but ~ 1 week prior to onset of overt paw inflammation. As shown in Fig. 5a and Additional file 1: Figure S34, mice treated with anti-FR-β prophylactic therapy exhibited lower arthritis scores in all treatment groups, with 10 mg/kg dose displaying the best response. Importantly, arthritis scores of both 10 mg/kg F3 and etanercept treatment groups were significantly lower than disease controls but not significantly different from each other (Additional file 1: Figure S35). Additionally, no significant difference in body weight was observed between any treatment groups (Fig. 5a and Additional file 1: Figures S36-S37), suggesting none of the therapies caused gross toxicity to the mice.

We next evaluated F3 in a more challenging therapeutic model of arthritis, where F3 was administered when joint swelling in all paws yielded an average arthritis score of ~ 2. As shown in Fig. 5b and Additional file 1: Figures S38-S40, paw thickness was reduced relative to disease control at all concentrations tested, with the 20 mg/kg treatment group exhibiting a decrease in absolute paw thickness during continued therapy that ultimately led to its convergence with the healthy controls. Similar to the prophylactic study, body weight of all arthritic mice declined over time (Fig. 5b and Additional file 1: Figure S41); however, weight loss in the treatment groups (Additional file 1: Figure S42) was not significantly different from disease controls, indicating that even 20 mg/kg F3 does not cause gross toxicity.

**Fig. 6:**
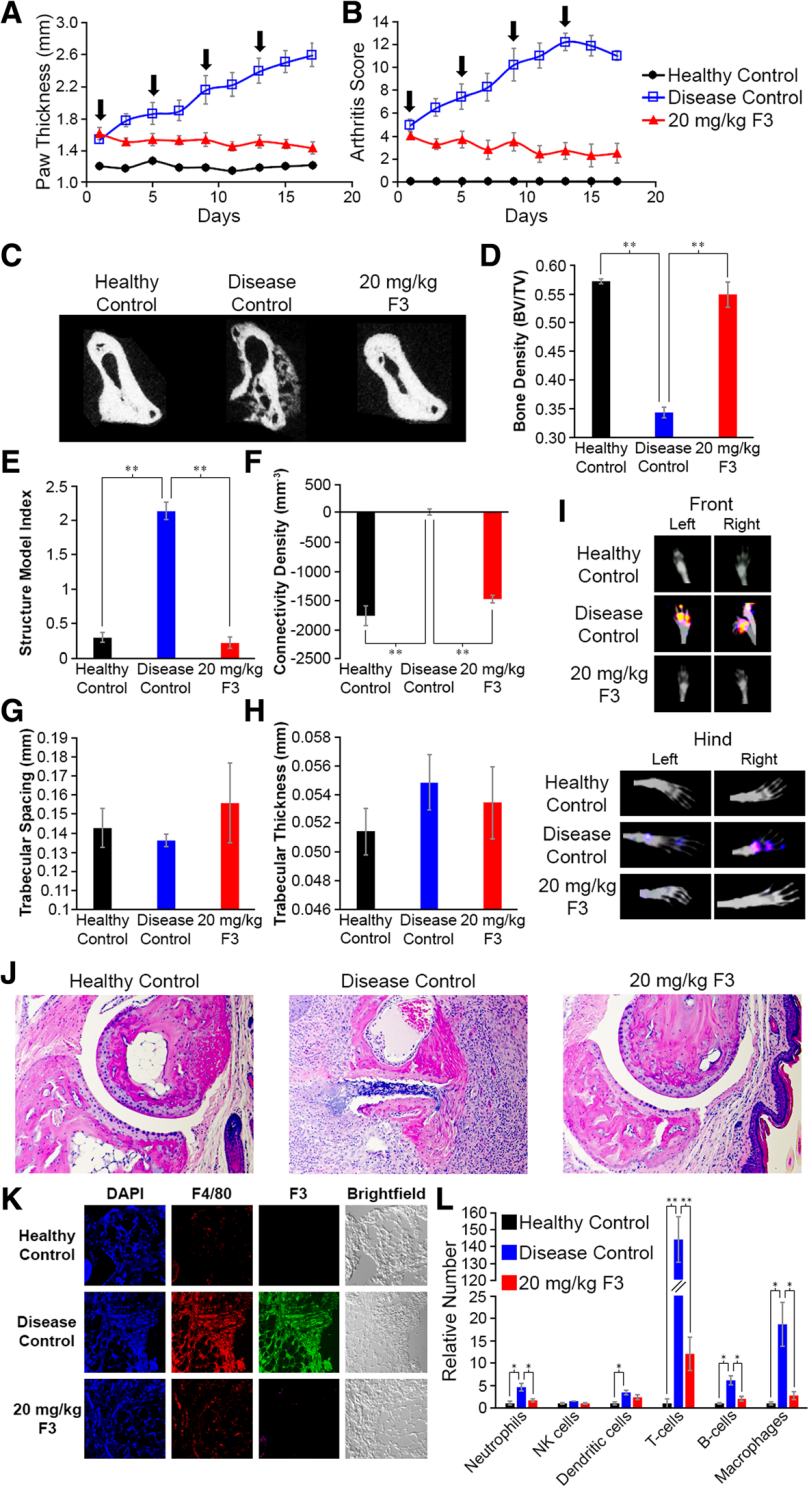
Detailed examination of therapeutic impact of optimal F3 dosing on collagen-induced arthritis in mice. Arthritis was induced as described above (*n* = 10 per group), and 20 mg/kg F3 was injected intraperitoneally once arthritis scores reached ~ 4. Treatments were administered 2×/week (arrows) for a total of 4 doses. **a** Paw thickness and **b** arthritis scores were determined every other day. **c** CT images of paws were obtained, and a representative cross section of the thumb metacarpal bone is shown for each group. **d** Morphometric bone analyses including bone density, **e** structure model index, **f** connectivity density, **g** trabecular spacing, and **h** trabecular thickness of medial metacarpals were quantitated as described in the Materials and methods section. **i** Mice were injected on day 18 with a ^99m^Tc-folate receptor-targeted imaging agent (EC20; 75 nmol/kg) and imaged 4 h later. Representative images are shown, and quantitation of radioactive intensity in the paws is plotted. **j** At the end of the study (day 18), sections of joint tissue were H&E stained. Representative images are shown for each treatment group. Black arrows denote immune cell infiltration. **k** Similar joint tissue sections were stained with desired markers for nuclei (DAPI, blue channel), macrophages (F4/80, red channel), FR-β (F3, green channel), and imaged by fluorescent microscopy. Additional images of different immune cell types can be viewed in SI Figs. BN - BS. **l** Staining intensity for each immune cell type was quantitated for healthy (black), untreated RA (blue), and F3-treated RA (red) mice. Error bars represent SEM and *, **, and *** denote *p* values < 0.05, < 0.005, and 0.0005, respectively for all panels

To obtain more detailed information on the mechanism by which F3 treats collagen-induced arthritis in mice, arthritic mice were allowed to progress until their average arthritis scores from all four paws exceeded four and at that time were treated 2×/week with 20 mg/kg F3 or left untreated. As seen in Fig. 6a and b, F3 therapy again suppressed disease-induced increases in both paw thickness (Fig. 6a and Additional file 1: Figure S43-S45) and arthritis score (Fig. 6b and Additional file 1: Figures S46-S47), resulting in the previously noted decreases in both parameters as therapy proceeded. Moreover, as shown in the micro-CT images of Fig. 6c, F3 treatment also prevented the dramatic decrease in bone density characteristic of the untreated disease controls. Indeed, more detailed computer analysis of the micro-morphological characteristics of the affected bones demonstrated that F3 treatment prevents (i) a prominent decrease in bone density (Fig. 6d), (ii) a marked increase in structure model index (Fig. 6e), and (iii) a dramatic increase in connectivity density (Fig. 6f). Moreover, bone properties not affected by disease progression such as trabecular spacing (Fig. 6g) and trabecular thickness (Fig. 6h) were also not affected by F3 treatment (Additional file 1: Figure S48), suggesting that F3 effects only disease-related bone changes.

To assess the number of FR-β-expressing cells remaining in the feet of affected mice, mice were imaged 4 days after the last treatment with a folate receptor targeted radioimaging agent (^99m^Tc-EC20). As shown in Fig. 6i and Additional file 1: Figure S49, significant uptake of ^99m^Tc-EC20 was observed in the untreated RA animals, but not in the healthy or F3-treated mice. After euthanasia, mice were resected and thin sections of their inflamed feet/ankles were scrutinized using histological and molecular staining techniques. As shown in Fig. 6j and Additional file 1: Figure S50-S53, H&E staining showed prominent immune cell infiltration in disease controls that was absent in F3-treated animals and healthy controls. When parallel sections were stained with fluorescently labeled antibodies for different immune cell types (e.g., neutrophil, NK cell, macrophage, dendritic cell, T cell, and B cell numbers), the relative number of immune cells, especially T cells and macrophages, was increased in the disease controls [39], but greatly suppressed in F3-treated tissues (Figs. 6k, l and Additional file 1: Figures S54-S59). Curiously, some cell types that do not express FR-β (e.g., T cells and B cells) were also significantly reduced following F3 therapy (Fig. 6k, l, Additional file 1: Figures S60 and S61). This result suggests that elimination of FR-β-positive macrophages by F3 suppresses recruitment or retention of other immune cells required to maintain the inflammatory milieu.

Lastly, body weight and a complete blood count were measured to identify any signs of gross toxicity. No significant difference in body weight (Additional file 1: Figures S62-S64) or any blood count value, except mean corpuscular hemoglobin (Additional file 1: Figures S65-S66), was observed among any of the treatment groups. Taken together, these data indicate that even at 20 mg/kg, F3 did not elicit any gross toxicity that would discourage its further development. Indeed, a recent study in which FRβ-positive monocytes were selectively depleted by up to 80% in human patients reported no obvious treatment-related toxicities [14], suggesting that treatments leading to reduction in FRβ-expressing monocytes can be well tolerated.

## Discussion

We have demonstrated that elimination of FR-β-positive macrophages with an anti-FR-β monoclonal antibody can reduce accumulation of inflammatory immune cells in collagen-induced arthritic joints and thereby treat the causes of this disease. Suppression of arthritis with an anti-FR-β monoclonal antibody is perhaps not surprising in view of the fact that FR-β-positive macrophages constitute the most inflammatory subset of this cell type [33], secreting a plethora of inflammatory stimuli (i.e., TNFα, IL-1, IL-6, IL-12, prostaglandins, leukotrienes, and reactive oxygen species) that enhance the inflammatory process including activation of other immune cells [7]. Indeed, considerable data demonstrate that neutralization of TNFα and other macrophage-generated cytokines promote the gradual disappearance of both activated macrophages and other immune cells from inflamed lesions [40–42]. In this respect, the inflammatory immune environment appears to behave like an orchestra, where departure of one section (e.g., the violins) precludes proper functioning of the remaining sections, causing the residual musicians to disband also [43, 44]. Because activated macrophages release so many cytokines that participate in maintenance of the inflamed state [7], the ability of their targeted elimination to concurrently suppress involvement of other inflammatory cells now seems intuitive.

It was informative that every inflammatory disease examined was characterized by accumulation of FR-β positive macrophages. Although the requirement for these macrophages to maintain the inflammatory milieu in Crohn’s disease, ulcerative colitis, idiopathic pulmonary fibrosis, nonspecific interstitial pneumonia, chronic obstructive pulmonary disease, rheumatoid arthritis, systemic lupus erythematosus, sarcoidosis, psoriasis, and scleroderma has not been established, successful therapy of several of these diseases, regardless of the drug used, has been shown to cause disappearance of FR-β-positive macrophages from the inflamed lesions [40, 41]. It is therefore tempting to speculate that an FR-β targeted therapy might contribute to resolution of many of these FR-β-enriched pathologies. While clodronate liposomes, which nonspecifically induce death of phagocytic cells, have been found to treat animal models of these diseases [9, 10], they simultaneously eradicate both quiescent (e.g., tissue resident) and activated/inflammatory macrophages. Because only the inflammatory subset expresses FR-β [14–31], an FR-β-targeted therapy might be anticipated to more specifically eliminate the disease-causing macrophages. The ability of m909 to selectively accumulate in the inflamed joints of the arthritic mice (Fig. 4b) suggests that it should largely avoid the killing of tissue-resident macrophages. Our previous demonstrations that folic acid-targeted imaging agents also selectively accumulate in inflamed lesions [25, 40, 41, 45–47] further suggest that folate-targeted drugs should also be specific in suppressing only the inflammatory subset of macrophages. Indeed, several folate-targeted anti-inflammatory drugs have demonstrated significant activity against animal models of autoimmune diseases without causing measurable off-target toxicities [36, 47, 48]. Based on all of these considerations, we conclude that m909 warrants further investigation as a potential broad-spectrum anti-inflammatory drug.

## Conclusions

The data presented here suggest that the vast majority of human inflammatory and autoimmune diseases are characterized by a marked increase in FR-β-expressing macrophages. When these macrophages are specifically targeted in animal models of inflammatory disease by a monoclonal antibody designed for ADCC, disease parameters significantly improved. Therefore, this strategy of selectively eliminating these activated macrophages along with the recently developed human anti-human FR-β monoclonal antibody (m909) provides a new mechanism for the potential treatment of multiple autoimmune and inflammatory diseases.

## Additional files

Additional file 1: Supplementary experimental data and analyses. (PDF 10858 kb)

Additional file 2: Custom script for bone morphometric analyses. (TXT 10 kb)

## Abbreviations

ADCC: Antibody-dependent cell cytotoxicity
BV: Bone volume
CHO- β: FR-β transfected CHO cells
Conn.D: Connectivity density
F3: Mouse anti-mouse FR-β specific monoclonal antibody
FR-β: Folate receptor beta
HE: Hematoxylin-eosin
m909: Human anti-human FR-β specific monoclonal antibody
mAb: Monoclonal antibody
PBMCs: Peripheral blood mononuclear cell
RA: Rheumatoid arthritis
SMI: Structure model index
Tb.Sp: Trabecular spacing
Tb.Th: Trabecular thickness
TBST: Tris-buffered saline containing 0.05% Tween 20
TV: Total volume
