# Supplementary material for: Depletion of activated macrophages with a folate receptor-beta-specific antibody improves symptoms in mouse models of rheumatoid arthritis
Source: Arthritis Res Ther. 2019 Jun 7;21:143. doi: 10.1186/s13075-019-1912-0 (PMC6555977; doi:10.1186/s13075-019-1912-0)
Supplement: Supplementary file 1 — Supplementary experimental data and analyses. (PDF 10858 kb) [file 13075_2019_1912_MOESM1_ESM.pdf]

Supplementary Information

Table of Contents

FR-β IHC images from human inflammatory and autoimmune diseases

- Crohn’s Disease .... SI Figure 1-3
- Ulcerative Colitis .... SI Figure 4-5
- Normal Lung .... SI Figure 6-8
- Idiopathic Pulmonary Fibrosis .... SI Figure 9-11
- Nonspecific Interstitial Pneumonia .... SI Figure 12-14
- Chronic Obstructive Pulmonary Disease .... SI Figure 15-17
- Rheumatoid Arthritis .... SI Figure 18-20
- Systemic Lupus Erythematosus .... SI Figure 21
- Psoriasis .... SI Figure 22
- Scleroderma .... SI Figure 23

Antibody Dependent Cell Cytotoxicity (ADCC)

- ADCC control antibody .... SI Figure 24
- Percentage of FR-β positive PBMCs and M1-/M2-differentitated macrophages .... SI Figure 25
- Statistical analysis of differences in ADCC of M1 and M2 macrophages (from **Fig. 2C**) .... SI Figure 26
- Effect of human serum on m909-mediated ADCC .... SI Figure 27

Thioglycollate Model

- Statistical analysis of differences in immune cell accumulation (from **Fig. 3A**) .... SI Figure 28
- Quantitation of FR-β positive immune cell subpopulations (from **Fig. 3B**) .... SI Figure 29
- Statistical analysis of differences in FR-β positive immune cells (from **Fig. 3B**) .... SI Figure 30
- Statistical analysis of differences in immune cell numbers (from **Fig. 3C**) .... SI Figure 31
- Statistical analysis of differences in macrophage numbers (from **Fig. 3D**) .... SI Figure 32

F3 Biodistribution

- Time dependence of F3 update in arthritic mice (from **Fig. 4B**) .... SI Figure 33

Table of Contents - Continued

Adjuvant-induced arthritis prophylactic treatment model

Arthritis score measurements (from **Fig. 5A**) ..... SI Figure 34
Statistical analysis of differences in arthritis scores (from **Fig. 5A**) ..... SI Figure 35
Body weight measurements (from **Fig. 5A**) ..... SI Figure 36
Statistical analysis of differences in body weight (from **Fig. 5A**) ..... SI Figure 37

Adjuvant-induced arthritis therapeutic model – dose finding study

Paw thickness measurements (from **Fig. 5B**) ..... SI Figure 38
Statistical analysis of differences in paw thicknesses (from **Fig. 5B**) ..... SI Figure 39
Images of mouse paws (from **Fig. 5B**) ..... SI Figure 40
Body weight measurements (from **Fig. 5B**) ..... SI Figure 41
Statistical analysis of differences in body weight (from **Fig. 5B**) ..... SI Figure 42

Adjuvant-induced arthritis therapeutic model – dose finding study

Images of mouse paws (from **Fig. 6**) ..... SI Figure 43
Paw thickness measurements (from **Fig. 6A**) ..... SI Figure 44
Statistical analysis of differences in paw thickness (from **Fig. 6A**) ..... SI Figure 45
Arthritis score measurements (from **Fig. 6B**) ..... SI Figure 46
Statistical analysis of differences in arthritis score (from **Fig. 6B**) ..... SI Figure 47
Statistical analysis of differences in morphometric bone parameters (from **Fig. 6D-6H**) ..... SI Figure 48
Measurements and statistical analysis of EC20 update (from **Fig. 6I**) ..... SI Figure 49
Images of H&E staining of joints (from **Fig. 6J**) ..... SI Figure 50-53
Immunofluorescence staining images of different immune cell populations from joints (from **Fig. 6K**) ..... SI Figure 54-59
Percent reduction of immune cell subpopulations (from **Fig. 6L**) ..... SI Figure 60
Statistical analysis of differences in immune cell types (from **Fig. 6L**) ..... SI Figure 61
Body weight graph (from **Fig. 6**) ..... SI Figure 62
Body weight measurements (from **Fig. 6**) ..... SI Figure 63
Statistical analysis of differences in body weight (from **Fig. 6**) ..... SI Figure 64
Complete blood count values (from **Fig. 6**) ..... SI Figure 65
Statistical analysis of complete blood count (from **Fig. 6**) ..... SI Figure 66

Human Inflammatory Disease IHC Staining

Crohn’s Disease

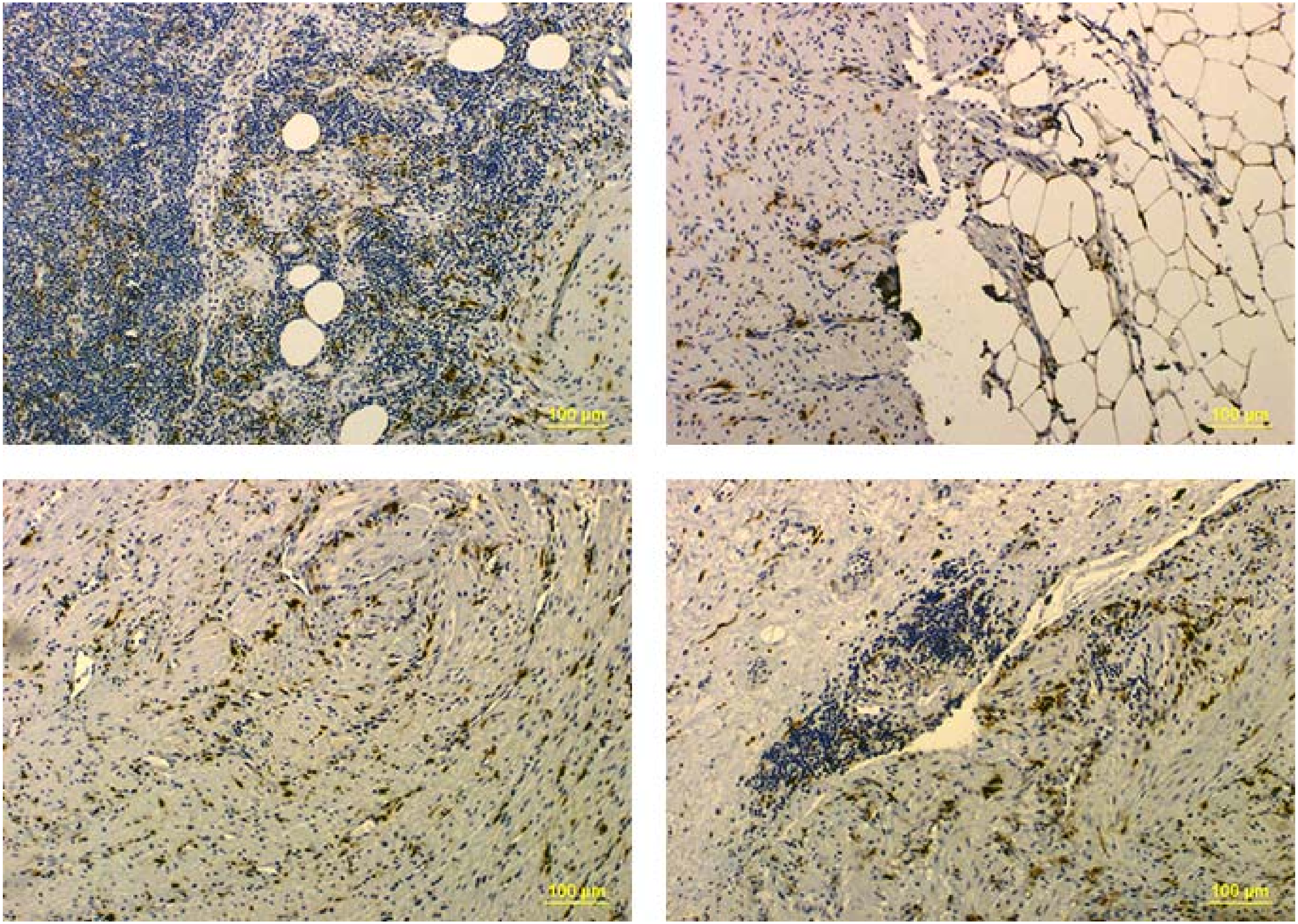

SI Figure 1. Immunohistochemistry staining of thin sections of colon from patients with Crohn's Disease. The indicated tissue sections were stained with m909, a human monoclonal antibody to human FR-β, that exhibits no cross-reactivity to other folate receptor isoforms.

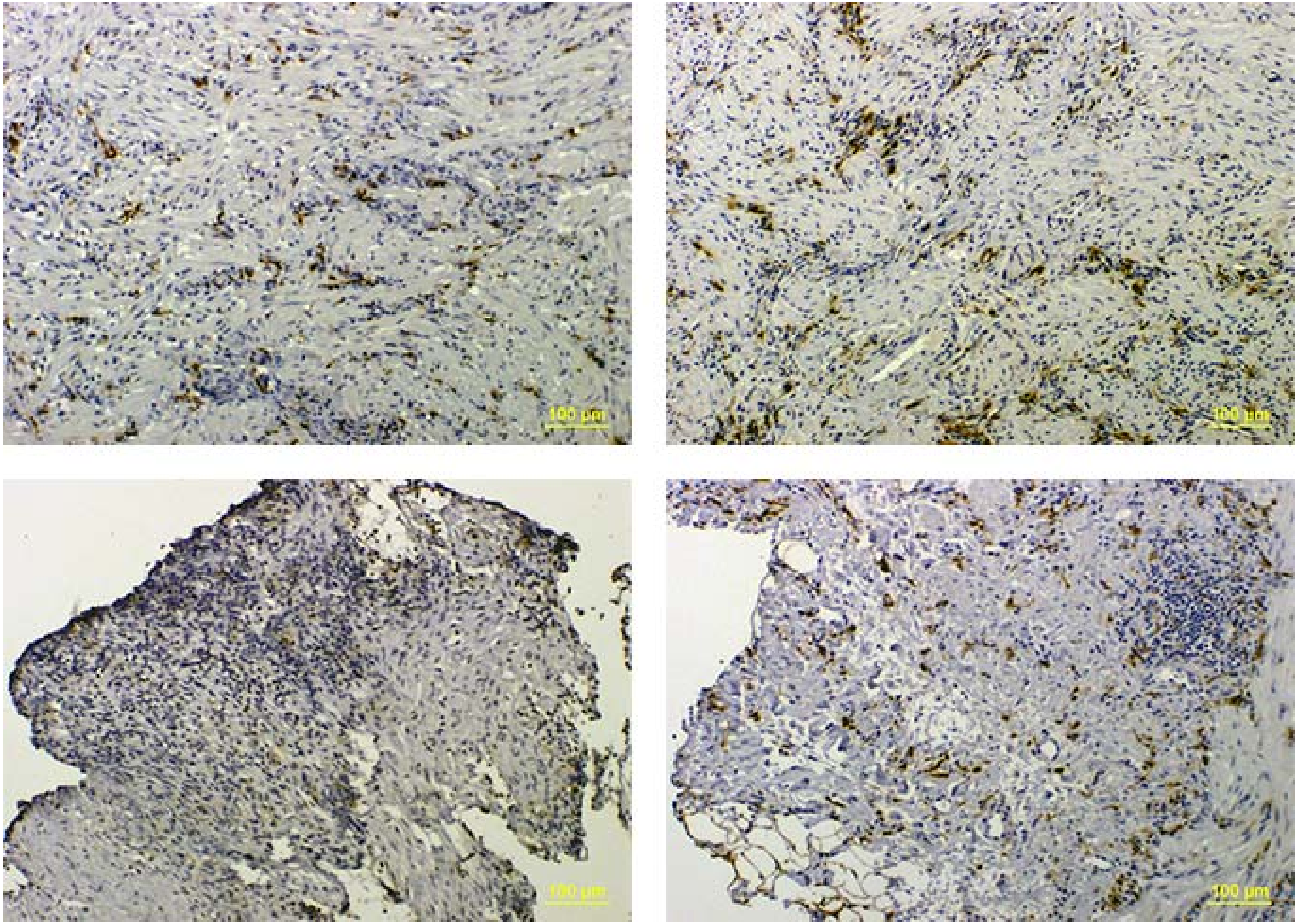

SI Figure 2. Immunohistochemistry staining of thin sections of colon from patients with Crohn's Disease. The indicated tissue sections were stained with m909, a human monoclonal antibody to human FR-β, that exhibits no cross-reactivity to other folate receptor isoforms.

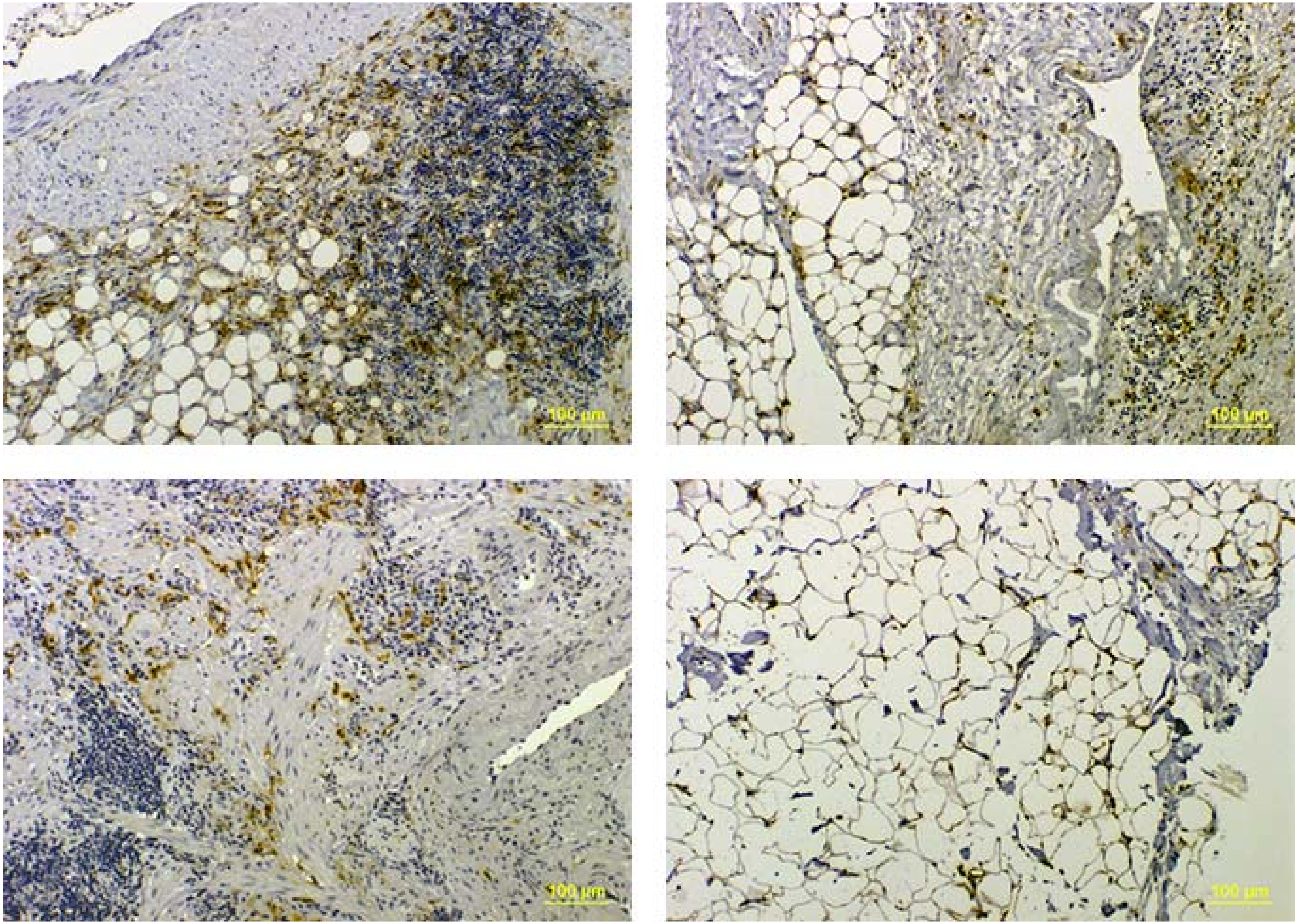

SI Figure 3. Immunohistochemistry staining of thin sections of colon from patients with Crohn's Disease. The indicated tissue sections were stained with m909, a human monoclonal antibody to human FR-β, that exhibits no cross-reactivity to other folate receptor isoforms.

Human Inflammatory Disease IHC Staining

Ulcerative Colitis

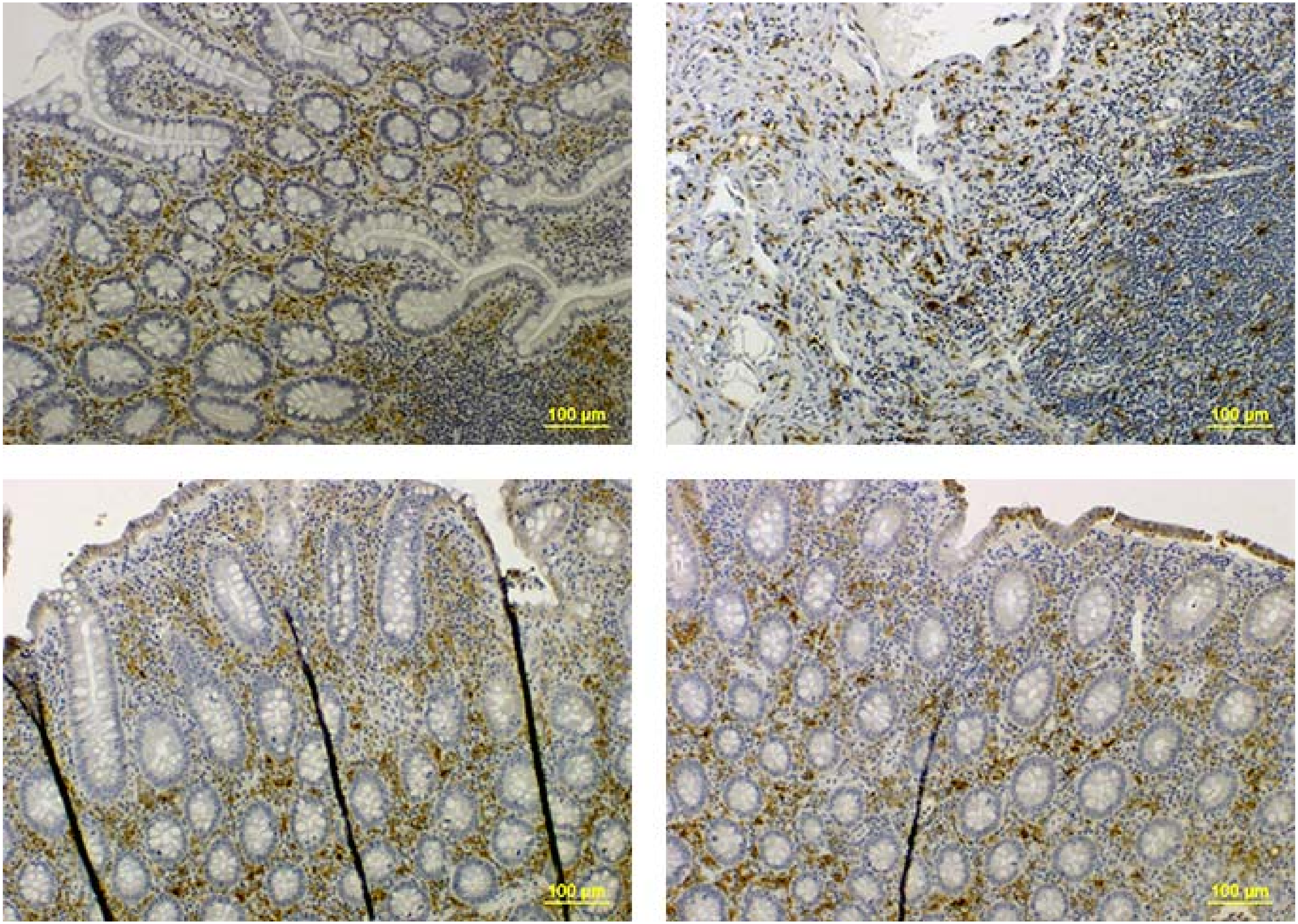

SI Figure 4. Immunohistochemistry staining of thin sections of colon from patients with Ulcerative Colitis. The indicated tissue sections were stained with m909, a human monoclonal antibody to human FR-β, that exhibits no cross-reactivity to other folate receptor isoforms.

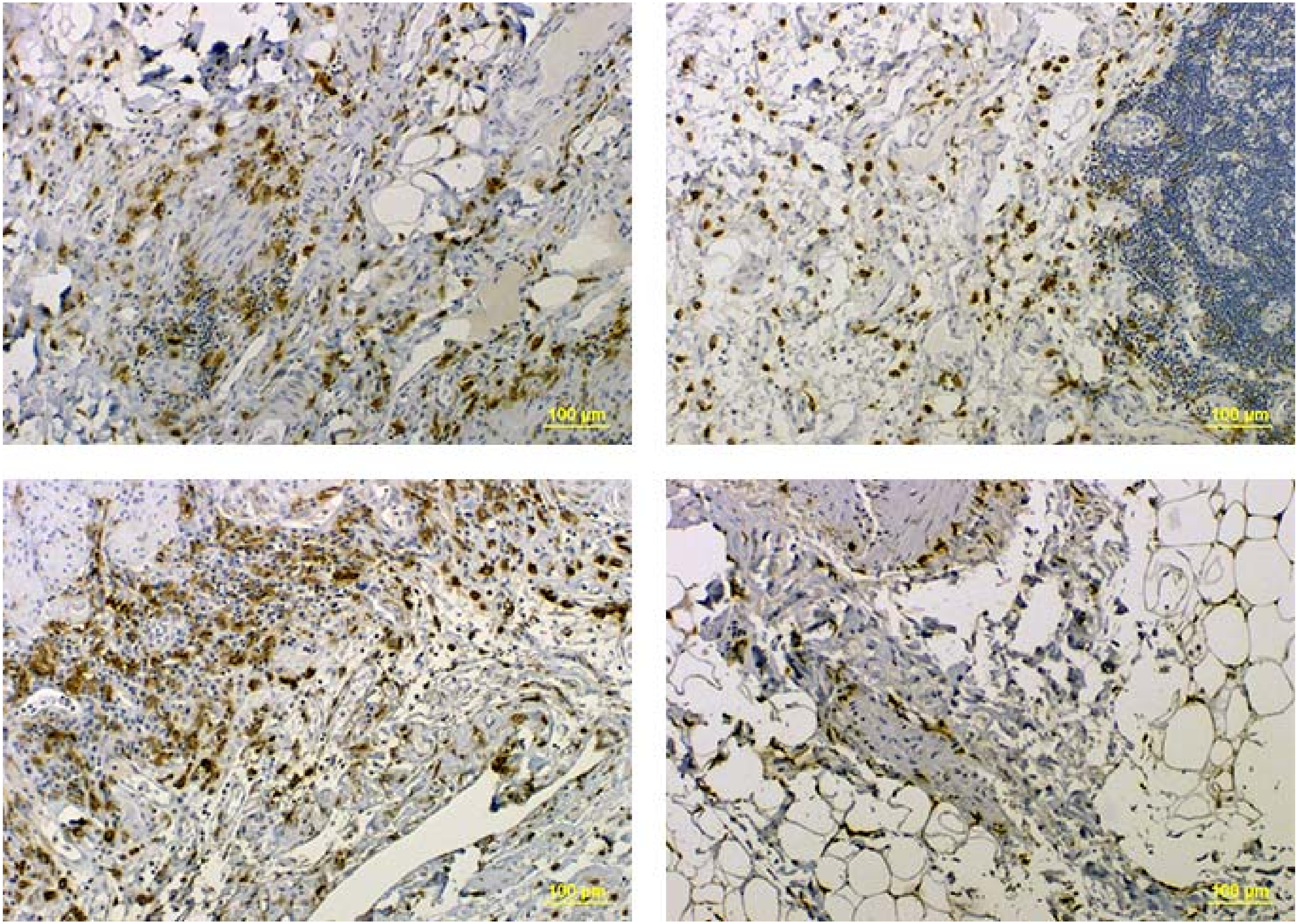

SI Figure 5. Immunohistochemistry staining of thin sections of colon from patients with Ulcerative Colitis. The indicated tissue sections were stained with m909, a human monoclonal antibody to human FR-β, that exhibits no cross-reactivity to other folate receptor isoforms.

Human Inflammatory Disease IHC Staining

Normal Lung

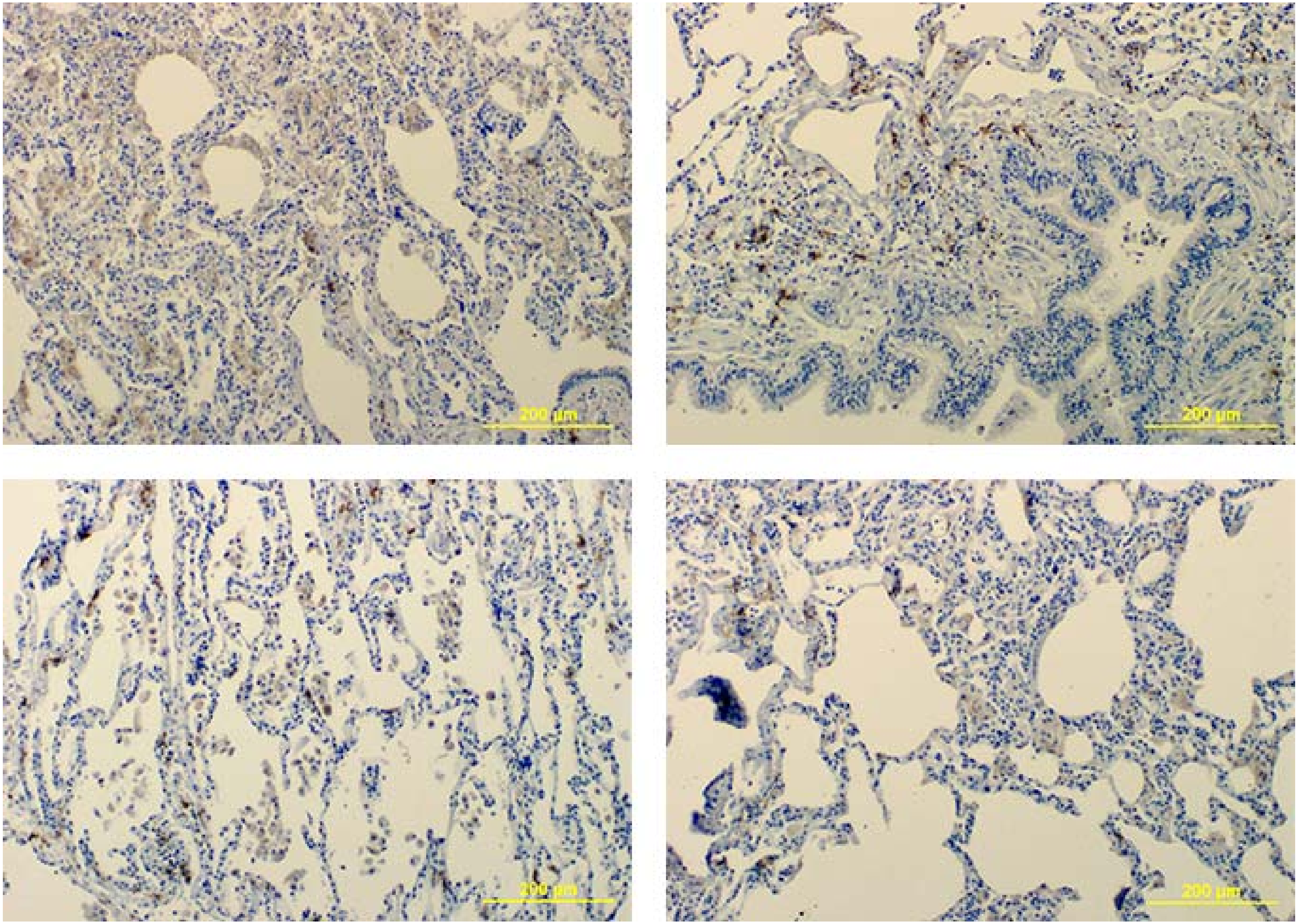

SI Figure 6. Immunohistochemistry staining of thin sections of lung from normal/healthy patients. The indicated tissue sections were stained with m909, a human monoclonal antibody to human FR-β, that exhibits no cross-reactivity to other folate receptor isoforms.

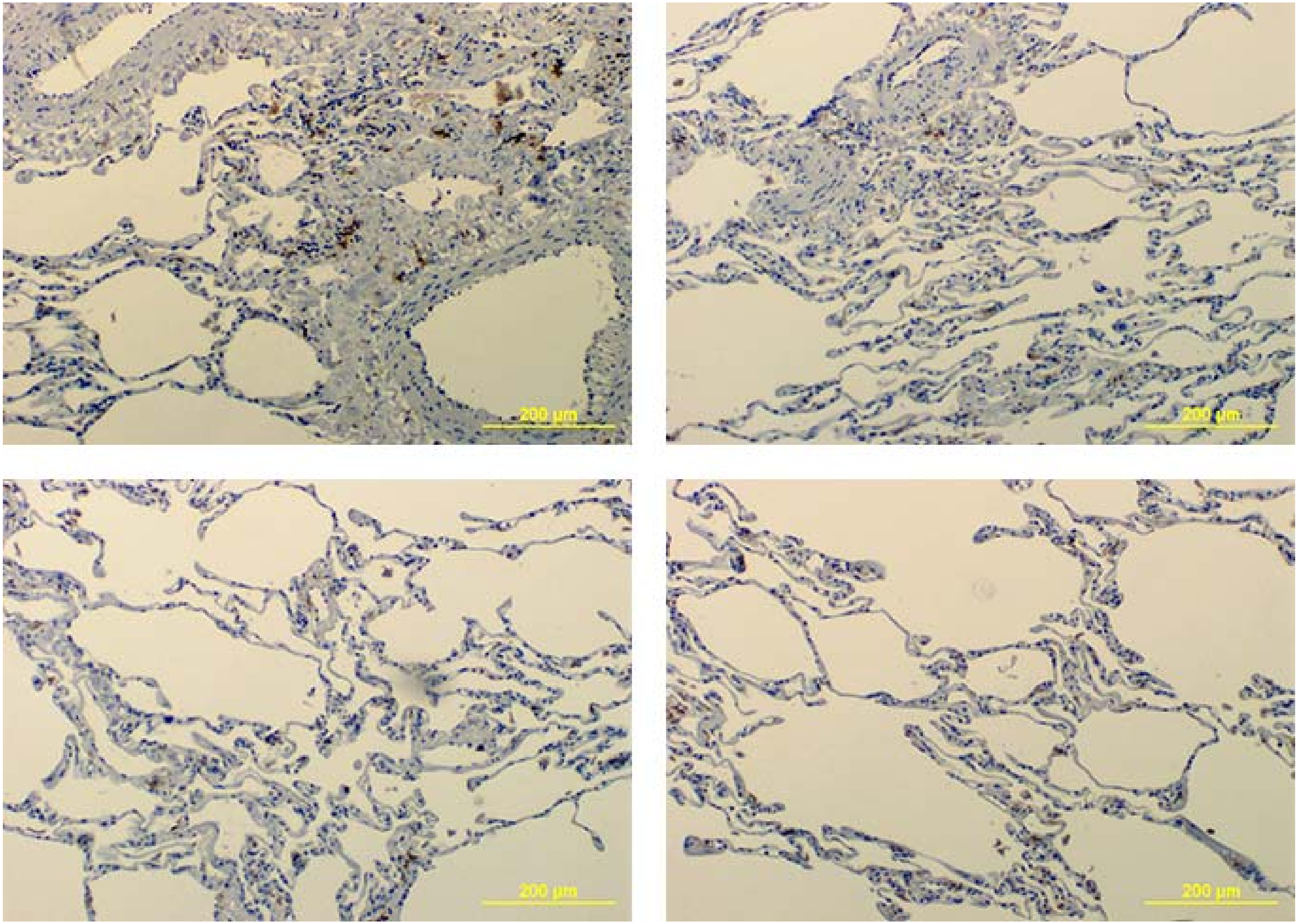

SI Figure 7. Immunohistochemistry staining of thin sections of lung from normal/healthy patients. The indicated tissue sections were stained with m909, a human monoclonal antibody to human FR-β, that exhibits no cross-reactivity to other folate receptor isoforms.

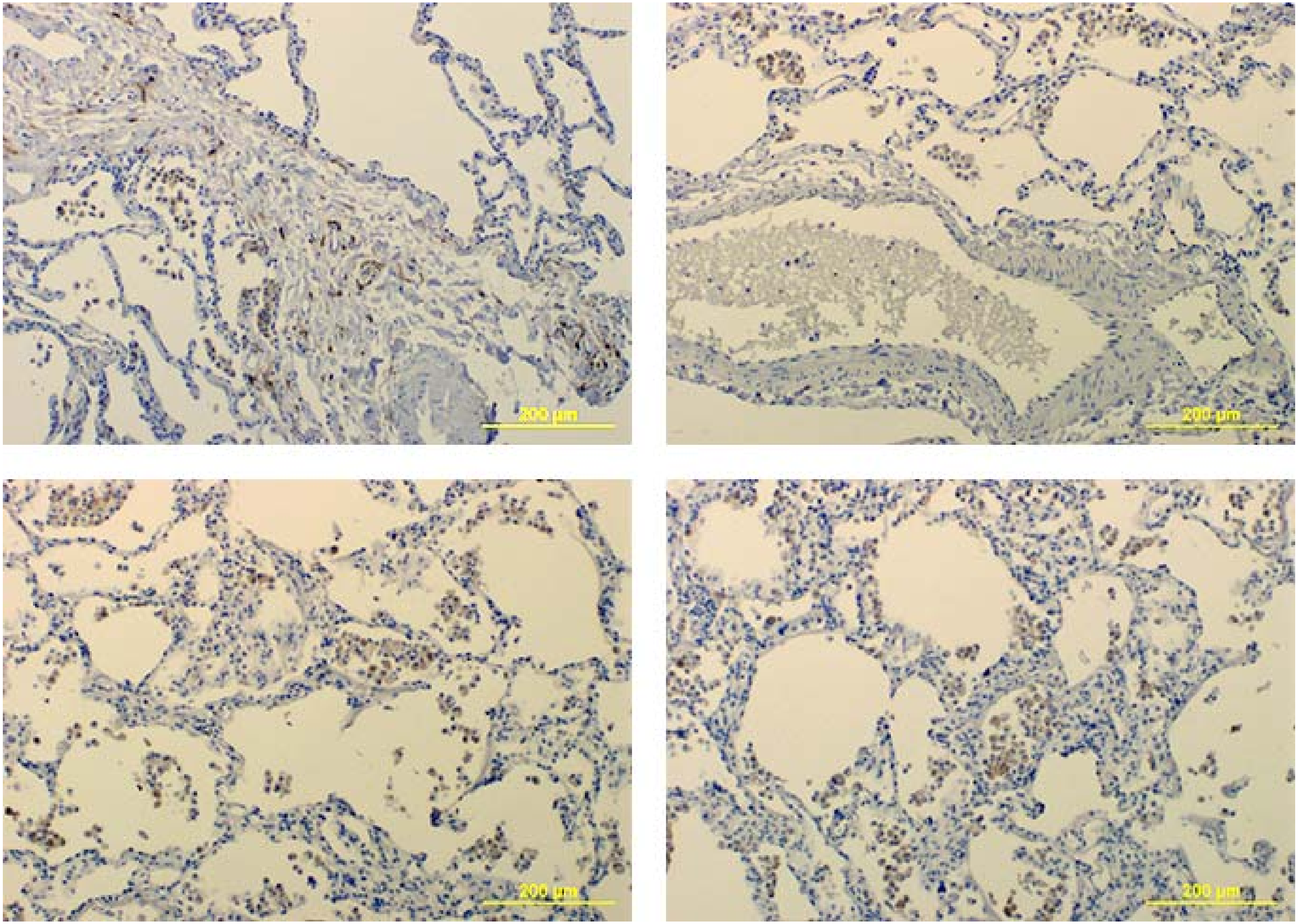

SI Figure 8. Immunohistochemistry staining of thin sections of lung from normal/healthy patients. The indicated tissue sections were stained with m909, a human monoclonal antibody to human FR-β, that exhibits no cross-reactivity to other folate receptor isoforms.

Human Inflammatory Disease IHC Staining

Idiopathic Pulmonary Fibrosis
(IPF)

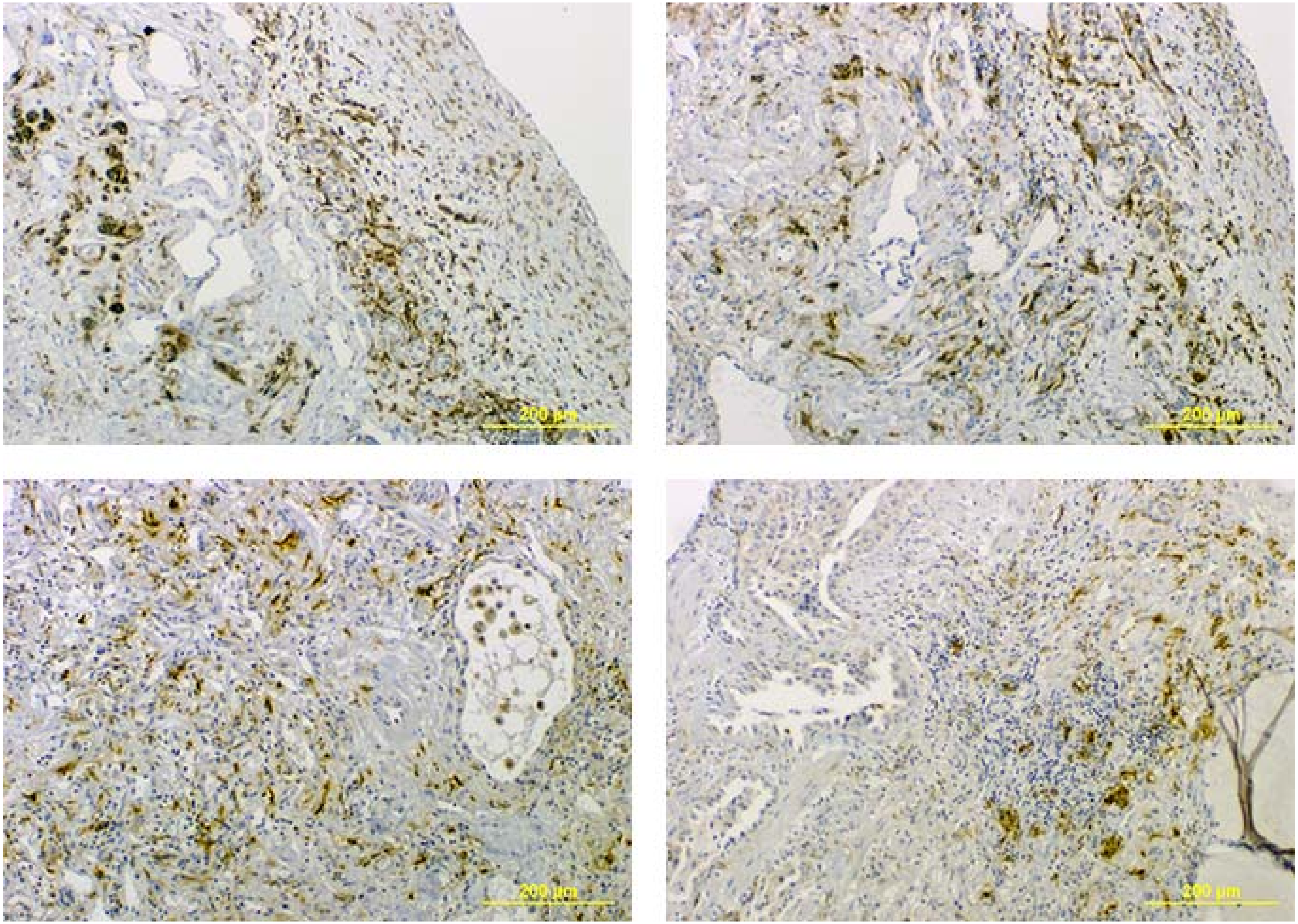

SI Figure 9. Immunohistochemistry staining of thin sections of lung from patients with Idiopathic Pulmonary Fibrosis (IPF). The indicated tissue sections were stained with m909, a human monoclonal antibody to human FR-β, that exhibits no cross-reactivity to other folate receptor isoforms.

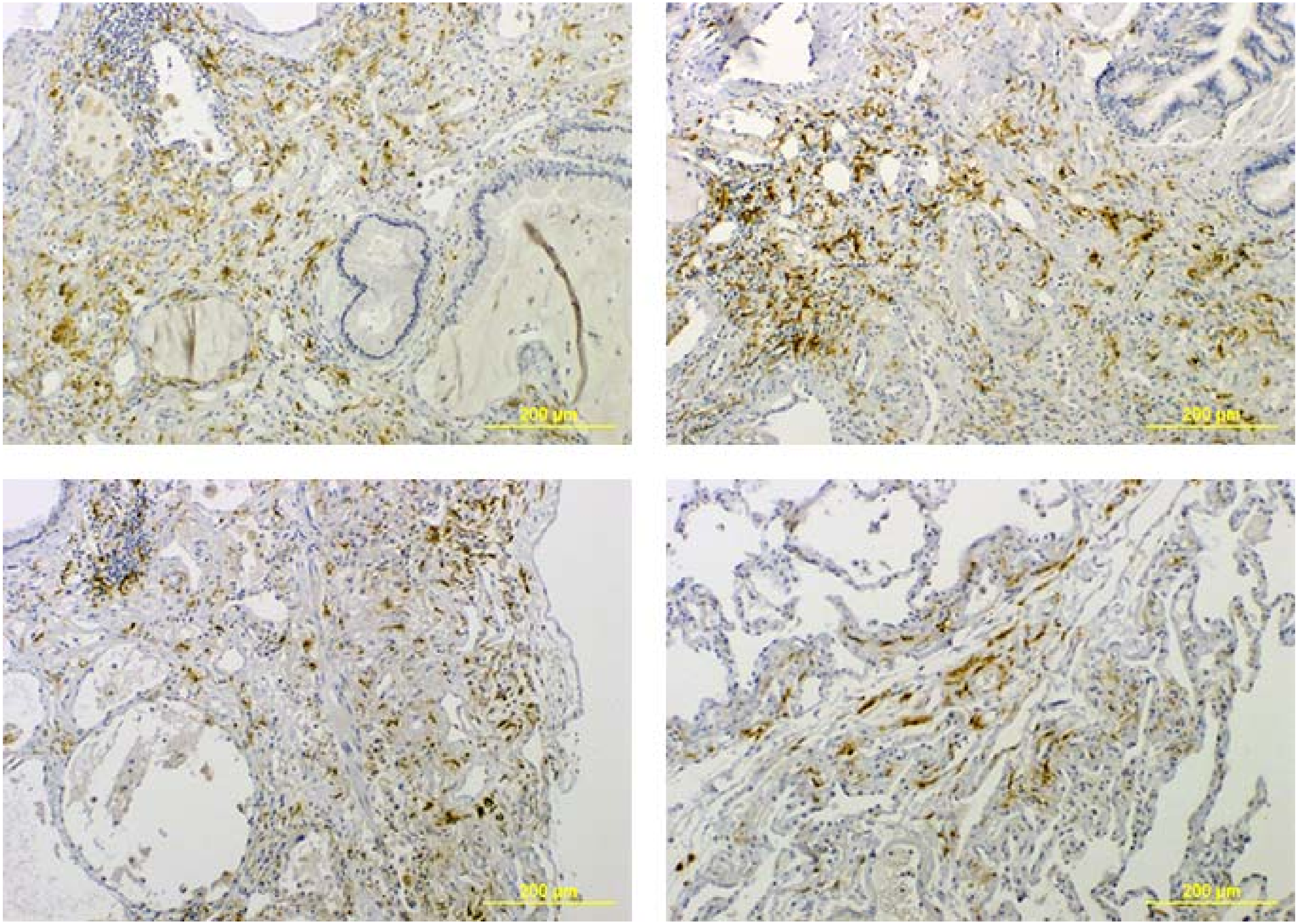

SI Figure 10. Immunohistochemistry staining of thin sections of lung from patients with Idiopathic Pulmonary Fibrosis (IPF). The indicated tissue sections were stained with m909, a human monoclonal antibody to human FR-β, that exhibits no cross-reactivity to other folate receptor isoforms.

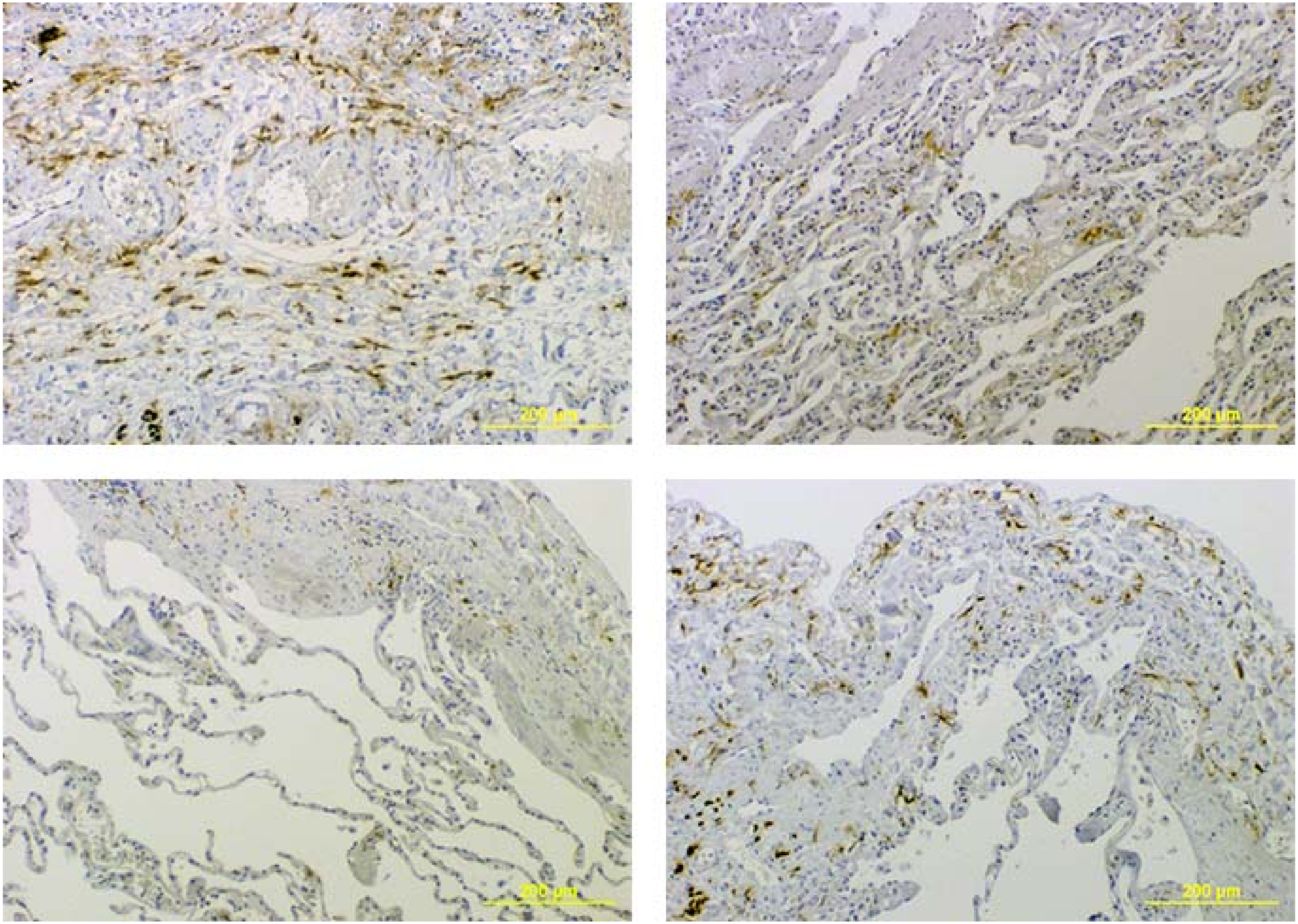

SI Figure 11. Immunohistochemistry staining of thin sections of lung from patients with Idiopathic Pulmonary Fibrosis (IPF). The indicated tissue sections were stained with m909, a human monoclonal antibody to human FR-β, that exhibits no cross-reactivity to other folate receptor isoforms.

Human Inflammatory Disease IHC Staining

Nonspecific Interstitial Pneumonia
(NSIP)

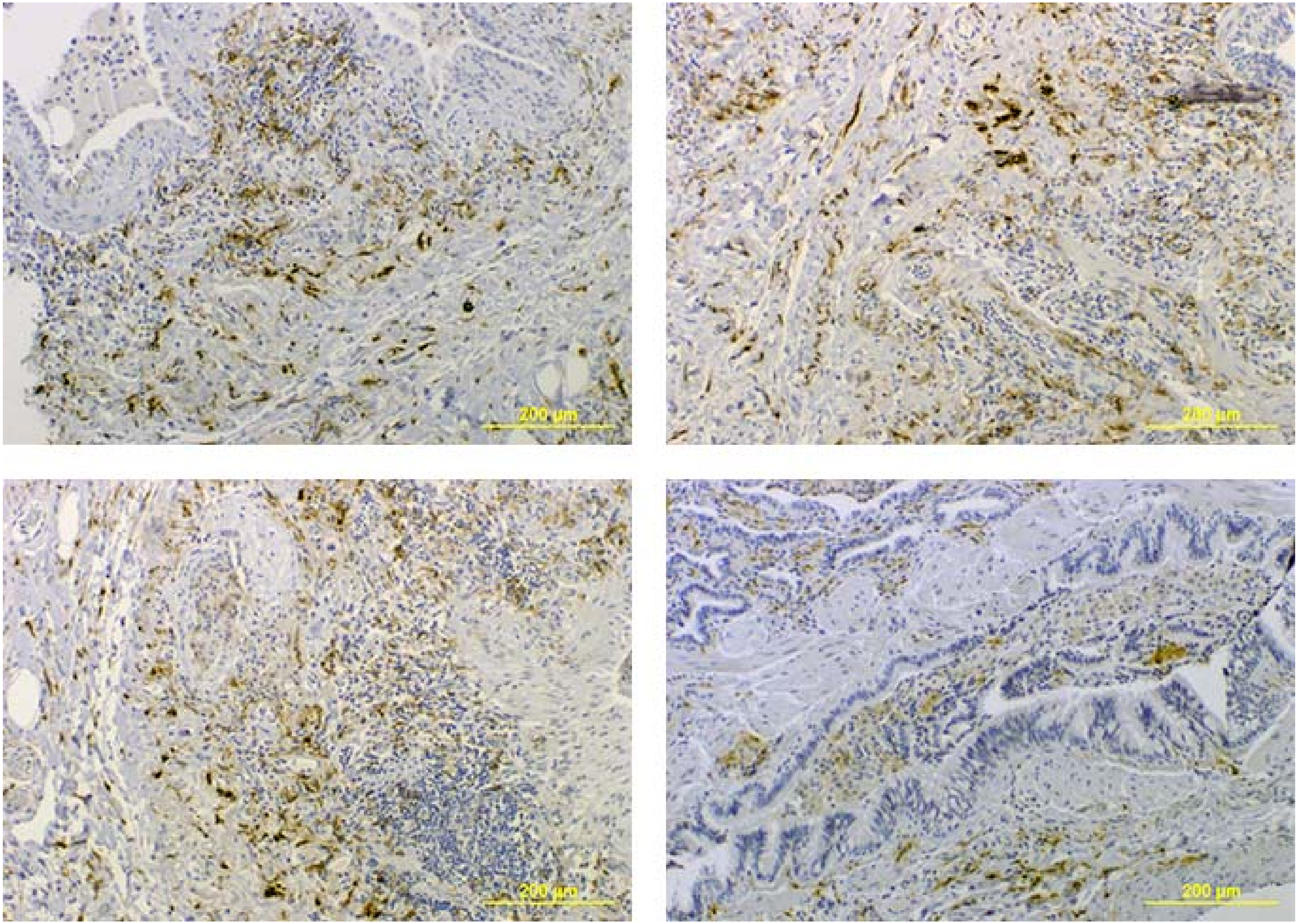

SI Figure 12. Immunohistochemistry staining of thin sections of lung from patients with Nonspecific Interstitial Pneumonia (NSIP). The indicated tissue sections were stained with m909, a human monoclonal antibody to human FR-β, that exhibits no cross-reactivity to other folate receptor isoforms.

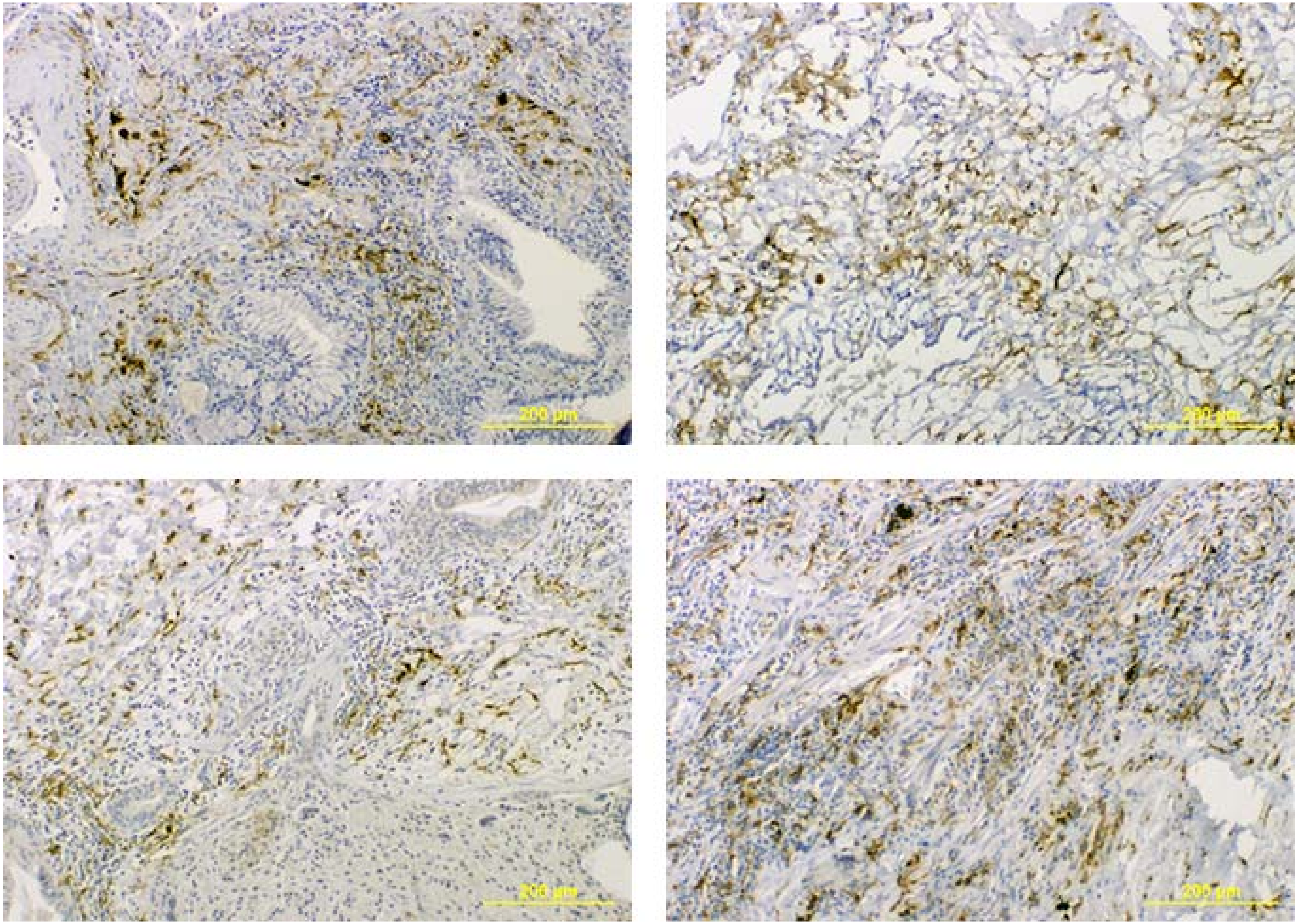

SI Figure 13. Immunohistochemistry staining of thin sections of lung from patients with Nonspecific Interstitial Pneumonia (NSIP). The indicated tissue sections were stained with m909, a human monoclonal antibody to human FR-β, that exhibits no cross-reactivity to other folate receptor isoforms.

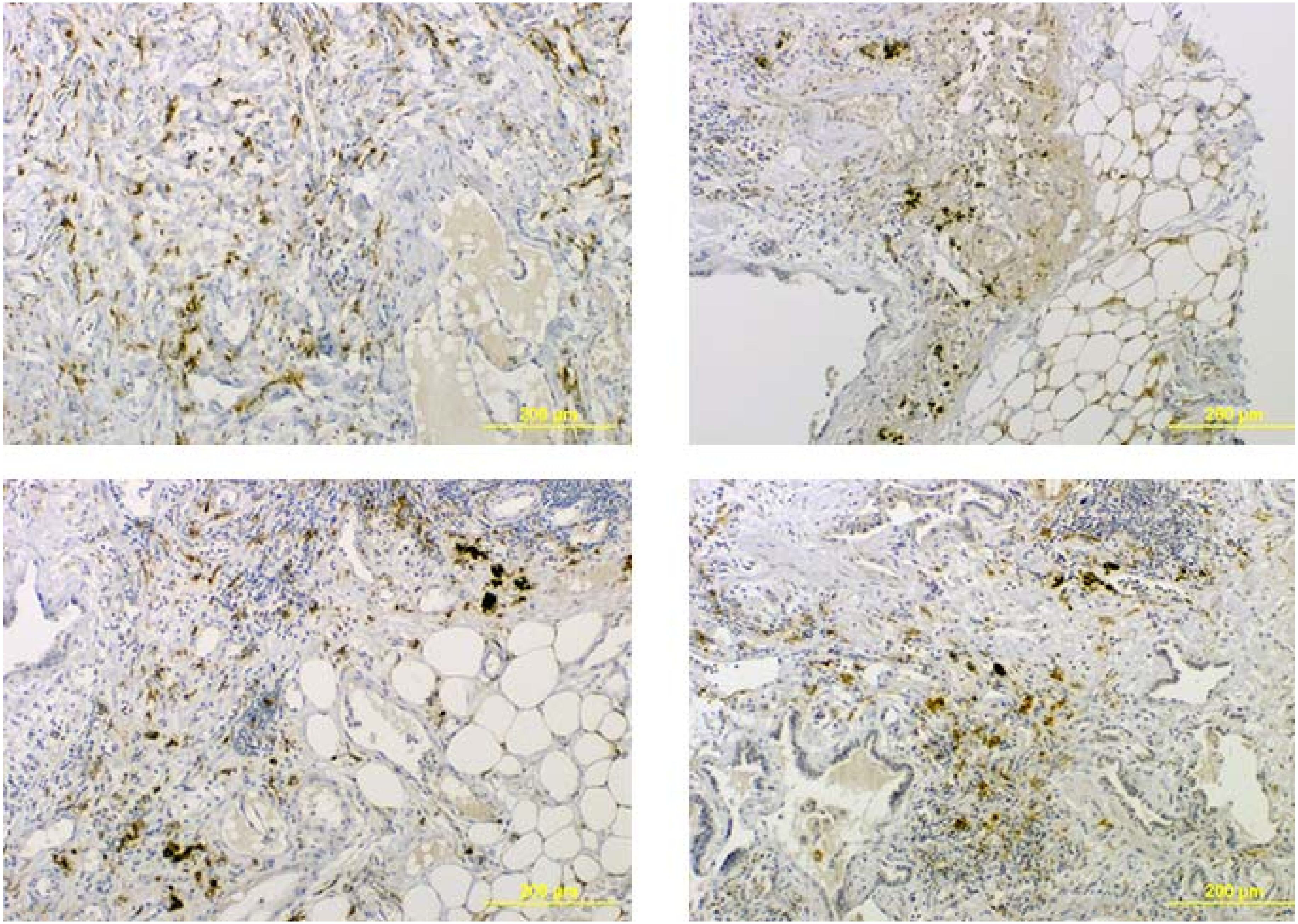

SI Figure 14. Immunohistochemistry staining of thin sections of lung from patients with Nonspecific Interstitial Pneumonia (NSIP). The indicated tissue sections were stained with m909, a human monoclonal antibody to human FR-β, that exhibits no cross-reactivity to other folate receptor isoforms.

Human Inflammatory Disease IHC Staining

Chronic Obstructive Pulmonary Disease (COPD)

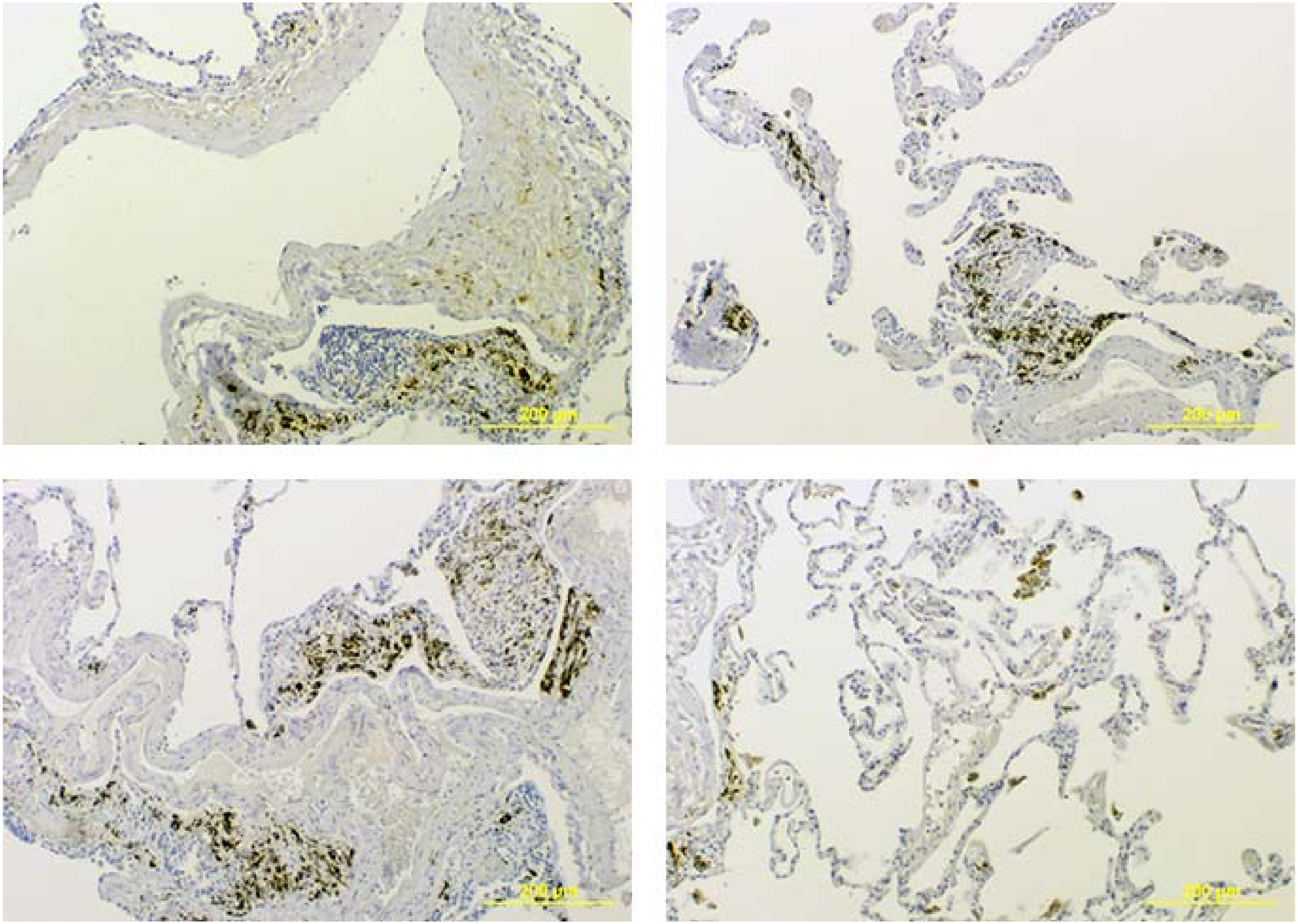

SI Figure 15. Immunohistochemistry staining of thin sections of lung from patients with Chronic Obstructive Pulmonary Disorder (COPD). The indicated tissue sections were stained with m909, a human monoclonal antibody to human FR-β, that exhibits no cross-reactivity to other folate receptor isoforms.

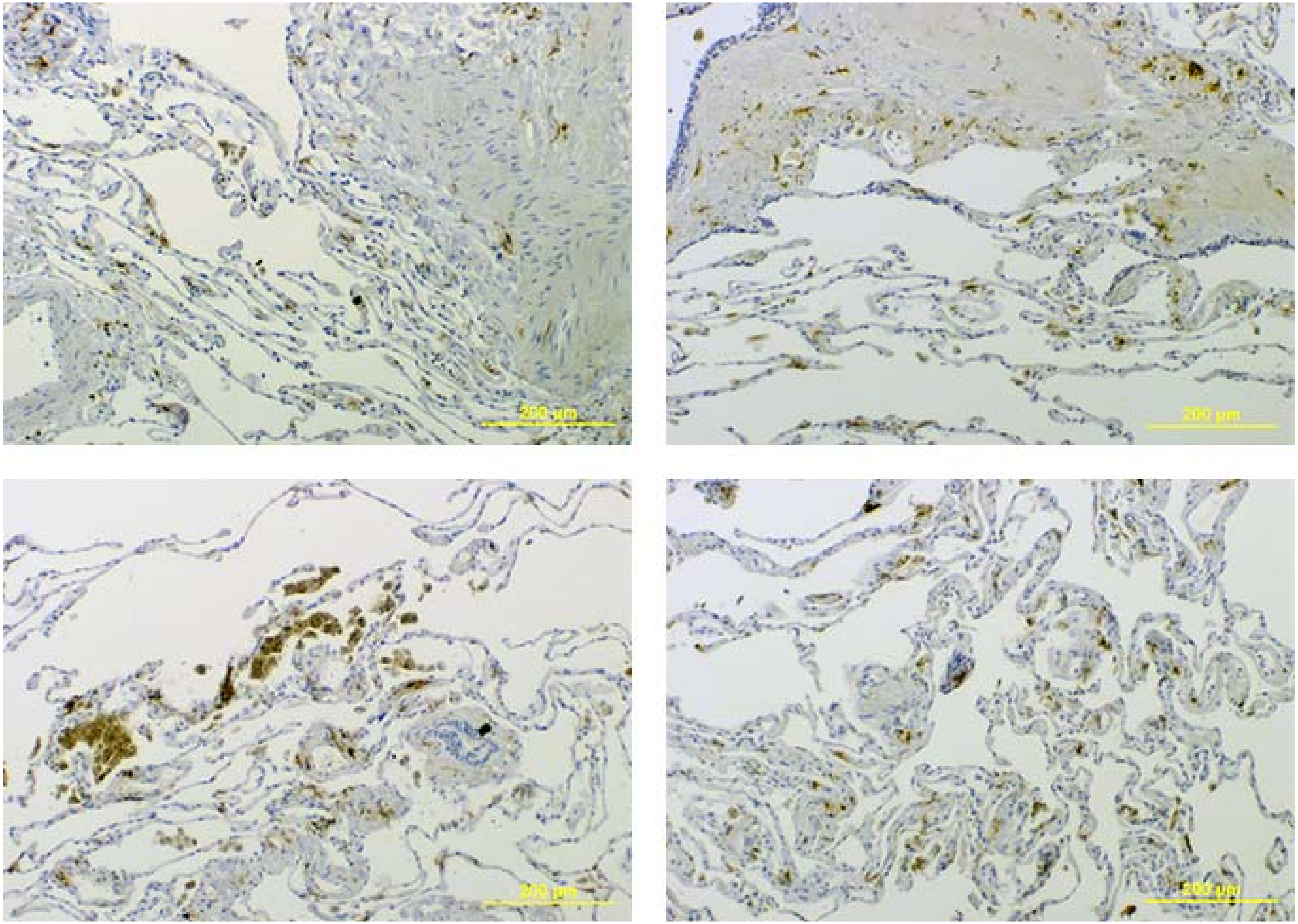

SI Figure 16. Immunohistochemistry staining of thin sections of lung from patients with Chronic Obstructive Pulmonary Disorder (COPD). The indicated tissue sections were stained with m909, a human monoclonal antibody to human FR-β, that exhibits no cross-reactivity to other folate receptor isoforms.

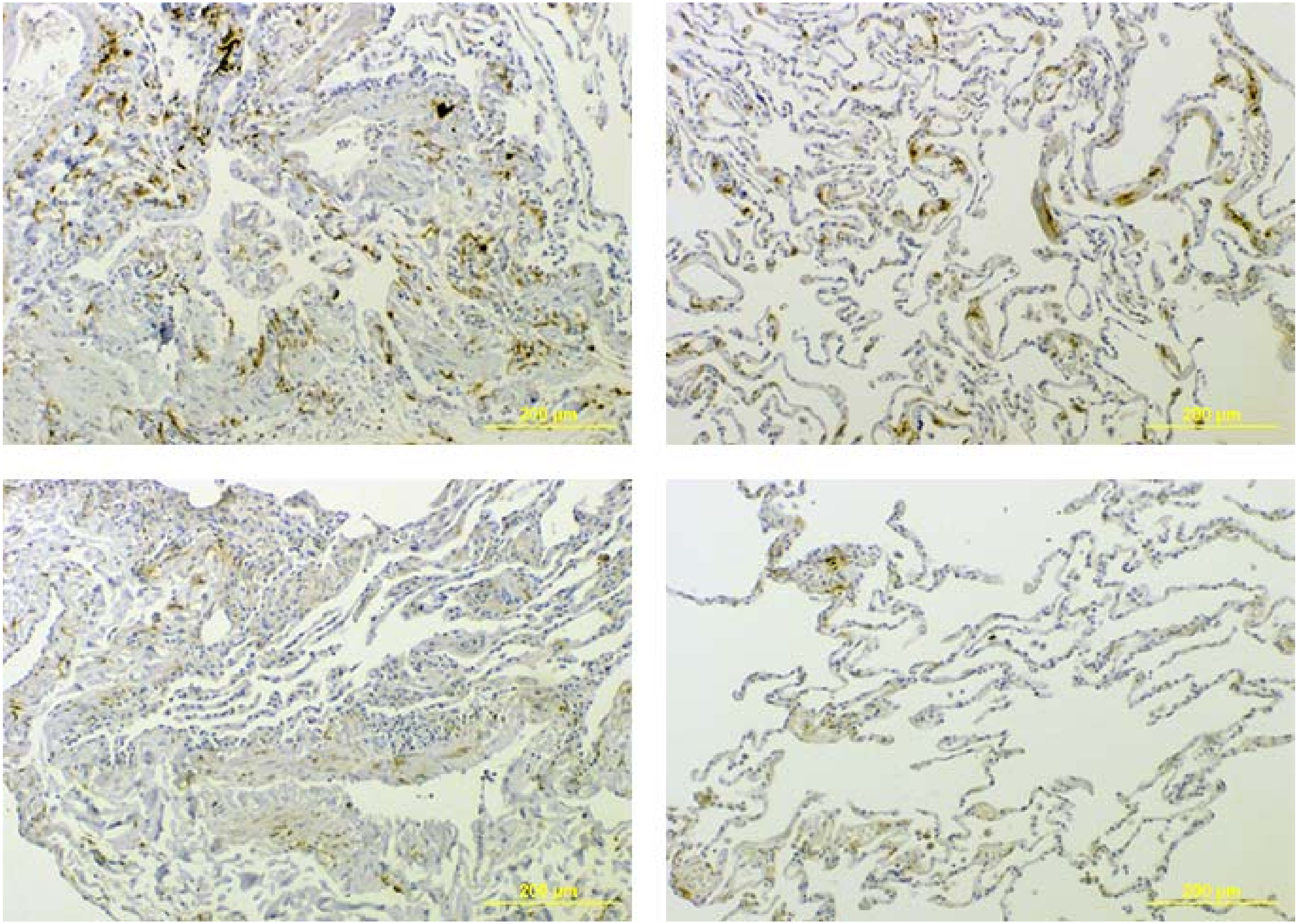

SI Figure 17. Immunohistochemistry staining of thin sections of lung from patients with Chronic Obstructive Pulmonary Disorder (COPD). The indicated tissue sections were stained with m909, a human monoclonal antibody to human FR-β, that exhibits no cross-reactivity to other folate receptor isoforms.

Human Inflammatory Disease IHC Staining

Rheumatoid Arthritis
(RA)

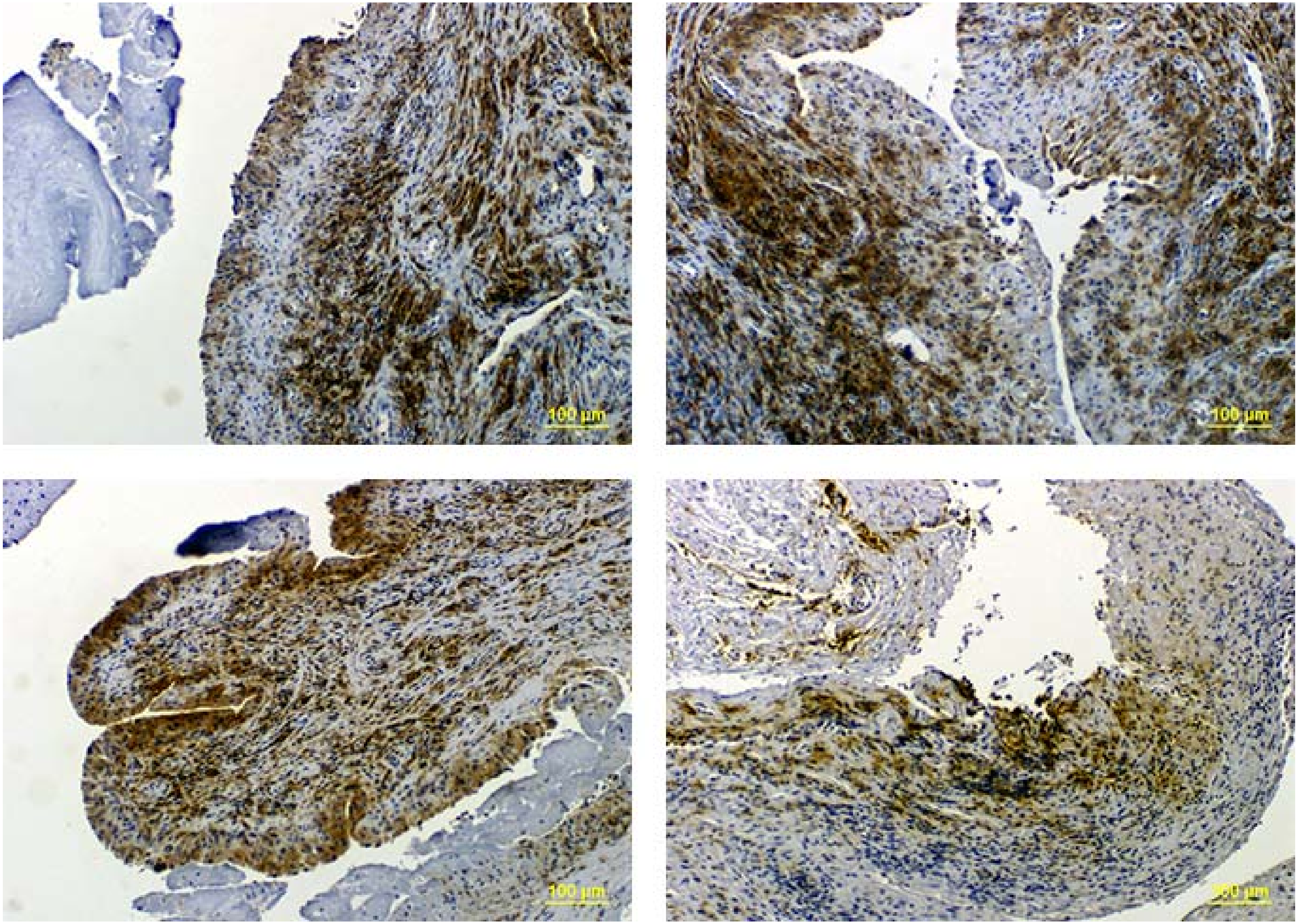

SI Figure 18. Immunohistochemistry staining of thin sections of synovium from patients with Rheumatoid Arthritis (RA). The indicated tissue sections were stained with m909, a human monoclonal antibody to human FR-β, that exhibits no cross-reactivity to other folate receptor isoforms.

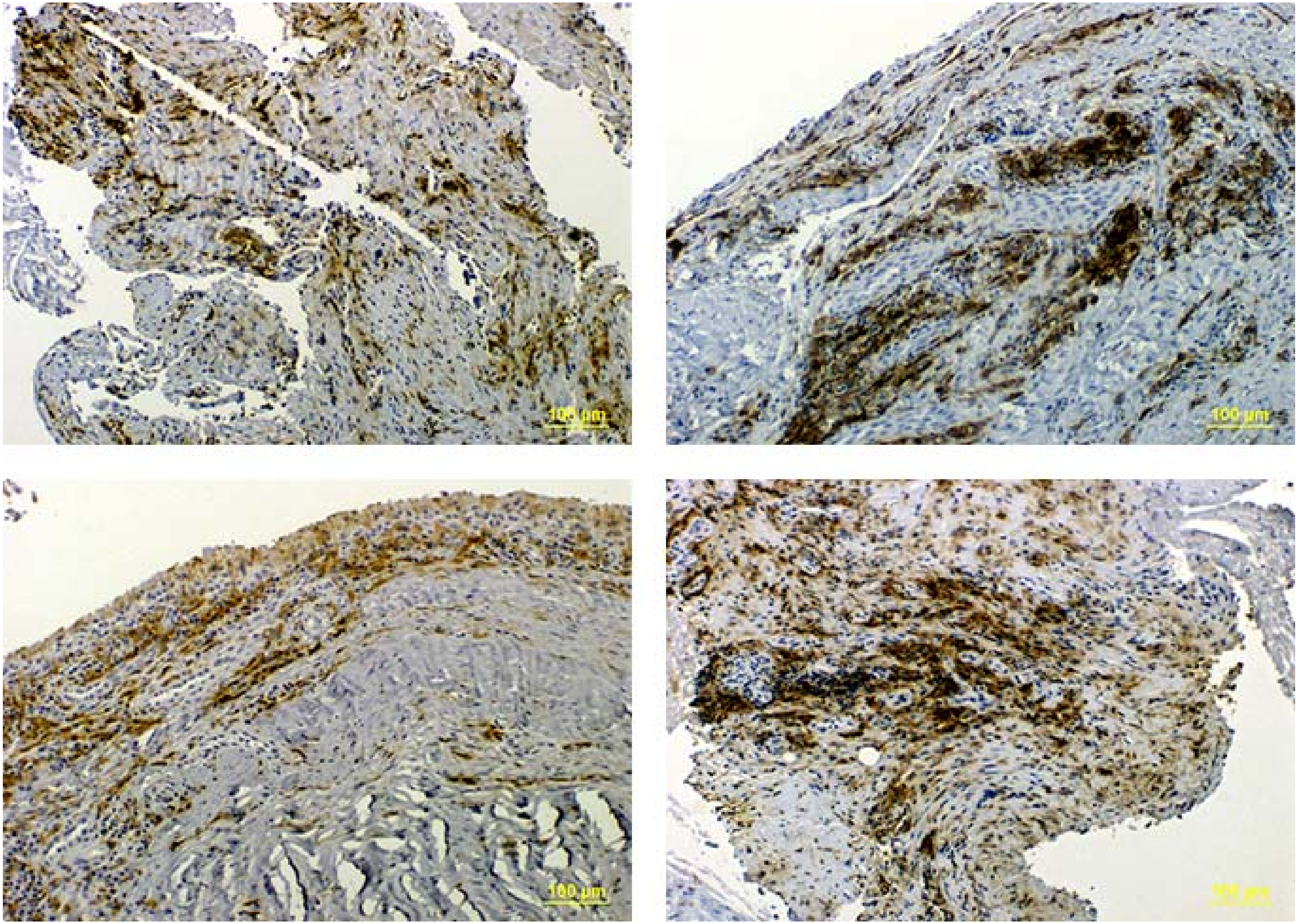

SI Figure 19. Immunohistochemistry staining of thin sections of synovium from patients with Rheumatoid Arthritis (RA). The indicated tissue sections were stained with m909, a human monoclonal antibody to human FR-β, that exhibits no cross-reactivity to other folate receptor isoforms.

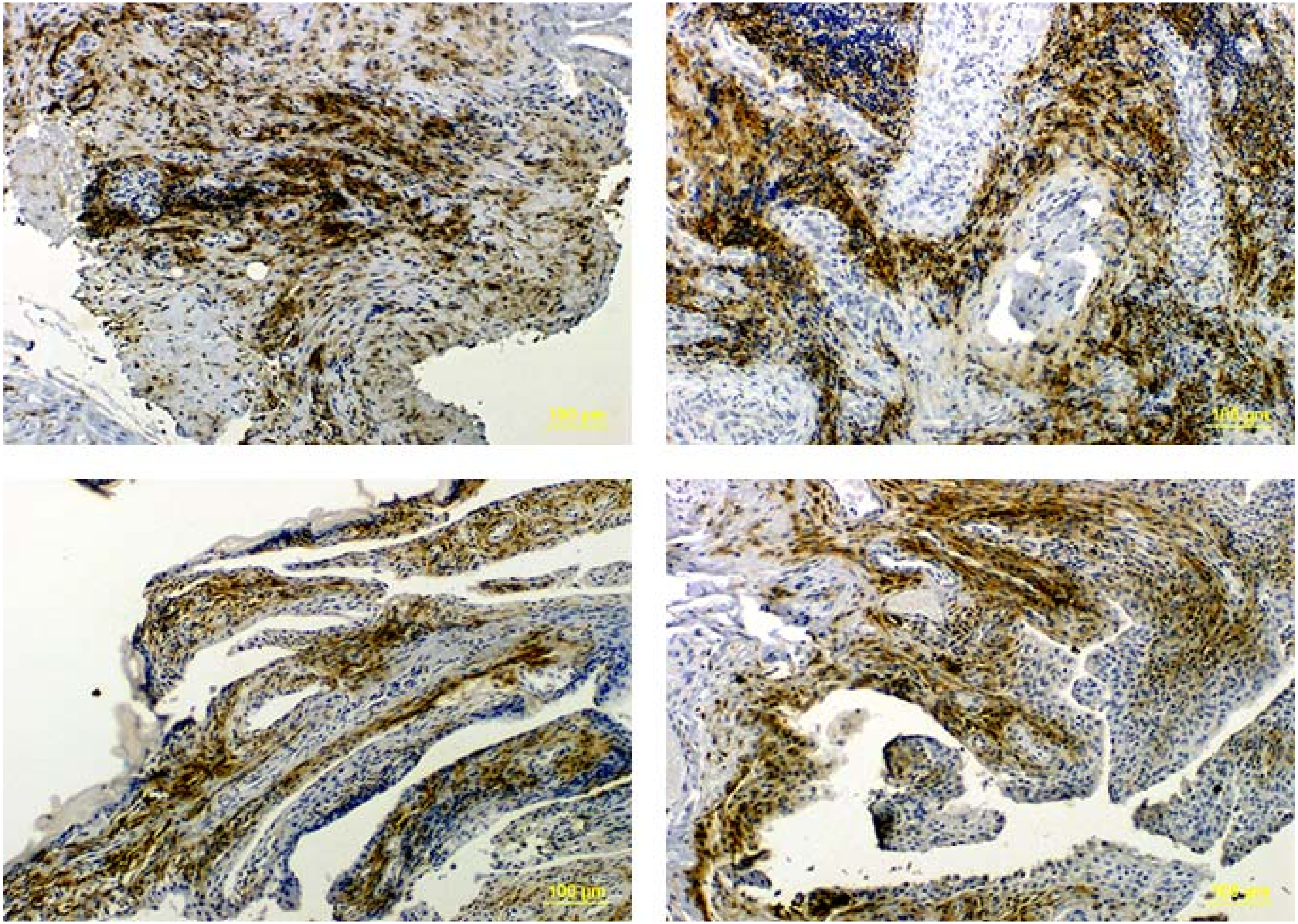

SI Figure 20. Immunohistochemistry staining of thin sections of synovium from patients with Rheumatoid Arthritis (RA). The indicated tissue sections were stained with m909, a human monoclonal antibody to human FR-β, that exhibits no cross-reactivity to other folate receptor isoforms.

Human Inflammatory Disease IHC Staining

Systemic Lupus Erythematosus
(Lupus)

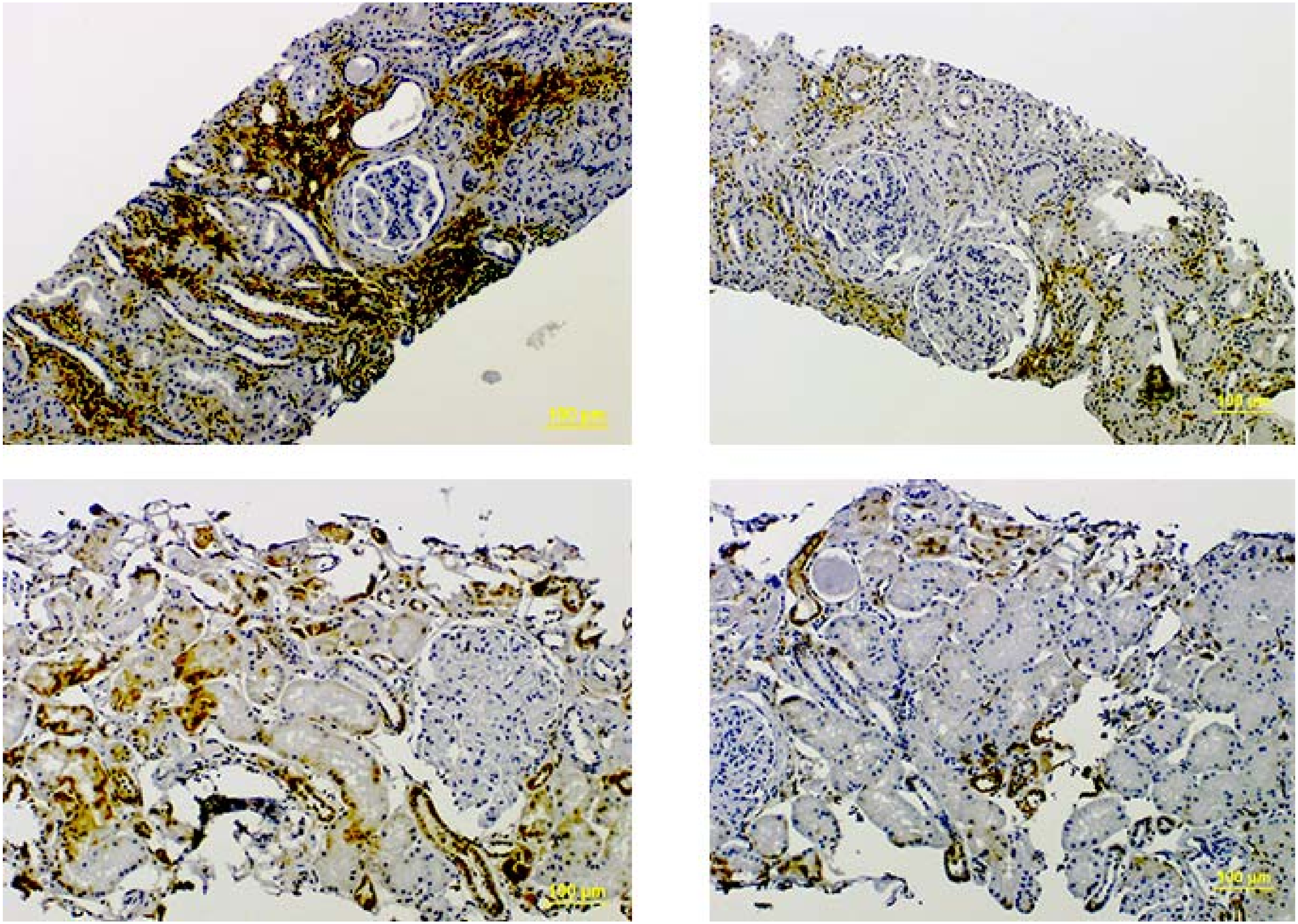

SI Figure 21. Immunohistochemistry staining of thin sections of kidney from patients with Systemic Lupus Erythematosus (Lupus). The indicated tissue sections were stained with m909, a human monoclonal antibody to human FR-β, that exhibits no cross-reactivity to other folate receptor isoforms.

Human Inflammatory Disease IHC Staining

Psoriasis

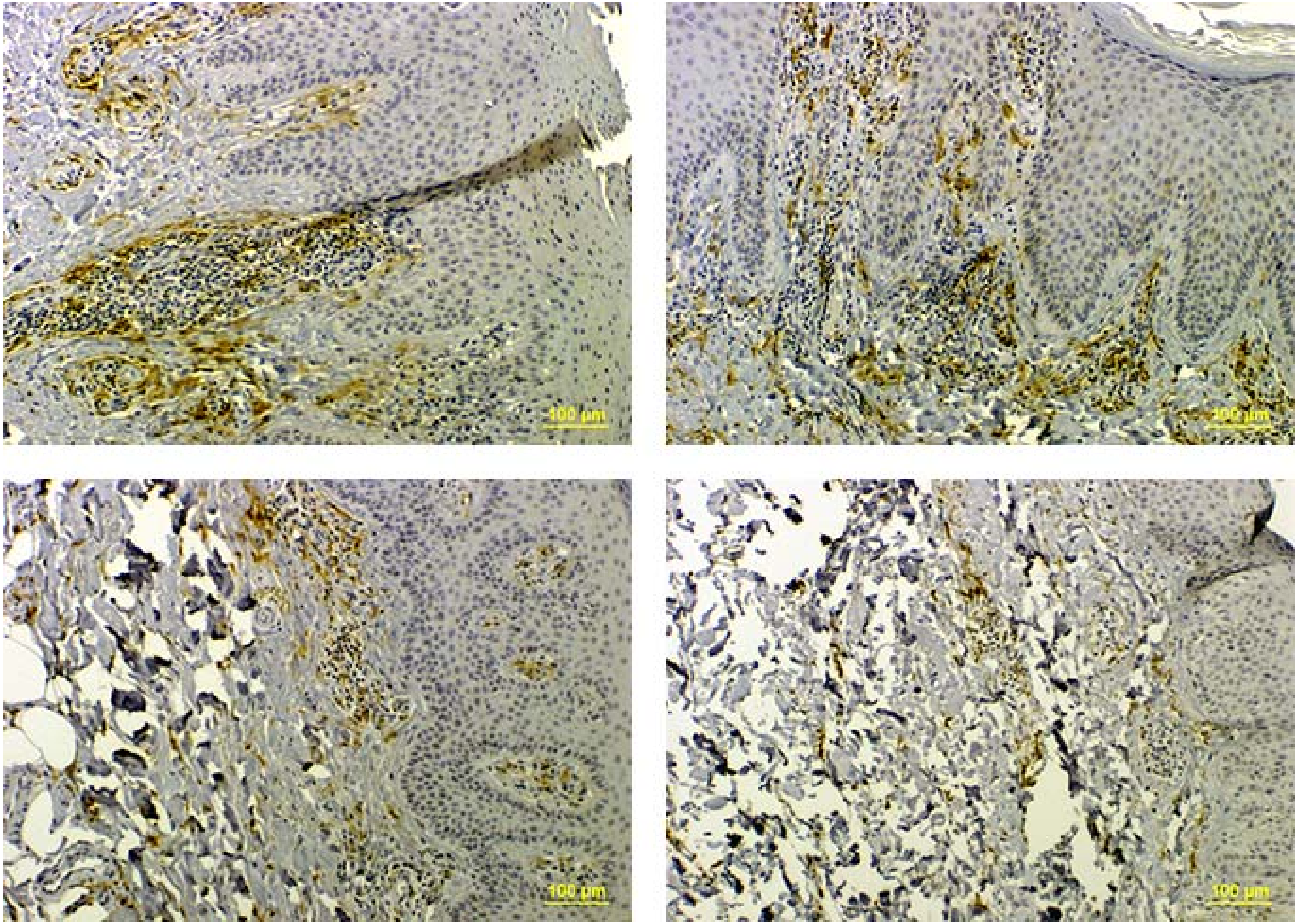

SI Figure 22. Immunohistochemistry staining of thin sections of skin from patients with Psoriasis. The indicated tissue sections were stained with m909, a human monoclonal antibody to human FR-β, that exhibits no cross-reactivity to other folate receptor isoforms.

Human Inflammatory Disease IHC Staining

Scleroderma

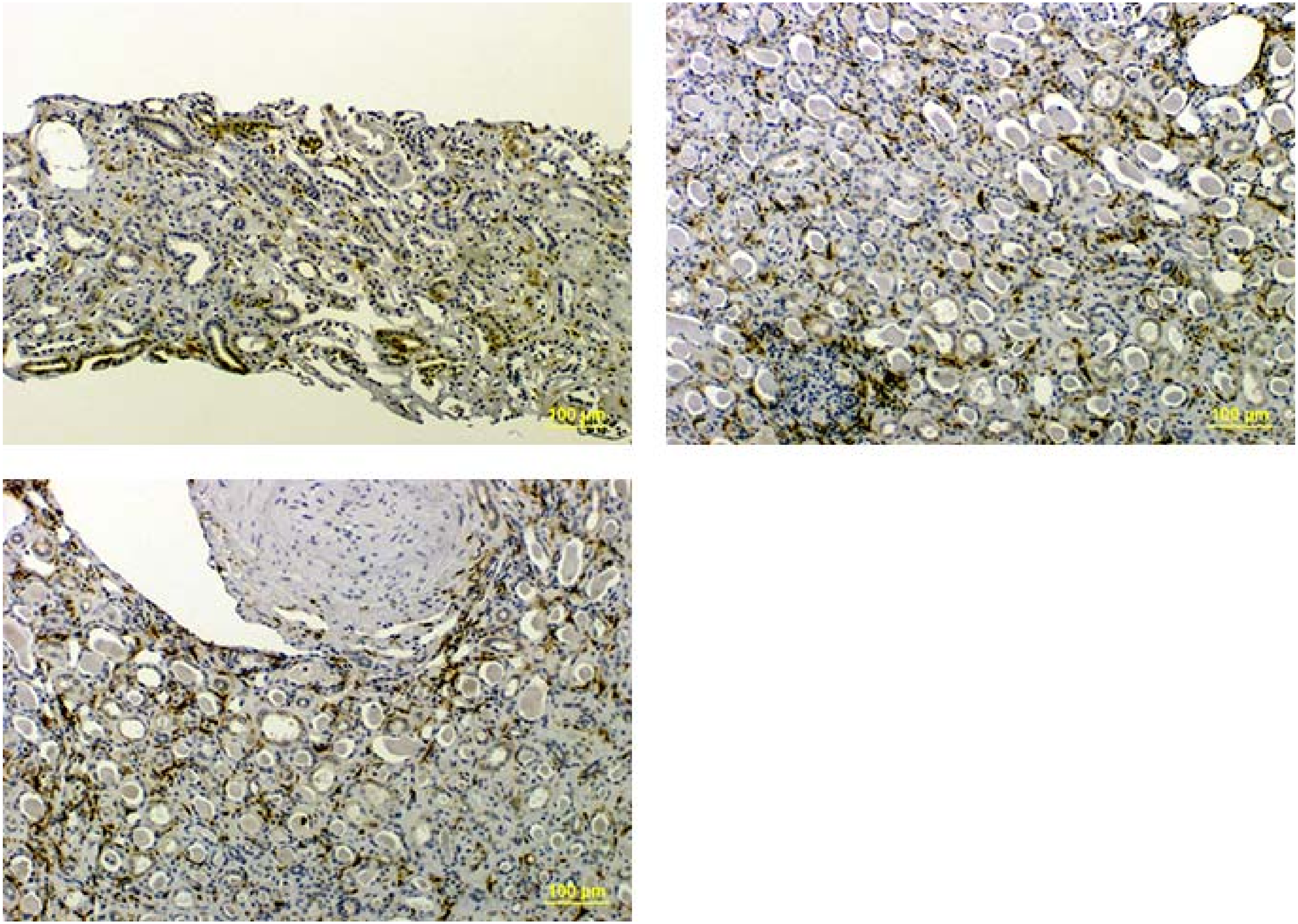

SI Figure 23. Immunohistochemistry staining of thin sections of skin from patients with Scleroderma. The indicated tissue sections were stained with m909, a human monoclonal antibody to human FR-β, that exhibits no cross-reactivity to other folate receptor isoforms.

ADCC

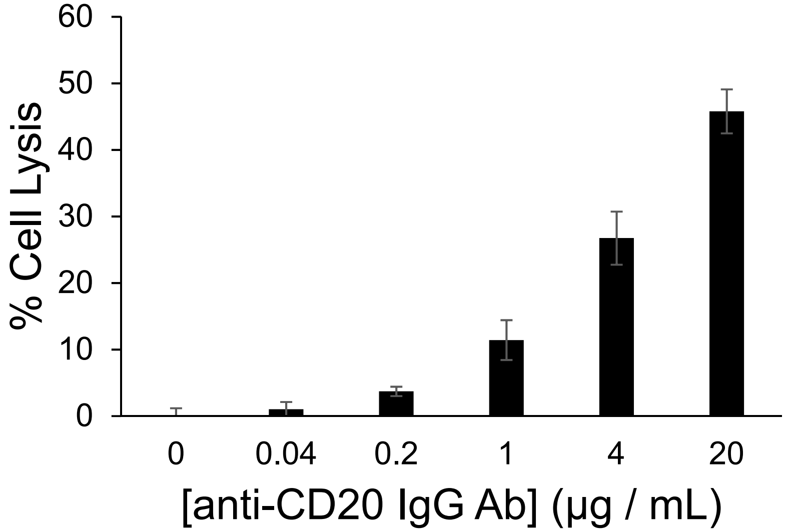

SI Figure 24. Demonstration of ADCC using a well-established positive control. Human PMBCs were isolated from fresh human blood and treated with the indicated concentrations of anti-CD20 antibody. After 24 hours, cell death was determined via an LDH assay.

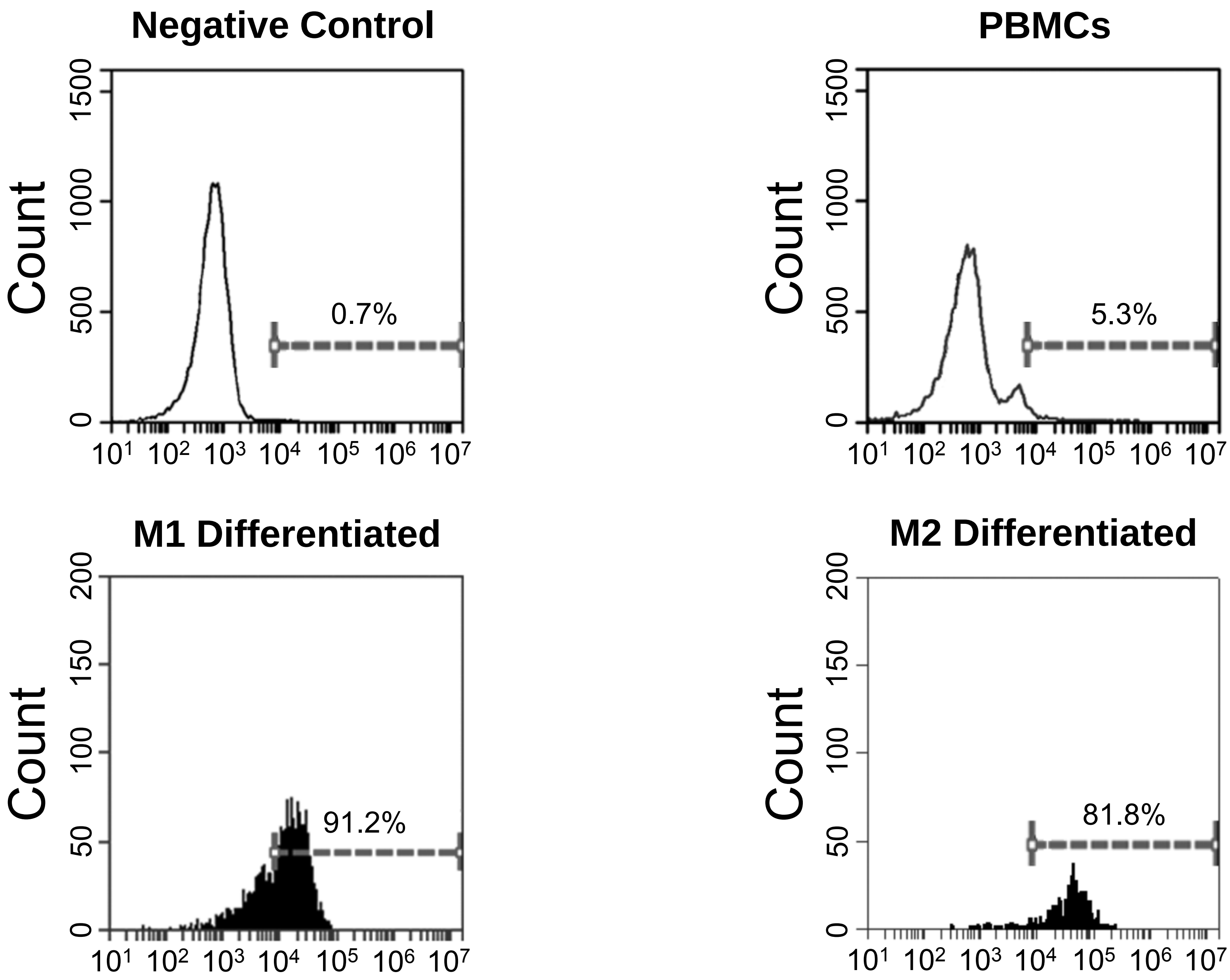

SI Figure 25. Quantitation of the percent of folate receptor beta positive cells in untreated and differentiated human PMBCs. Human PMBCs were isolated from fresh human blood and examined before differentiation or differentiated into M1 or M2 like macrophages as described in Methods. Cells were then incubated with FITC-labeled m909 antibody for 0.5 hours. After washing, bound fluorescence was determined via flow cytometry.

1-way ANOVA - m909 ADCC

	[m909] (μg/mL)																	
	0.0002			0.002			0.02			0.2			2			20		
SS (Between | Within | Total)	713	15	728	1893	652	2545	1805	176	1981	2463	2564	5027	3417	43	3460	5722	3627	9350
df (Between | Within | Total)	2	10	12	2	10	12	2	10	12	2	10	12	2	10	12	2	10	12
MS (Between | Within)	356.5	3.7		946.7	81.5		902.4	44.0		1231.7	256.4		1708.4	10.9		2861.2	362.7	
F	97.385			11.617			20.536			4.803			157.447			7.888		
p-value	0.0004			0.0043			0.0079			0.0345			0.0002			0.0088		

	post-hoc Tukey Test (p-value)					
No Macs vs. M1 Macs	0.0010	0.0078	0.0281	0.0494	0.0010	0.0121
No Macs vs. M2 Macs	0.0026	0.0059	0.0080	0.0426	0.0010	0.0131
M1 Macs vs. M2 Macs	0.0172	0.9000	0.3188	0.9000	0.6706	0.9000

SI Figure 26. Statistical analysis of the differences in m909-mediated ADCC of M1 and M2 macrophages (Macs) (from **Fig. 2C**). Human PMBCs were isolated from blood and differentiated into M1 or M2 macrophages prior to isolation and incubation with undifferentiated PBMCs in the presence of various concentrations of m909. After 24 hours, cell death was determined via the LDH assay. A 1-way ANOVA was used to determine if there were any significant differences in cell killing between undifferentiated PBMCs, M1- and M2-like differentiated macrophages. A p-value < 0.05 was considered significant and highlighted in yellow.

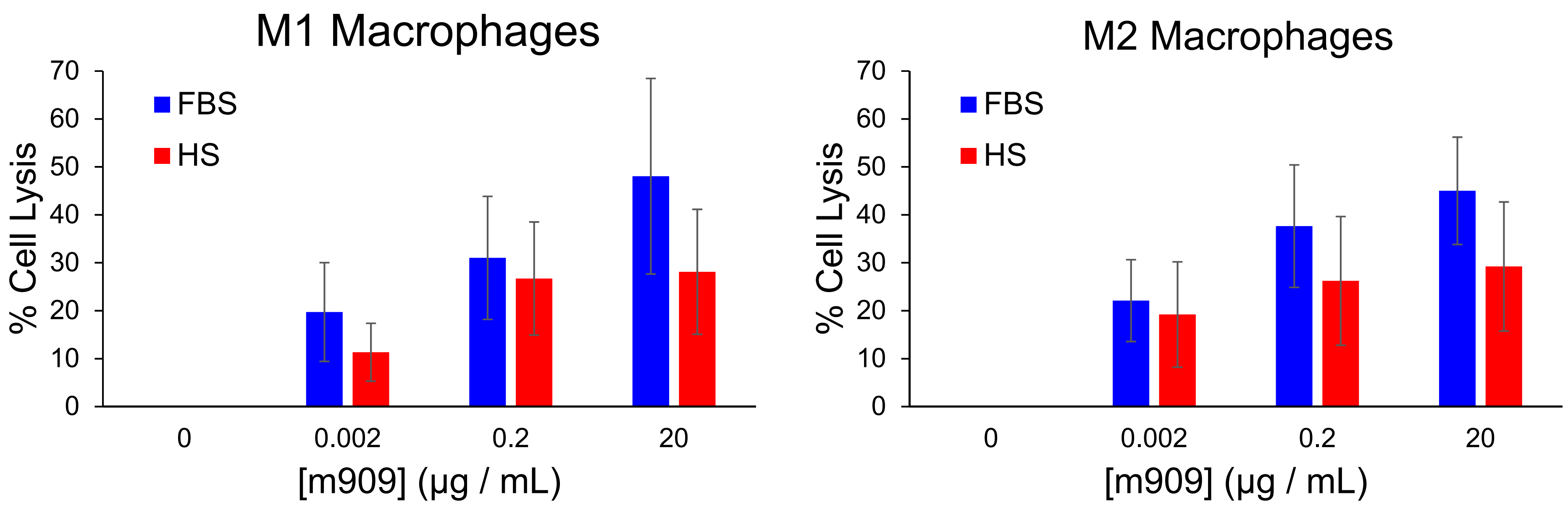

M1 Macrophages - FBS

	m909 (µg / mL)			
	0	0.002	0.2	20
p-value	-	0.5210	0.8172	0.4565

M2 Macrophages - FBS

	m909 (µg / mL)			
	0	0.002	0.2	20
p-value	-	0.8447	0.5711	0.4181

SI Figure 27. Effect of human serum on m909-mediated ADCC of FR-β-positive M1 and M2 macrophages. Human PMBCs were isolated from blood and differentiated into M1 and M2 macrophages as described in Methods. Macrophages were isolated and added to undifferentiated PBMCs in the presence of various concentrations of m909. After 24 hours, cell death was determined via the LDH assay. A t-test was used to determine if there were any significant differences in cell killing between HS and FBS. Assuming a p-value < 0.05 indicates a significant difference, human serum had no significant effect on m909-mediated ADCC.

Thioglycollate Model

t-test (p-value)

	Time (hours)			
	8	24	48	96
Macrophages	0.0026	0.0015	0.0007	0.0002
Neutrophil	0.0441	0.0041	0.0362	0.0073
Dendritic Cells	0.0670	0.0652	0.2691	0.0094
NK cells	0.0992	0.0654	0.0919	0.0002
T-cells	0.0695	0.3802	0.1780	0.1797
B-cells	0.6073	0.1781	0.2980	0.0554

SI Figure 28. Statistical analysis of the differences in immune cell accumulation in peritoneal fluid after thioglycollate injection (from **Fig. 3A**). Mice were injected intraperitoneally with thioglycollate (1 mL of a 3% solution) and sacrificed at various times after injection. IP fluid was removed, stained with various antibodies against cell-type markers and analyzed via flow cytometry. A t-test was used to determine if there were any significant differences in immune cell numbers between healthy and thioglycollate treated animals. A p-value < 0.05 was considered significant and highlighted in yellow.

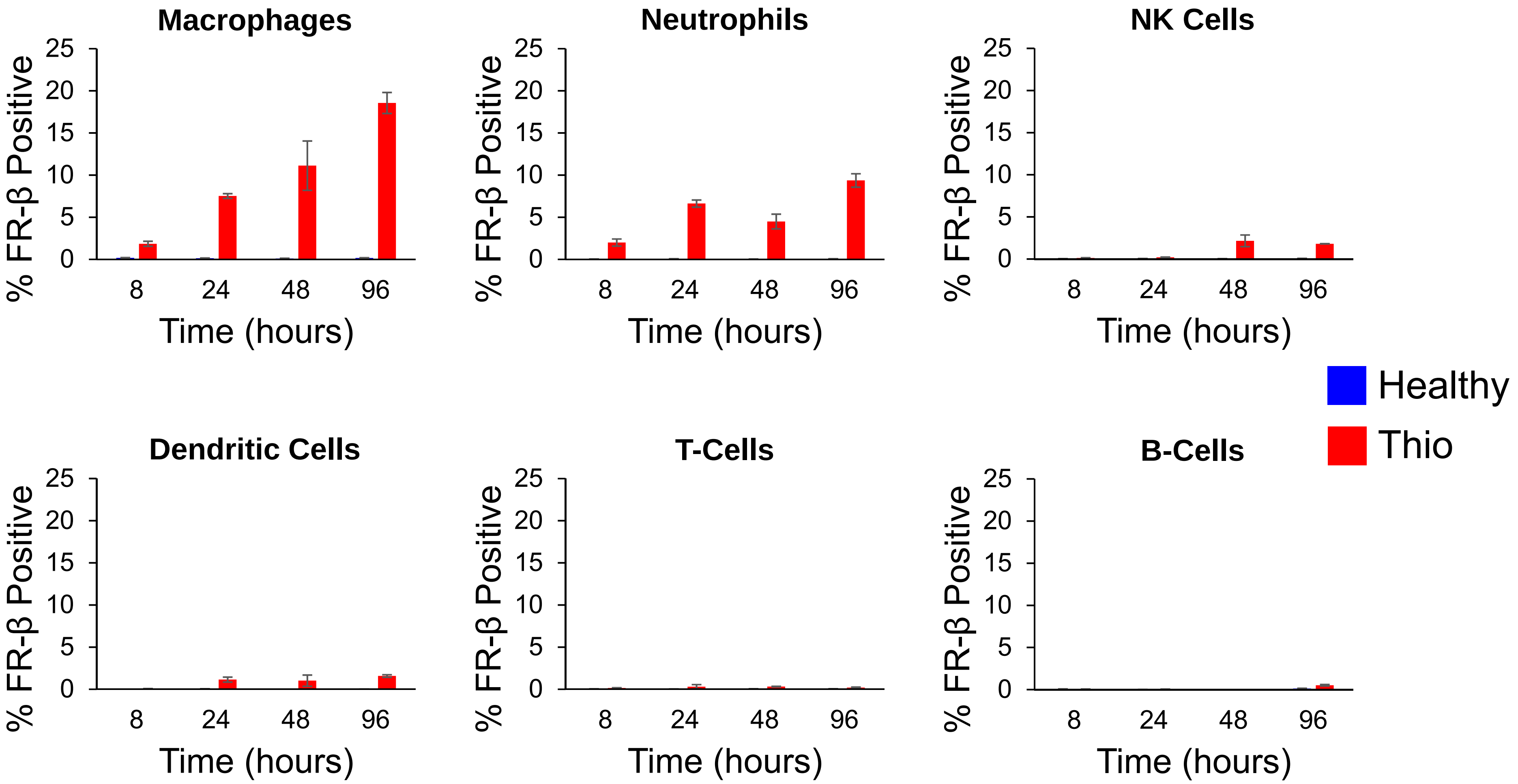

SI Figure 29. Quantitation of folate receptor beta positive (F3 positive) immune cell populations in the peritoneal fluid of mice at various times after thioglycollate injection (from **Fig. 3B**). Mice were injected IP with thioglycollate (1 mL of a 3% solution) and sacrificed at various times after the injection. IP fluid was removed, stained with various antibodies against both cell-type markers and mouse FR-β prior to analysis by flow cytometry. The percentage of each cell type that was FR-β positive is plotted versus time post-thioglycollate injection. Error bars represent standard error.

t-test (p-value)

	Time (hours)			
	8	24	48	96
Macrophages	0.0052	0.0001	0.0151	0.0003
Neutrophil	0.0086	0.0002	0.0063	0.0006
Dendritic Cells	0.0006	0.0155	0.1351	0.0008
NK cells	0.0294	0.0179	0.0260	0.0001
T-cells	0.0183	0.2309	0.0026	0.0731
B-cells	0.5263	0.0755	0.1880	0.1401

SI Figure 30. Statistical analysis of differences in FR-β positive (F3 positive) immune cells from the peritoneal fluid of mice after thioglycollate injection (from **Fig. 3B** and **SI Fig. 29**). Mice were injected IP with thioglycollate (1 mL of a 3% solution) and sacrificed at various times after the injection. IP fluid was removed, stained with various antibodies against both cell-type markers and mouse FR-β prior to analysis by flow cytometry. The percentage of each cell type that was FR-β positive is plotted versus time post-thioglycollate injection. A p-value < 0.05 was considered significant and highlighted in yellow.

	t-test (p-value)
Macrophages	0.0001
Neutrophil	0.0002
Dendritic Cells	0.9201
NK cells	0.0632
T-cells	0.4151
B-cells	0.0912

SI Figure 31. Statistical analysis of differences in immune cell numbers from the peritoneal fluid of mice treated with or without therapy from F3 monoclonal antibody after thioglycollate injection (from **Fig. 3C**). Mice were injected IP with thioglycollate (1 mL of a 3% solution) and 48h later injected with 5mg/kg of F3 monoclonal antibody. After an additional 48h, mice were sacrificed, and IP fluid was removed, stained with various antibodies against cell-type markers and analyzed by flow cytometry. A t-test was used to determine if there were any significant differences in the number of different immune cell types between the F3 treated and non-treated animals. A p-value < 0.05 was considered significant and highlighted in yellow.

# All Macrophages	# M1-Macrophages Only	# M2-Macrophages Only

All Macrophages — Statistical Difference Between Healthy vs. F3 Treatments

	F3 (mg/kg)					
	Healthy	Thioglycollate				
	0	0	2.5	5	12.5	25
p-value	-	0.0048	0.0012	0.0271	0.0019	0.0001

M1-Macrophages Only — Statistical Difference Between Healthy vs. F3 Treatments

	F3 (mg/kg)					
	Healthy	Thioglycollate				
	0	0	2.5	5	12.5	25
p-value	-	0.0193	0.0054	0.0139	0.0510	0.0097

M2-Macrophages Only — Statistical Difference Between Healthy vs. F3 Treatments

	F3 (mg/kg)					
	Healthy	Thioglycollate				
	0	0	2.5	5	12.5	25
p-value	-	0.0022	0.0002	0.0216	0.0109	0.0004

All Macrophages — Statistical Difference Between F3 Treatments
1-Way Anova

	SS	df	MS	F	p
Between	104.3	4	26.07	5.7797	0.0020
Within	112.8	25	4.51		
Total	217.0	29			

M1-Macrophages Only — Statistical Difference Between F3 Treatments
1-Way Anova

	SS	df	MS	F	p
Between	41.0	4	10.25	4.3569	0.0082
Within	58.8	25	2.35		
Total	99.8	29			

M2-Macrophages Only — Statistical Difference Between F3 Treatments
1-Way Anova

	SS	df	MS	F	p
Between	17.7	4	4.43	4.9468	0.0045
Within	22.4	25	0.89		
Total	40.1	29			

All Macrophages — Tukey Test (p-value)
Thioglycollate treated
F3 (mg/kg)

	0	2.5	5	12.5	25
0	-	0.2350	0.0157	0.0028	0.0062
2.5		-	0.6699	0.2892	0.4586
5			-	0.9000	0.9000
12.5				-	0.9000
25					-

M1-Macrophages Only — Tukey Test (p-value)
Thioglycollate treated
F3 (mg/kg)

	0	2.5	5	12.5	25
0	-	0.2527	0.0165	0.0294	0.0138
2.5		-	0.6573	0.7932	0.6153
5			-	0.9000	0.9000
12.5				-	0.9000
25					-

M2-Macrophages Only — Tukey Test (p-value)
Thioglycollate treated
F3 (mg/kg)

	0	2.5	5	12.5	25
0	-	0.4974	0.0211	0.0024	0.0488
2.5		-	0.8938	0.0715	0.4277
5			-	0.2986	0.9000
12.5				-	0.6985
25					-

SI Figure 32. Statistical analysis of differences in macrophage numbers in peritoneal fluid of mice treated with and without F3 after thioglycollate injection (from **Fig. 3D**). Mice were injected IP with thioglycollate (1 mL of a 3% solution) and 48h later injected with various concentrations of F3 monoclonal antibody. After an additional 48h, mice were sacrificed, and IP fluid was removed, stained with various antibodies against macrophage-specific markers and analyzed by flow cytometry. A t-test was used to determine if there were any significant differences in the number of macrophages between healthy and Thioglycollate/F3 treated animals. A 1-way ANOVA was then performed to determine any differences between F3 treated groups. A p-value < 0.05 was considered significant and highlighted in yellow.

Collagen/Tuberculosis Bacteria RA Model

F3 Biodistribution

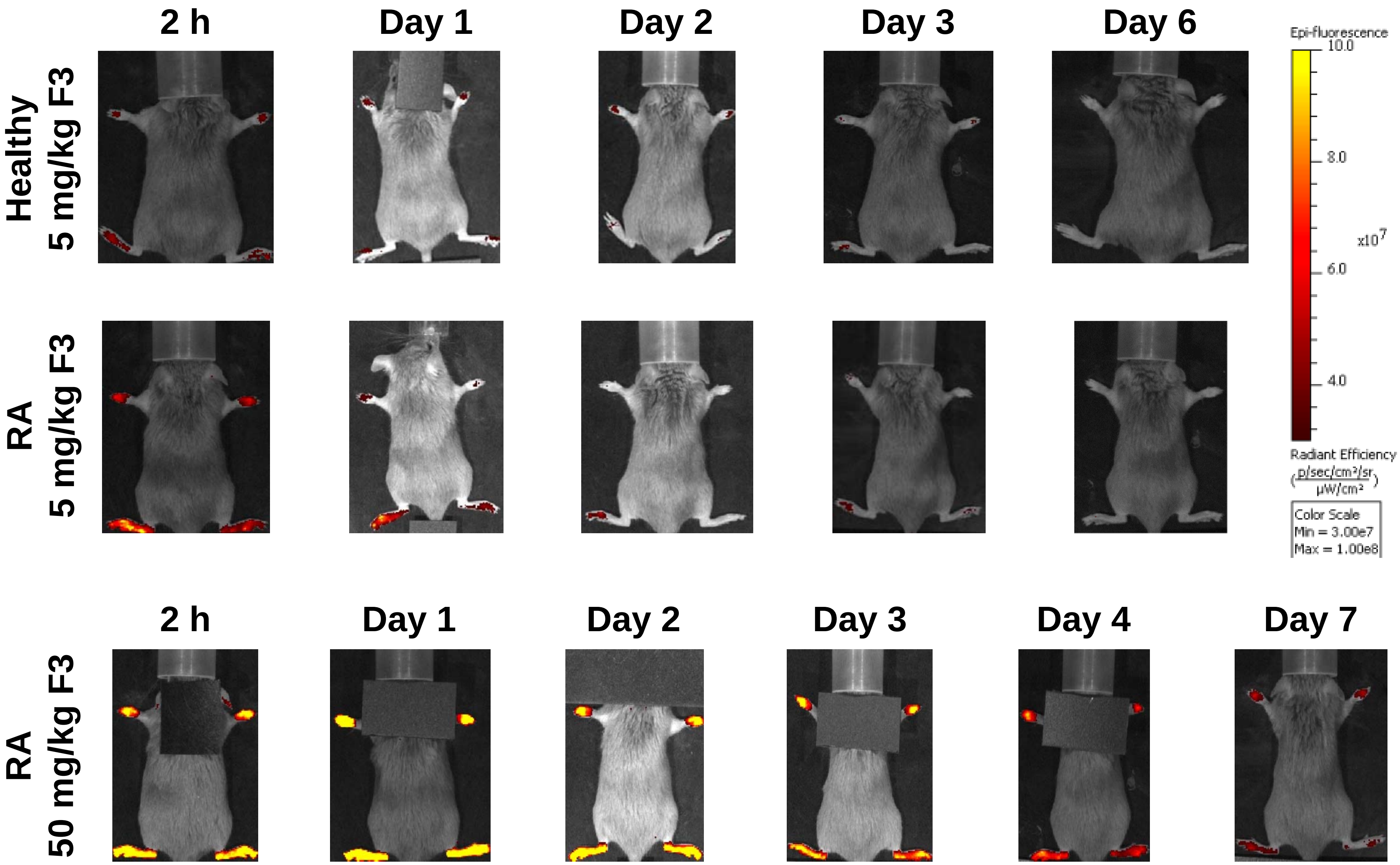

SI Figure 33. Time dependence of F3 uptake in arthritic mice treated to develop adjuvant-induced arthritis (from **Fig. 4B**). Adjuvant-induced arthritis was induced as described in Methods. Mice were injected intraperitoneally with AF647-conjugated F3 (5 or 50 mg/kg) when the arthritis score reached ~2. Whole animal fluorescence then was imaged at various times post-F3 injection.

Collagen/Tuberculosis Bacteria RA Model

Prophylactic Treatment

Arthritis Score and Weight

Healthy (Vehicle Control) - Arthritis Score

	Days																		
	0	1	2	3	4	5	6	7	8	9	10	11	12	13	14	15	16	17	18
n	4	4	4	4	4	4	4	4	4	4	4	4	4	4	4	4	4	4	4
average	0.00	0.00	0.00	0.00	0.00	0.00	0.00	0.00	0.00	0.00	0.00	0.00	0.00	0.00	0.00	0.00	0.00	0.00	0.00
standard deviation	0.000	0.000	0.000	0.000	0.000	0.000	0.000	0.000	0.000	0.000	0.000	0.000	0.000	0.000	0.000	0.000	0.000	0.000	0.000
standard error	0.000	0.000	0.000	0.000	0.000	0.000	0.000	0.000	0.000	0.000	0.000	0.000	0.000	0.000	0.000	0.000	0.000	0.000	0.000

RA (Vehicle Control) - Arthritis Score

	Days																		
	0	1	2	3	4	5	6	7	8	9	10	11	12	13	14	15	16	17	18
n	12	12	12	12	12	12	12	12	12	12	12	12	12	12	12	12	12	12	12
average	0.00	0.00	0.00	0.00	0.00	0.00	0.00	0.25	1.10	1.88	2.65	3.10	3.31	3.40	3.48	3.50	3.54	3.56	3.58
standard deviation	0.000	0.000	0.000	0.000	0.000	0.000	0.000	0.354	0.808	0.843	0.901	0.895	0.791	0.734	0.695	0.648	0.629	0.632	0.643
standard error	0.000	0.000	0.000	0.000	0.000	0.000	0.000	0.102	0.233	0.243	0.260	0.258	0.228	0.212	0.201	0.187	0.182	0.182	0.185

5 mg/kg F3 - Arthritis Score

	Days																		
	0	1	2	3	4	5	6	7	8	9	10	11	12	13	14	15	16	17	18
n	12	12	12	12	12	12	12	12	12	12	12	12	12	12	12	12	12	12	12
average	0.00	0.00	0.00	0.00	0.00	0.00	0.00	0.08	0.29	0.71	1.23	1.77	2.06	2.29	2.48	2.50	2.54	2.54	2.54
standard deviation	0.000	0.000	0.000	0.000	0.000	0.000	0.000	0.195	0.382	0.940	1.194	1.371	1.466	1.414	1.359	1.331	1.356	1.356	1.356
standard error	0.000	0.000	0.000	0.000	0.000	0.000	0.000	0.056	0.110	0.271	0.345	0.396	0.423	0.408	0.392	0.384	0.391	0.391	0.391

10 mg/kg F3 - Arthritis Score

	Days																		
	0	1	2	3	4	5	6	7	8	9	10	11	12	13	14	15	16	17	18
n	12	12	12	12	12	12	12	12	12	12	12	12	12	12	12	12	12	12	12
average	0.00	0.00	0.00	0.00	0.00	0.00	0.00	0.00	0.00	0.17	0.65	1.04	1.27	1.52	1.69	1.75	1.77	1.79	1.79
standard deviation	0.000	0.000	0.000	0.000	0.000	0.000	0.000	0.000	0.000	0.268	0.607	0.797	0.968	0.956	1.034	1.071	1.095	1.091	1.091
standard error	0.000	0.000	0.000	0.000	0.000	0.000	0.000	0.000	0.000	0.077	0.175	0.230	0.279	0.276	0.299	0.309	0.316	0.315	0.315

10 mg/kg Enbrel - Arthritis Score

	Days																		
	0	1	2	3	4	5	6	7	8	9	10	11	12	13	14	15	16	17	18
n	12	12	12	12	12	12	12	12	12	12	12	12	12	12	12	12	12	12	12
average	0.00	0.00	0.00	0.00	0.00	0.00	0.00	0.00	0.33	0.65	1.10	1.48	1.60	1.75	1.96	1.98	2.00	2.00	2.00
standard deviation	0.000	0.000	0.000	0.000	0.000	0.000	0.000	0.000	0.615	0.787	0.862	0.997	0.985	1.061	1.027	1.019	1.061	1.061	1.061
standard error	0.000	0.000	0.000	0.000	0.000	0.000	0.000	0.000	0.178	0.227	0.249	0.288	0.284	0.306	0.296	0.294	0.306	0.306	0.306

SI Figure 34. Comparison of Arthritis Scores following F3 or etanercept treatment of arthritic mice (from **Fig. 5A**). Arthritis was induced as described in methods and either F3 (5 or 10 mg/kg) or etanercept (10 mg/kg) was injected intraperitoneally 18 days later (day 0) before signs of arthritis were evident. Table provides arthritis scores as a function of time.

1-way ANOVA

	Day 7			Day 8			Day 9			Day 10			Day 11			Day 12		
SS (Between | Within | Total)	0.5	1.8	2.3	8.7	13.0	21.7	21.6	25.1	46.8	34.2	36.9	71.0	41.1	47.4	88.5	44.5	51.5	96.0
df (Between | Within | Total)	4	47	51	4	47	51	4	47	51	4	47	51	4	47	51	4	47	51
MS (Between | Within)	0.131	0.038		2.176	0.276		5.403	0.535		8.538	0.784		10.266	1.009		11.125	1.096	
F	3.447			7.896			10.101			10.888			10.179			10.148		
p-value	0.0150			5.93E-05			5.53E-06			2.50E-06			5.10E-06			5.26E-06		

post-hoc Tukey Test (p-value)

	Day 7	Day 8	Day 9	Day 10	Day 11	Day 12
Healthy vs. Disease	0.1910	0.0058	0.0010	0.0010	0.0010	0.0010
Healthy vs. F3 (5 mg/kg)	0.9000	0.8579	0.4598	0.1319	0.0291	0.0111
Healthy vs. F3 (10 mg/kg)	0.9000	0.9000	0.9000	0.6909	0.3889	0.2363
Healthy vs. Enbrel (10 mg/kg)	0.9000	0.7816	0.5432	0.2130	0.0966	0.0770
Disease vs. F3 (5 mg/kg)	0.2410	0.0037	0.0026	0.0025	0.0173	0.0404
Disease vs. F3 (10 mg/kg)	0.0235	0.0010	0.0010	0.0010	0.0010	0.0010
Disease vs. Enbrel (10 mg/kg)	0.0235	0.0066	0.0014	0.0010	0.0022	0.0020
F3 (5 mg/kg) vs. F3 (10 mg/kg)	0.8117	0.6367	0.3789	0.4963	0.3993	0.3575
F3 (5 mg/kg) vs. Enbrel (10 mg/kg)	0.8117	0.9000	0.9000	0.9000	0.9000	0.7969
F3 (10 mg/kg) vs. Enbrel (10 mg/kg)	0.9000	0.5289	0.5014	0.6884	0.7997	0.9000

	Day 13			Day 14			Day 15			Day 16			Day 17			Day 18		
SS (Between | Within | Total)	43.7	50.3	94.0	43.8	49.0	92.8	43.6	48.2	91.8	44.8	50.1	94.9	45.5	50.1	95.3	45.8	50.3	96.0
df (Between | Within | Total)	4	47	51	4	47	51	4	47	51	4	47	51	4	47	51	4	47	51
MS (Between | Within)	10.918	1.071		10.942	1.042		10.907	1.025		11.192	1.067		11.289	1.066		11.450	1.069	
F	10.193			10.499			10.640			10.491			10.590			10.709		
p-value	5.03E-06			3.69E-06			3.20E-06			3.72E-06			3.36E-06			2.98E-06		

post-hoc Tukey Test (p-value)

	Day 13	Day 14	Day 15	Day 16	Day 17	Day 18
Healthy vs. Disease	0.0010	0.0010	0.0010	0.0010	0.0010	0.0010
Healthy vs. F3 (5 mg/kg)	0.0033	0.0011	0.0010	0.0010	0.0010	0.0010
Healthy vs. F3 (10 mg/kg)	0.0979	0.0469	0.0339	0.0361	0.0329	0.0333
Healthy vs. Enbrel (10 mg/kg)	0.0399	0.0143	0.0120	0.0131	0.0130	0.0132
Disease vs. F3 (5 mg/kg)	0.0843	0.1332	0.1278	0.1411	0.1271	0.1159
Disease vs. F3 (10 mg/kg)	0.0010	0.0010	0.0010	0.0011	0.0011	0.0010
Disease vs. Enbrel (10 mg/kg)	0.0027	0.0057	0.0052	0.0056	0.0048	0.0042
F3 (5 mg/kg) vs. F3 (10 mg/kg)	0.3731	0.3318	0.3787	0.3710	0.3988	0.4004
F3 (5 mg/kg) vs. Enbrel (10 mg/kg)	0.6805	0.6984	0.6927	0.6791	0.6488	0.6798
F3 (10 mg/kg) vs. Enbrel (10 mg/kg)	0.9000	0.9000	0.9000	0.9000	0.9000	0.9000

SI Figure 35. Statistical analysis of differences in Arthritis Scores following F3 or etanercept treatment of arthritic mice (from **Fig. 5A**). Arthritis was induced as described in methods and either F3 (5 or 10 mg/kg) or etanercept (10 mg/kg) was injected intraperitoneally 18 days later (day 0) before signs of arthritis were evident. A 1-way ANOVA followed by a post-hoc Tukey test was used to determine if there were any significant differences in Arthritis Scores between groups. A p-value < 0.05 was considered significant and highlighted in yellow.

Healthy (Vehicle Control) - Body Weight (g)

	Days									
	0	2	4	6	8	10	12	14	16	18
n	4	4	4	4	4	4	4	4	4	4
average	24.61	24.68	24.83	24.98	24.18	24.72	24.56	25.29	25.33	25.61
standard deviation	0.843	0.918	1.118	0.907	1.385	1.290	1.533	1.240	1.207	1.392
standard error	0.421	0.459	0.559	0.453	0.693	0.645	0.766	0.620	0.604	0.696

RA (Vehicle Control) - Body Weight (g)

	Days									
	0	2	4	6	8	10	12	14	16	18
n	12	12	12	12	12	12	12	12	12	12
average	21.85	21.71	22.03	22.00	20.44	19.73	19.25	19.41	19.26	20.32
standard deviation	1.623	1.544	1.684	1.699	1.773	1.775	1.688	1.471	1.449	1.366
standard error	0.469	0.446	0.486	0.491	0.512	0.512	0.487	0.425	0.418	0.394

5 mg/kg F3 - Body Weight (g)

	Days									
	0	2	4	6	8	10	12	14	16	18
n	12	12	12	12	12	12	12	12	12	12
average	21.88	21.92	22.42	22.54	20.81	20.67	20.21	20.22	20.08	21.10
standard deviation	1.480	1.398	1.721	1.605	1.460	1.446	1.364	1.684	1.758	1.581
standard error	0.427	0.404	0.497	0.463	0.421	0.417	0.394	0.486	0.507	0.457

10 mg/kg F3 - Body Weight (g)

	Days									
	0	2	4	6	8	10	12	14	16	18
n	12	12	12	12	12	12	12	12	12	12
average	21.85	21.98	22.36	22.32	21.06	21.15	20.63	20.68	20.49	21.42
standard deviation	1.212	1.177	1.133	1.113	1.150	1.295	1.346	1.468	1.404	1.265
standard error	0.350	0.340	0.327	0.321	0.332	0.374	0.389	0.424	0.405	0.365

10 mg/kg Enbrel - Body Weight (g)

	Days									
	0	2	4	6	8	10	12	14	16	18
n	12	12	12	12	12	12	12	12	12	12
average	22.02	22.30	22.72	22.69	21.72	21.75	21.29	21.49	21.06	21.97
standard deviation	1.404	1.423	1.501	1.428	1.506	1.957	2.140	2.051	1.986	1.899
standard error	0.405	0.411	0.433	0.412	0.435	0.565	0.618	0.592	0.573	0.548

SI Figure 36. Comparison of body weight following F3 or etanercept treatment of arthritic mice (from **Fig. 5A**). Arthritis was induced as described in methods and either F3 (5 or 10 mg/kg) or etanercept (10 mg/kg) was injected intraperitoneally 18 days later (day 0) before signs of arthritis were evident. Table provides body weights as a function of time.

1-way ANOVA

	Day																													
	0			2			4			6			8			10			12			14			16			18		
SS (Between | Within | Total)	27.3	93.1	120.4	29.2	87.8	116.9	25.0	106.4	131.4	28.1	98.6	126.7	47.7	103.3	150.9	82.2	123.2	205.4	92.1	129.2	221.2	113.8	129.6	243.4	116.9	126.5	243.4	88.8	111.1	199.9
df (Between | Within | Total)	4	47	51	4	47	51	4	47	51	4	47	51	4	47	51	4	47	51	4	47	51	4	47	51	4	47	51	4	47	51
MS (Between | Within)	6.828	1.980		7.293	1.868		6.240	2.264		7.031	2.098		11.918	2.197		20.545	2.621		23.019	2.748		28.451	2.753		29.230	2.692		22.210	2.364	
F	3.448			3.905			2.756			3.351			5.424			7.838			8.377			10.319			10.858			9.397		
p-value	0.0150			0.0081			0.0386			0.0171			0.0011			0.0001			3.47E-05			4.43E-06			2.57E-06			1.15E-05		

post-hoc Tukey Test (p-value)

	0	2	4	6	8	10	12	14	16	18
Healthy vs. Disease	0.0118	0.0040	0.0190	0.0071	0.0010	0.0010	0.0010	0.0010	0.0010	0.0010
Healthy vs. F3 (5 mg/kg)	0.0129	0.0086	0.0584	0.0406	0.0024	0.0010	0.0110	0.0010	0.0010	0.0010
Healthy vs. F3 (10 mg/kg)	0.0116	0.0109	0.0489	0.0210	0.0057	0.0034	0.0014	0.0010	0.0010	0.0010
Healthy vs. Enbrel (10 mg/kg)	0.0207	0.0314	0.1268	0.0622	0.0458	0.0211	0.0010	0.0022	0.0010	0.0015
Disease vs. F3 (5 mg/kg)	0.9000	0.9000	0.9000	0.8820	0.9000	0.6069	0.0318	0.7234	0.7119	0.7008
Disease vs. F3 (10 mg/kg)	0.9000	0.9000	0.9000	0.9000	0.8188	0.2220	0.2627	0.3458	0.3670	0.4160
Disease vs. Enbrel (10 mg/kg)	0.9000	0.9000	0.7686	0.7436	0.2270	0.0291	0.6068	0.0280	0.7045	0.0809
F3 (5 mg/kg) vs. F3 (10 mg/kg)	0.9000	0.9000	0.9000	0.9000	0.9000	0.9000	0.8493	0.9000	0.9000	0.9000
F3 (5 mg/kg) vs. Enbrel (10 mg/kg)	0.9000	0.9000	0.9000	0.9000	0.5597	0.4814	0.5016	0.3512	0.5780	0.6227
F3 (10 mg/kg) vs. Enbrel (10 mg/kg)	0.9000	0.9000	0.9000	0.9000	0.7859	0.8832	0.9000	0.7289	0.9000	0.9000

SI Figure 37. Statistical analysis of differences in body weight following F3 or etanercept treatment of arthritic mice (from **Fig. 5A**). Arthritis was induced as described in methods and either F3 (5 or 10 mg/kg) or etanercept (10 mg/kg) was injected intraperitoneally 18 days later (day 0) before signs of arthritis were evident. A 1-way ANOVA followed by a post-hoc Tukey test was used to determine if there were any significant differences in body weight between groups. A p-value < 0.05 was considered significant and highlighted in yellow.

Collagen/Tuberculosis Bacteria RA Model

Restorative Treatment – Dose Finding/Pilot Study

Paw Thickness and Weight

Healthy (Vehicle Control) - Paw Thickness (mm)

	Days																
	1	2	3	4	5	6	7	8	9	10	11	12	13	14	15	16	17
n	1	1	1	1	1	1	1	1	1	1	1	1	1	1	1	1	1
average	1.18	1.17	1.16	1.16	1.11	1.17	1.14	1.10	1.14	1.07	1.11	1.12	1.19	1.20	1.19	1.19	1.20
standard deviation	-	-	-	-	-	-	-	-	-	-	-	-	-	-	-	-	-
standard error	-	-	-	-	-	-	-	-	-	-	-	-	-	-	-	-	-

RA (Vehicle Control) - Paw Thickness (mm)

	Days																
	1	2	3	4	5	6	7	8	9	10	11	12	13	14	15	16	17
n	3	3	3	3	3	3	3	3	3	3	3	3	3	3	3	3	3
average	1.45	1.55	1.60	1.54	1.68	1.63	1.71	1.79	1.85	1.87	1.85	1.86	1.94	1.99	1.95	1.95	1.93
standard deviation	0.08	0.26	0.18	0.30	0.28	0.26	0.19	0.23	0.34	0.14	0.15	0.10	0.05	0.10	0.05	0.18	0.18
standard error	0.05	0.15	0.11	0.17	0.16	0.15	0.11	0.14	0.20	0.08	0.09	0.06	0.03	0.06	0.03	0.10	0.10

5 mg/kg F3

	Days																
	1	2	3	4	5	6	7	8	9	10	11	12	13	14	15	16	17
n	3	3	3	3	3	3	3	3	3	3	3	3	3	3	3	3	3
average	1.6	1.4	1.5	1.5	1.6	1.5	1.5	1.6	1.6	1.6	1.6	1.6	1.6	1.6	1.7	1.8	1.7
standard deviation	0.18	0.24	0.24	0.23	0.29	0.20	0.31	0.36	0.20	0.40	0.22	0.25	0.04	0.17	0.23	0.13	0.19
standard error	0.10	0.14	0.14	0.13	0.17	0.11	0.18	0.21	0.12	0.23	0.13	0.14	0.02	0.10	0.13	0.08	0.11

10 mg/kg F3

	Days																
	1	2	3	4	5	6	7	8	9	10	11	12	13	14	15	16	17
n	3	3	3	3	3	3	3	3	3	3	3	3	3	3	3	3	3
average	1.41	1.40	1.41	1.39	1.42	1.30	1.30	1.35	1.42	1.33	1.41	1.39	1.33	1.44	1.41	1.38	1.44
standard deviation	0.13	0.24	0.11	0.07	0.14	0.10	0.11	0.12	0.12	0.15	0.06	0.20	0.08	0.22	0.21	0.20	0.14
standard error	0.08	0.14	0.06	0.04	0.08	0.06	0.07	0.07	0.07	0.08	0.04	0.12	0.04	0.13	0.12	0.11	0.08

20 mg/kg F3

	Days																
	1	2	3	4	5	6	7	8	9	10	11	12	13	14	15	16	17
n	3	3	3	3	3	3	3	3	3	3	3	3	3	3	3	3	3
average	1.42	1.40	1.34	1.22	1.26	1.26	1.34	1.28	1.21	1.14	1.19	1.25	1.20	1.22	1.23	1.19	1.18
standard deviation	0.03	0.01	0.01	0.04	0.01	0.09	0.03	0.07	0.10	0.03	0.05	0.00	0.06	0.06	0.02	0.01	0.06
standard error	0.02	0.01	0.01	0.02	0.01	0.05	0.02	0.04	0.06	0.02	0.03	0.00	0.04	0.04	0.01	0.00	0.04

SI Figure 38. Comparison of paw thickness following F3 treatment in mice induced to develop arthritis (from **Fig. 5B**). Arthritis was induced as described in methods and F3 (5, 10 or 20 mg/kg) was injected intraperitoneally (day 1) only after arthritis scores reached ~2. Table provides paw thickness measurements as a function of time.

1-way ANOVA - Paw Thickness

	Day 1	Day 2	Day 3	Day 4	Day 5	Day 6	Day 7	Day 8	Day 9
SS (Between | Within | Total)	0.05 0.11 0.16	0.05 0.36 0.42	0.10 0.21 0.31	0.16 0.29 0.45	0.26 0.37 0.62	0.24 0.24 0.48	0.29 0.29 0.58	0.44 0.40 0.84	0.57 0.35 0.92
df (Between | Within | Total)	3 8 11	3 8 11	3 8 11	3 8 11	3 8 11	3 8 11	3 8 11	3 8 11	3 8 11
MS (Between | Within)	0.015 0.016	0.017 0.052	0.032 0.030	0.053 0.042	0.086 0.052	0.082 0.034	0.098 0.042	0.146 0.057	0.190 0.051
F	0.926	0.334	1.080	1.250	1.647	2.391	2.352	2.542	3.749
p-value	0.4764	0.8013	0.4175	0.3621	0.2638	0.1543	0.1585	0.1396	0.0681

post-hoc Tukey Test (p-value)

	1	2	3	4	5	6	7	8	9
Disease vs. F3 (5 mg/kg)	-	-	-	-	-	-	-	-	-
Disease vs. F3 (10 mg/kg)	-	-	-	-	-	-	-	-	-
Disease vs. F3 (20 mg/kg)	-	-	-	-	-	-	-	-	-
F3 (5 mg/kg) vs. F3 (10 mg/kg)	-	-	-	-	-	-	-	-	-
F3 (5 mg/kg) vs. F3 (20 mg/kg)	-	-	-	-	-	-	-	-	-
F3 (10 mg/kg) vs. F3 (20 mg/kg)	-	-	-	-	-	-	-	-	-

1-way ANOVA - Paw Thickness

	Day 10	Day 11	Day 12	Day 13	Day 14	Day 15	Day 16	Day 17
SS (Between | Within | Total)	0.76 0.39 1.16	0.60 0.16 0.75	0.56 0.23 0.78	0.84 0.02 0.86	0.82 0.18 1.00	0.78 0.20 0.97	0.96 0.17 1.13	0.80 0.18 0.98
df (Between | Within | Total)	3 8 11	3 8 11	3 8 11	3 8 11	3 8 11	3 8 11	3 8 11	3 8 11
MS (Between | Within)	0.254 0.056	0.199 0.022	0.186 0.032	0.279 0.004	0.273 0.026	0.260 0.028	0.319 0.025	0.267 0.025
F	4.503	8.853	5.767	80.273	10.494	9.257	12.786	10.536
p-value	0.0463	0.0088	0.0263	8.72E-06	0.0055	0.0078	0.0032	0.0055

post-hoc Tukey Test (p-value)

	10	11	12	13	14	15	16	17
Disease vs. F3 (5 mg/kg)	0.4397	0.2439	0.3305	0.0010	0.1257	0.2820	0.6225	0.0430
Disease vs. F3 (10 mg/kg)	0.1020	0.0359	0.0571	0.0010	0.0177	0.0223	0.0126	0.0269
Disease vs. F3 (20 mg/kg)	0.0467	0.0077	0.0282	0.0010	0.0052	0.0086	0.0049	0.0056
F3 (5 mg/kg) vs. F3 (10 mg/kg)	0.6521	0.4964	0.5533	0.0054	0.4678	0.2820	0.0577	0.2169
F3 (5 mg/kg) vs. F3 (20 mg/kg)	0.2956	0.0763	0.2378	0.0010	0.0877	0.0761	0.0172	0.0301
F3 (10 mg/kg) vs. F3 (20 mg/kg)	0.7931	0.4077	0.7930	0.1366	0.4835	0.6260	0.5844	0.3676

SI Figure 39. Statistical analysis of differences in paw thicknesses following F3 treatment in mice induced to develop arthritis (from **Fig. 5B**). Arthritis was induced as described in methods and F3 (5, 10 or 20 mg/kg) was injected intraperitoneally (day 1) only after arthritis scores reached ~2. A 1-way ANOVA followed by a post-hoc Tukey test was used to determine if there were any significant differences in paw thickness between groups. A p-value < 0.05 was considered significant and highlighted in yellow.

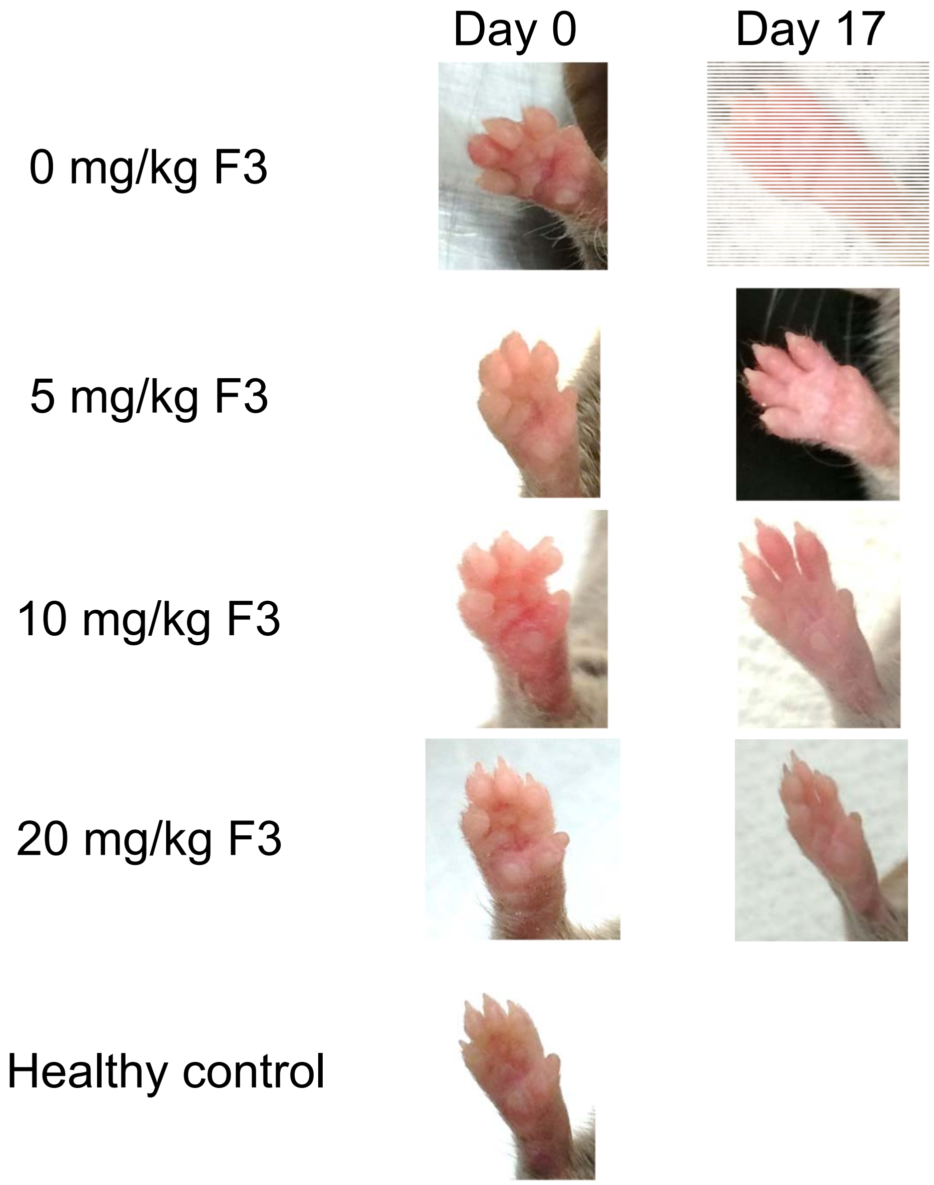

SI Figure 40. Images of paws from F3-treated and untreated mice induced to develop arthritis (from **Fig. 5B**). Arthritis was induced as described in methods and F3 (5, 10 or 20 mg/kg) was injected intraperitoneally (day 1) only after arthritis scores reached ~2. Images of paws were acquired on Day 0 and Day 17.

Healthy (Vehicle Control) - Body Weight (g)

	Days																
	1	2	3	4	5	6	7	8	9	10	11	12	13	14	15	16	17
n	1	1	1	1	1	1	1	1	1	1	1	1	1	1	1	1	1
average	17	16.9	16.9	16.8	16.8	16.5	16.6	16.4	16.7	16.7	16.9	16.2	16.6	17	17	16	16.2
standard deviation	-	-	-	-	-	-	-	-	-	-	-	-	-	-	-	-	-
standard error	-	-	-	-	-	-	-	-	-	-	-	-	-	-	-	-	-

RA (Vehicle Control) - Body Weight (g)

	Days																
	1	2	3	4	5	6	7	8	9	10	11	12	13	14	15	16	17
n	3	3	3	3	3	3	3	3	3	3	3	3	3	3	3	3	3
average	18.8	18.5	18.5	18.5	18.2	17.9	17.7	17.6	17.6	17.1	17.1	17.0	16.8	17.1	16.9	17.1	17.3
standard deviation	0.83	0.85	0.64	0.40	0.21	0.59	0.30	0.21	0.72	0.20	0.72	0.69	0.57	0.55	0.76	0.81	0.82
standard error	0.48	0.49	0.37	0.23	0.12	0.34	0.17	0.12	0.42	0.12	0.42	0.40	0.33	0.32	0.44	0.47	0.47

5 mg/kg F3

	Days																
	1	2	3	4	5	6	7	8	9	10	11	12	13	14	15	16	17
n	3	3	3	3	3	3	3	3	3	3	3	3	3	3	3	3	3
average	18.8	18.4	18.0	18.0	18.2	18.4	18.5	18.3	18.6	18.4	17.8	17.8	17.8	18.0	17.8	17.7	17.8
standard deviation	1.17	1.58	1.59	1.55	1.62	1.61	1.42	1.82	2.01	1.35	1.50	1.85	2.14	2.27	2.57	2.42	2.32
standard error	0.67	0.91	0.92	0.90	0.93	0.93	0.82	1.05	1.16	0.78	0.87	1.07	1.23	1.31	1.48	1.40	1.34

10 mg/kg F3

	Days																
	1	2	3	4	5	6	7	8	9	10	11	12	13	14	15	16	17
n	3	3	3	3	3	3	3	3	3	3	3	3	3	3	3	3	3
average	18.6	18.3	18.1	18.1	17.9	17.9	17.8	17.8	18.1	17.8	17.6	17.5	17.9	17.7	17.7	17.5	17.5
standard deviation	0.85	0.99	0.96	1.04	0.98	1.22	1.06	1.21	1.93	1.36	1.21	1.37	1.37	1.60	1.26	1.12	1.02
standard error	0.49	0.57	0.56	0.60	0.57	0.70	0.61	0.70	1.11	0.78	0.70	0.79	0.79	0.92	0.73	0.65	0.59

20 mg/kg F3

	Days																
	1	2	3	4	5	6	7	8	9	10	11	12	13	14	15	16	17
n	3	3	3	3	3	3	3	3	3	3	3	3	3	3	3	3	3
average	18.2	17.8	17.8	17.6	17.7	17.5	17.1	16.8	17.1	16.8	16.9	16.5	16.2	16.2	16.2	16.2	16.0
standard deviation	0.07	0.92	1.13	0.49	1.13	1.06	0.71	0.35	0.28	1.06	0.49	0.49	0.35	0.07	0.07	0.07	0.07
standard error	0.04	0.53	0.65	0.29	0.65	0.61	0.41	0.20	0.16	0.61	0.29	0.29	0.20	0.04	0.04	0.04	0.04

SI Figure 41. Comparison of body weight following F3 treatment in mice induced to develop arthritis (from **Fig. 5B**). Arthritis was induced as described in methods and F3 (5, 10 or 20 mg/kg) was injected intraperitoneally (day 1) only after arthritis scores reached ~2. Table provides body weight measurements as a function of time.

1-way ANOVA - Body Weight

	Day																										
	1			2			3			4			5			6			7			8			9		
SS (Between | Within | Total)	0.64	5.57	6.21	0.78	9.25	10.02	0.59	9.05	9.64	1.05	7.56	8.61	0.48	8.52	9.00	1.11	9.95	11.07	2.34	6.97	9.30	2.99	9.76	12.75	3.04	16.65	19.70
df (Between | Within | Total)	3	8	11	3	8	11	3	8	11	3	8	11	3	8	11	3	8	11	3	8	11	3	8	11	3	8	11
MS (Between | Within)	0.214	0.795		0.259	1.321		0.196	1.293		0.349	1.080		0.159	1.217		0.371	1.422		0.778	0.995		0.997	1.394		1.014	2.379	
F	0.269			0.196			0.152			0.323			0.131			0.261			0.782			0.715			0.426		
p-value	0.8462			0.8958			0.9253			0.8088			0.9389			0.8513			0.5406			0.5735			0.7404		

	post-hoc Tukey Test (p-value)								
Disease vs. F3 (5 mg/kg)	-	-	-	-	-	-	-	-	-
Disease vs. F3 (10 mg/kg)	-	-	-	-	-	-	-	-	-
Disease vs. F3 (20 mg/kg)	-	-	-	-	-	-	-	-	-
F3 (5 mg/kg) vs. F3 (10 mg/kg)	-	-	-	-	-	-	-	-	-
F3 (5 mg/kg) vs. F3 (20 mg/kg)	-	-	-	-	-	-	-	-	-
F3 (10 mg/kg) vs. F3 (20 mg/kg)	-	-	-	-	-	-	-	-	-

1-way ANOVA - Body Weight

	Day																							
	10			11			12			13			14			15			16			17		
SS (Between | Within | Total)	4.19	8.51	12.70	1.44	8.72	10.16	2.46	11.83	14.29	5.22	13.68	18.90	4.89	16.02	20.91	4.03	17.57	21.60	3.28	15.57	18.85	4.65	14.18	18.83
df (Between | Within | Total)	3	8	11	3	8	11	3	8	11	3	8	11	3	8	11	3	8	11	3	8	11	3	8	11
MS (Between | Within)	1.397	1.216		0.479	1.246		0.819	1.690		1.741	1.954		1.629	2.288		1.344	2.509		1.095	2.224		1.549	2.026	
F	1.149			0.385			0.484			0.891			0.712			0.536			0.492			0.765		
p-value	0.3940			0.7675			0.7037			4.91E-01			0.5752			0.6726			0.6988			0.5489		

	post-hoc Tukey Test (p-value)							
Disease vs. F3 (5 mg/kg)	-	-	-	-	-	-	-	-
Disease vs. F3 (10 mg/kg)	-	-	-	-	-	-	-	-
Disease vs. F3 (20 mg/kg)	-	-	-	-	-	-	-	-
F3 (5 mg/kg) vs. F3 (10 mg/kg)	-	-	-	-	-	-	-	-
F3 (5 mg/kg) vs. F3 (20 mg/kg)	-	-	-	-	-	-	-	-
F3 (10 mg/kg) vs. F3 (20 mg/kg)	-	-	-	-	-	-	-	-

SI Figure 42. Statistical analysis of differences in body weight following F3 treatment in mice induced to develop arthritis (from **Fig. 5B**). Arthritis was induced as described in methods and F3 (5, 10 or 20 mg/kg) was injected intraperitoneally (day 1) only after arthritis scores reached ~2. A 1-way ANOVA followed by a post-hoc Tukey test was used to determine if there were any significant differences in body weight between groups. A p-value < 0.05 was considered significant and highlighted in yellow.

Collagen/Tuberculosis Bacteria RA Model

Restorative Treatment – Full Study

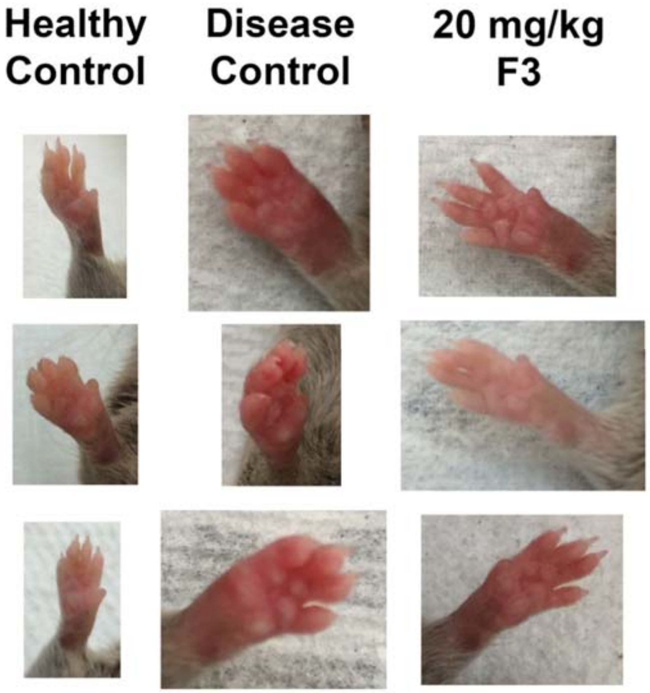

SI Figure 43. Images of paws from F3-treated and untreated mice induced to develop arthritis (from **Fig. 6**). Arthritis was induced as described in methods and F3 (20 mg/kg) was injected intraperitoneally (day 1) only after arthritis scores reached ~4. Images of paws were acquired on Day 0 and Day 18.

Healthy (Vehicle Control) - Paw Thickness (mm)

	Days								
	1	3	5	7	9	11	13	15	17
n	6	6	6	6	6	6	6	6	6
average	1.21	1.18	1.27	1.19	1.19	1.14	1.18	1.20	1.22
standard deviation	0.07	0.03	0.10	0.03	0.04	0.02	0.03	0.04	0.04
standard error	0.03	0.01	0.04	0.01	0.02	0.01	0.01	0.02	0.02

RA (Vehicle Control) - Paw Thickness (mm)

	Days								
	1	3	5	7	9	11	13	15	17
n	10	10	10	10	10	10	10	10	10
average	1.53	1.78	1.86	1.89	2.15	2.22	2.38	2.50	2.59
standard deviation	0.11	0.24	0.40	0.34	0.52	0.46	0.47	0.44	0.45
standard error	0.04	0.08	0.13	0.11	0.16	0.15	0.15	0.14	0.14

20 mg/kg F3 - Paw Thickness (mm)

	Days								
	1	3	5	7	9	11	13	15	17
n	9	9	9	9	9	9	9	9	9
average	1.61	1.51	1.54	1.53	1.54	1.46	1.51	1.48	1.43
standard deviation	0.20	0.16	0.23	0.18	0.23	0.18	0.23	0.19	0.23
standard error	0.07	0.05	0.08	0.06	0.08	0.06	0.08	0.06	0.08

SI Figure 44. Comparison of paw thickness following F3 treatment in mice induced to develop arthritis (from **Fig. 6A**). Arthritis was induced as described in methods and F3 (20 mg/kg) was injected intraperitoneally (day 1) only after arthritis scores reached ~4. Table provides paw thickness measurements as a function of time.

1-way ANOVA - Paw Thickness

	Day 1			Day 3			Day 5			Day 7			Day 9			Day 11			Day 13			Day 15			Day 17		
SS (Between | Within | Total)	0.6	0.5	1.1	1.3	0.7	2.1	1.4	1.9	3.3	1.9	1.3	3.2	3.9	2.8	6.7	5.1	2.2	7.3	6.4	2.4	8.8	7.9	2.0	9.9	8.5	1.9	10.4
df (Between | Within | Total)	2	22	24	2	22	24	2	22	24	2	22	24	2	22	24	2	22	24	2	22	24	2	22	24	2	22	24
MS (Between | Within)	0.322	0.021		0.664	0.034		0.677	0.087		0.953	0.060		1.929	0.130		2.530	0.100		3.200	0.108		3.953	0.091		4.251	0.102	
F	15.538			19.610			7.763			15.791			14.894			25.336			29.654			43.508			41.743		
p-value	6.21E-05			1.29E-05			2.80E-03			5.59E-05			8.13E-05			1.96E-06			5.69E-07			2.26E-08			1.11E-07		

post-hoc Tukey Test (p-value)

	Day 1	Day 3	Day 5	Day 7	Day 9	Day 11	Day 13	Day 15	Day 17
Healthy vs. Disease	0.0010	0.0010	0.0024	0.0010	0.0010	0.0010	0.0010	0.0010	0.0010
Healthy vs. F3 (20 mg/kg)	0.0010	0.0071	0.2265	0.0434	0.1806	0.1666	0.1613	0.2022	0.4754
Disease vs. F3 (20 mg/kg)	0.4666	0.0132	0.0660	0.0098	0.0033	0.0010	0.0010	0.0010	0.0010

SI Figure 45. Statistical analysis of differences in paw thickness following F3 treatment in mice induced to develop arthritis (from **Fig. 6A**). Arthritis was induced as described in methods and F3 (20 mg/kg) was injected intraperitoneally (day 1) only after arthritis scores reached ~4. A 1-way ANOVA followed by a post-hoc Tukey test was used to determine if there were any significant differences between groups. A p-value < 0.05 was considered significant and highlighted in yellow.

Healthy (Vehicle Control) - Arthritis Score

	Days								
	1	3	5	7	9	11	13	15	17
n	6	6	6	6	6	6	6	6	6
average	0.00	0.00	0.00	0.00	0.00	0.00	0.00	0.00	0.00
standard deviation	0.00	0.00	0.00	0.00	0.00	0.00	0.00	0.00	0.00
standard error	0.00	0.00	0.00	0.00	0.00	0.00	0.00	0.00	0.00

RA (Vehicle Control) - Arthritis Score

	Days								
	1	3	5	7	9	11	13	15	17
n	7	7	7	7	7	7	7	7	7
average	4.91	6.41	7.36	8.20	10.14	10.91	12.14	11.81	10.93
standard deviation	1.40	1.84	2.88	2.92	3.77	2.81	2.03	2.41	1.13
standard error	0.53	0.69	1.09	1.11	1.43	1.06	0.77	0.91	0.43

20 mg/kg F3 - Arthritis Score

	Days								
	1	3	5	7	9	11	13	15	17
n	8	8	8	8	8	8	8	8	8
average	3.99	3.25	3.69	2.79	3.48	2.40	2.69	2.29	2.47
standard deviation	0.93	1.28	2.13	2.48	2.42	2.13	2.19	2.82	2.36
standard error	0.33	0.45	0.75	0.88	0.86	0.75	0.77	1.00	0.83

SI Figure 46. Comparison of Arthritis Score following F3 treatment in mice induced to develop arthritis (from **Fig. 6B**). Arthritis was induced as described in methods and F3 (20 mg/kg) was injected intraperitoneally (day 1) only after arthritis scores reached ~4. Table provides Arthritis Score measurements as a function of time.

1-way ANOVA - Arthritis Score

	Day																										
	1			3			5			7			9			11			13			15			17		
SS (Between | Within | Total)	86.9	17.8	104.7	133.1	31.8	164.9	175.2	81.5	256.8	230.4	94.2	324.6	351.9	126.4	478.3	444.7	79.1	523.9	549.8	58.1	607.9	533.1	90.8	623.9	433.8	35.5	469.3
df (Between | Within | Total)	2	18	20	2	18	20	2	18	20	2	18	20	2	18	20	2	18	20	2	18	20	2	18	20	2	18	20
MS (Between | Within)	43.467	0.987		66.565	1.765		87.623	4.528		115.180	5.234		175.959	7.022		222.362	4.397		274.909	3.227		266.570	5.042		216.911	2.221	
F	44.061			37.715			19.351			22.007			25.059			50.570			85.190			52.869			97.687		
p-value	1.16E-07			3.66E-07			3.27E-05			1.46E-05			6.28E-06			4.10E-08			6.64E-10			2.92E-08			1.08E-09		

post-hoc Tukey Test (p-value)

	1	3	5	7	9	11	13	15	17
Healthy vs. Disease	0.0010	0.0010	0.0010	0.0010	0.0010	0.0010	0.0010	0.0010	0.0010
Healthy vs. F3 (20 mg/kg)	0.0010	0.0010	0.0128	0.0884	0.0637	0.1139	0.0321	0.1713	0.0285
Disease vs. F3 (20 mg/kg)	0.1968	0.0010	0.0098	0.0010	0.0010	0.0010	0.0010	0.0010	0.0010

SI Figure 47. Statistical analysis of differences in Arthritis Score following F3 treatment in mice induced to develop arthritis (from **Fig. 6B**). Arthritis was induced as described in methods and F3 (20 mg/kg) was injected intraperitoneally (day 1) only after arthritis scores reached ~4. A 1-way ANOVA followed by a post-hoc Tukey test was used to determine if there were any significant differences between groups. A p-value < 0.05 was considered significant and highlighted in yellow.

Bone Density (BV/TV)

1-Way Anova

	SS	df	MS	F	p
Between	0.1588	2	0.0794	76.401	1.49E-07
Within	0.0125	12	0.0010		
Total	0.1713	14			

Tukey Test (p-value)

	Healthy	Disease	F3
Healthy	-	0.0010	0.5333
Disease		-	0.0010
F3			-

Connectivity Density

1-Way Anova

	SS	df	MS	F	p
Between	9,120,858	2	4,560,429	68.39	2.75E-07
Within	800,212	12	66,684		
Total	9,921,071	14			

Tukey Test (p-value)

	Healthy	Disease	F3
Healthy	-	0.0010	0.2218
Disease		-	0.0010
F3			-

Structure Model Index

1-Way Anova

	SS	df	MS	F	p
Between	11.78	2	5.8912	110.29	1.89E-08
Within	0.64	12	0.0534		
Total	12.42	14			

Tukey Test (p-value)

	Healthy	Disease	F3
Healthy	-	0.0010	0.8543
Disease		-	0.0010
F3			-

Trabecular Thickness

1-Way Anova

	SS	df	MS	F	p
Between	0	2	0	0.6759	0.5270
Within	0.0003	12	0		
Total	0.0003	14			

Trabecular Spacing

1-Way Anova

	SS	df	MS	F	p
Between	0.0010	2	0.0005	0.5299	0.6018
Within	0.0111	12	0.0009		
Total	0.0120	14			

SI Figure 48. Statistical analysis of differences in morphometric bone parameters following F3 treatment in mice induced to develop arthritis (from **Fig. 6D-6H**). Arthritis was induced as described in methods and F3 (20 mg/kg) was injected intraperitoneally (day 1) only after arthritis scores reached ~4. A 1-way ANOVA followed by a post-hoc Tukey test was used to determine if there were any significant differences between groups. A p-value < 0.05 was considered significant and highlighted in yellow.

Relative Intensity

	Healthy Control	Disease Control	20 mg/kg F3
n	6	6	6
average	1.92	77.17	1.67
standard deviation	3.24	41.36	2.61
standard error	1.32	16.89	1.06

1-Way Anova

	SS	df	MS	F	p
Between	22725	2	11363	19.727	6.32E-05
Within	8640	15	576		
Total	31365	17			

Tukey Test (p-value)

	Healthy	Disease	F3
Healthy	-	0.0010	0.9000
Disease		-	0.0010
F3			-

SI Figure 49. Comparison and statistical analysis of differences in uptake of a ^{99m}Tc-folate receptor-targeted imaging agent (EC20; 75 nmol/kg) following F3 treatment in mice induced to develop arthritis (from **Fig. 6I**). Arthritis was induced as described in methods and F3 (20 mg/kg) was injected intraperitoneally (day 1) only after arthritis scores reached ~4. Table provides the relative intensity on day 18. A 1-way ANOVA followed by a post-hoc Tukey test was used to determine if there were any significant differences between groups. A p-value < 0.05 was considered significant and highlighted in yellow.

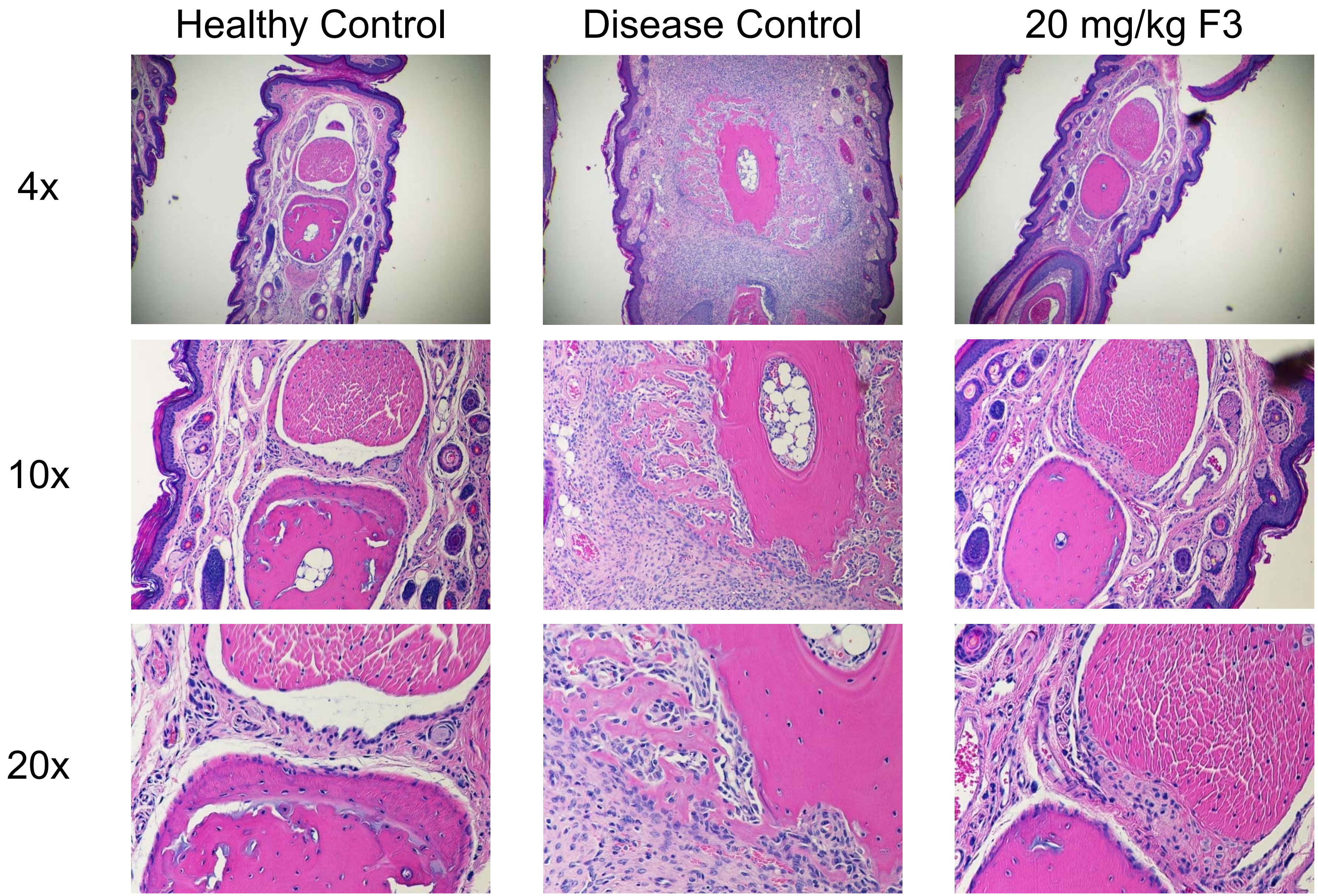

SI Figure 50. H&E staining of joints following F3 treatment in mice induced to develop arthritis (from **Fig. 6J**). Arthritis was induced as described in methods and F3 (20 mg/kg) was injected intraperitoneally (day 1) only after arthritis scores reached ~4. At the end of the study (day 18), sections of joint tissue were H&E stained and imaged at various magnifications.

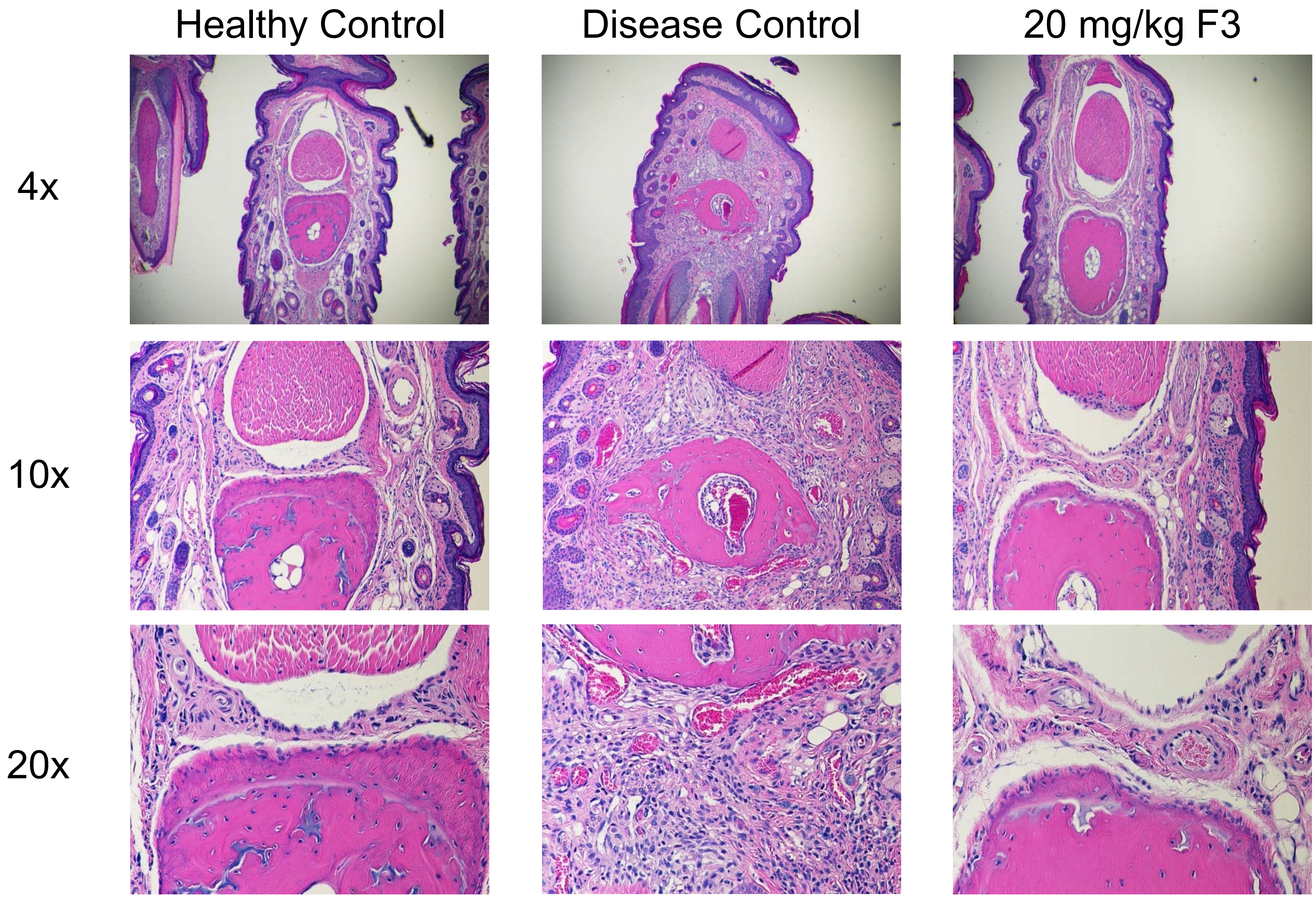

SI Figure 51. H&E staining of joints following F3 treatment in mice induced to develop arthritis (from **Fig. 6J**). Arthritis was induced as described in methods and F3 (20 mg/kg) was injected intraperitoneally (day 1) only after arthritis scores reached ~4. At the end of the study (day 18), sections of joint tissue were H&E stained and imaged at various magnifications.

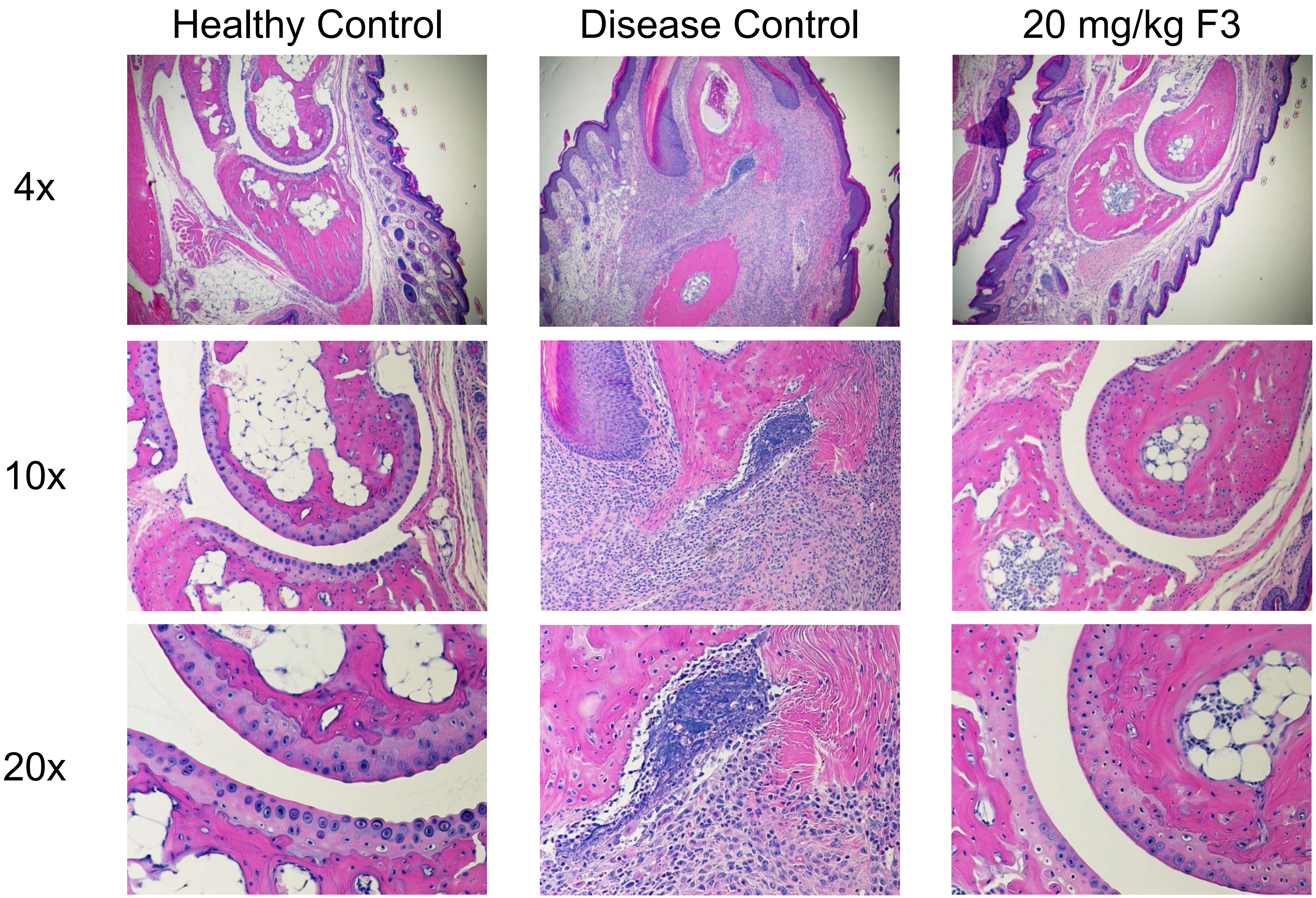

SI Figure 52. H&E staining of joints following F3 treatment in mice induced to develop arthritis (from **Fig. 6J**). Arthritis was induced as described in methods and F3 (20 mg/kg) was injected intraperitoneally (day 1) only after arthritis scores reached ~4. At the end of the study (day 18), sections of joint tissue were H&E stained and imaged at various magnifications.

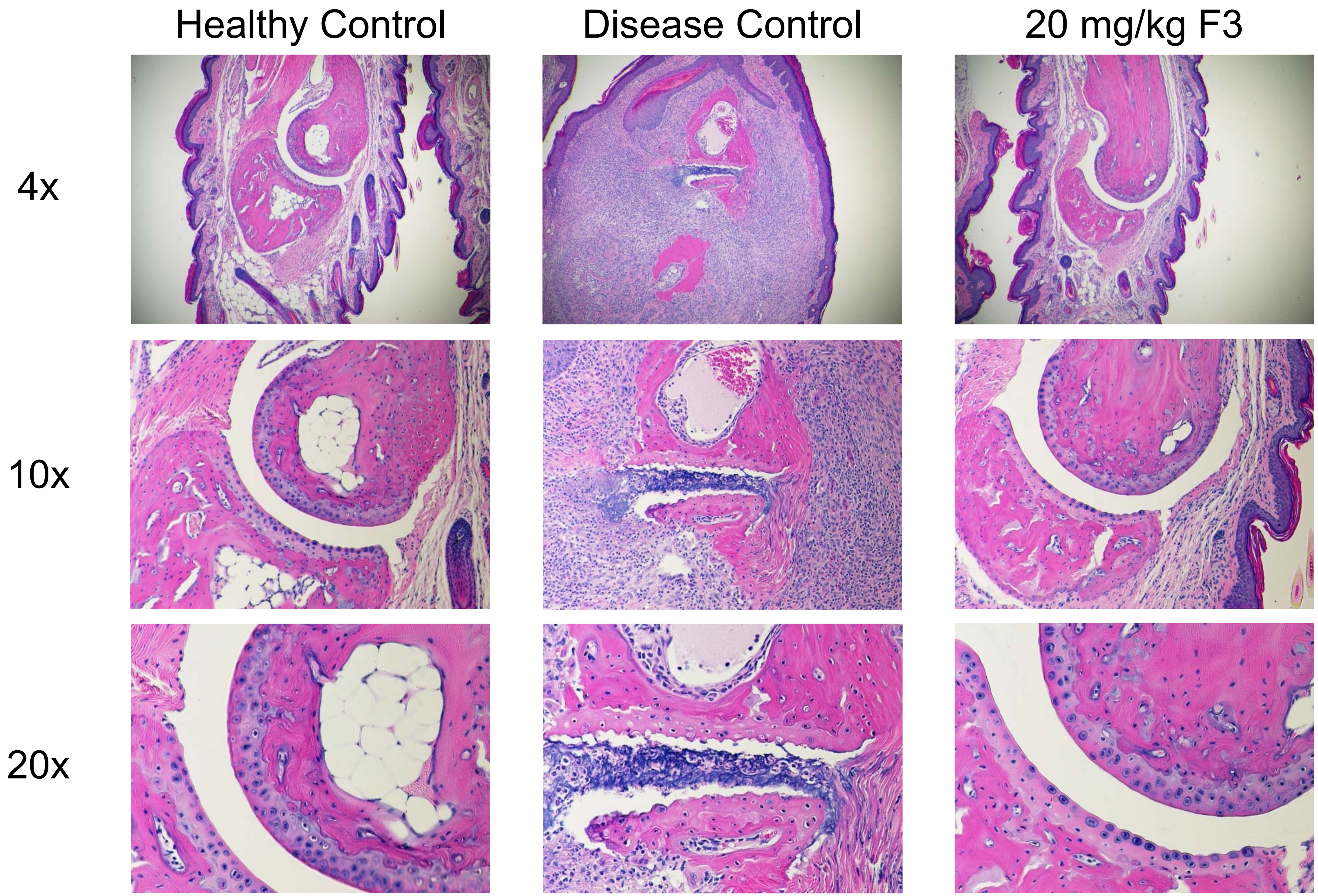

SI Figure 53. H&E staining of joints following F3 treatment in mice induced to develop arthritis (from **Fig. 6J**). Arthritis was induced as described in methods and F3 (20 mg/kg) was injected intraperitoneally (day 1) only after arthritis scores reached ~4. At the end of the study (day 18), sections of joint tissue were H&E stained and imaged at various magnifications.

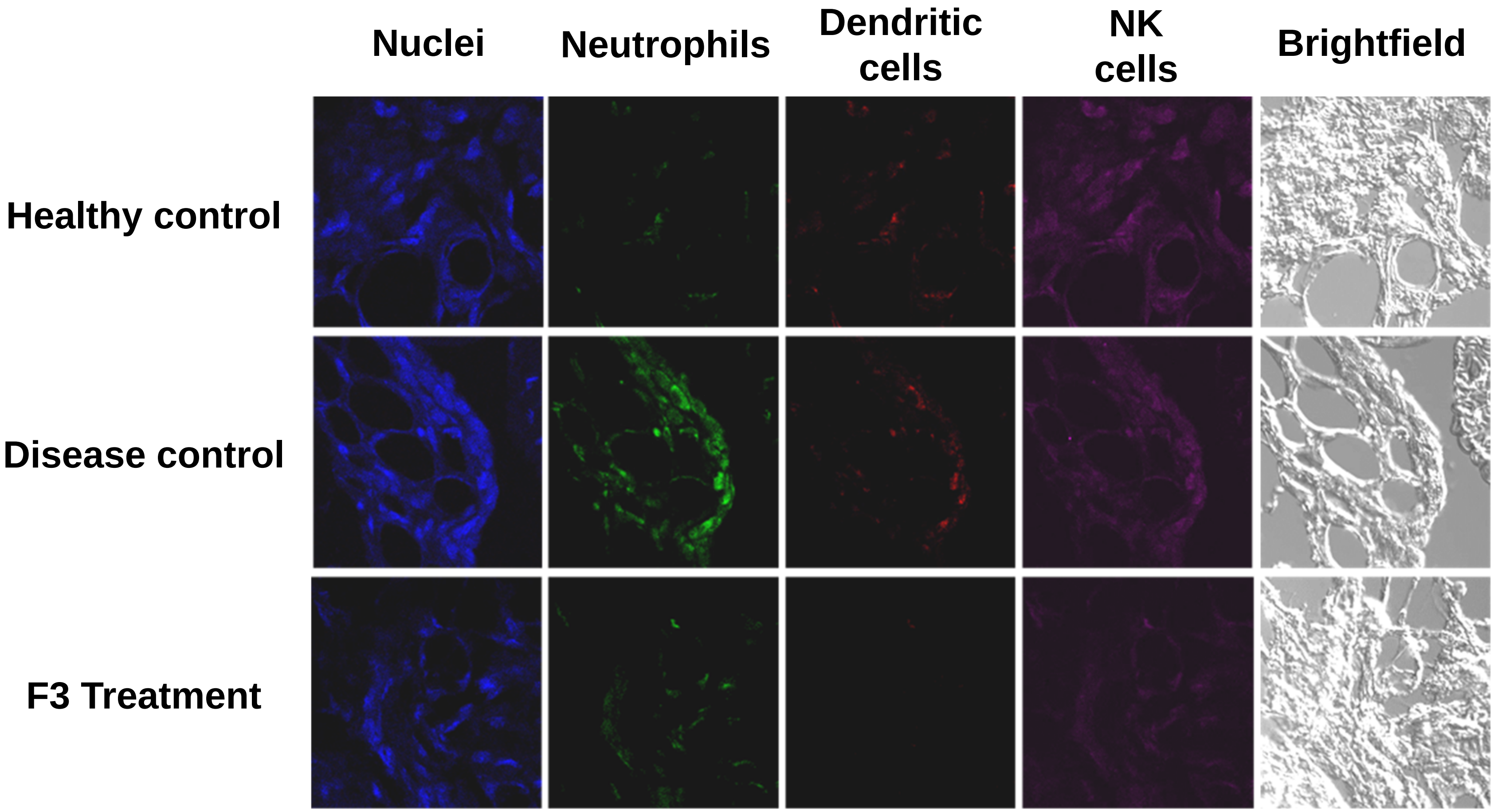

SI Figure 54. Immunofluorescent staining of different immune cell populations in thin sections of joints following F3 treatment in mice induced to develop arthritis (from **Fig. 6K**). Arthritis was induced as described in methods and F3 (20 mg/kg) was injected intraperitoneally (day 1) only after arthritis scores reached ~4. At the end of the study (day 18), sections of joint tissue were stained with cell specific markers and imaged via fluorescent microscopy.

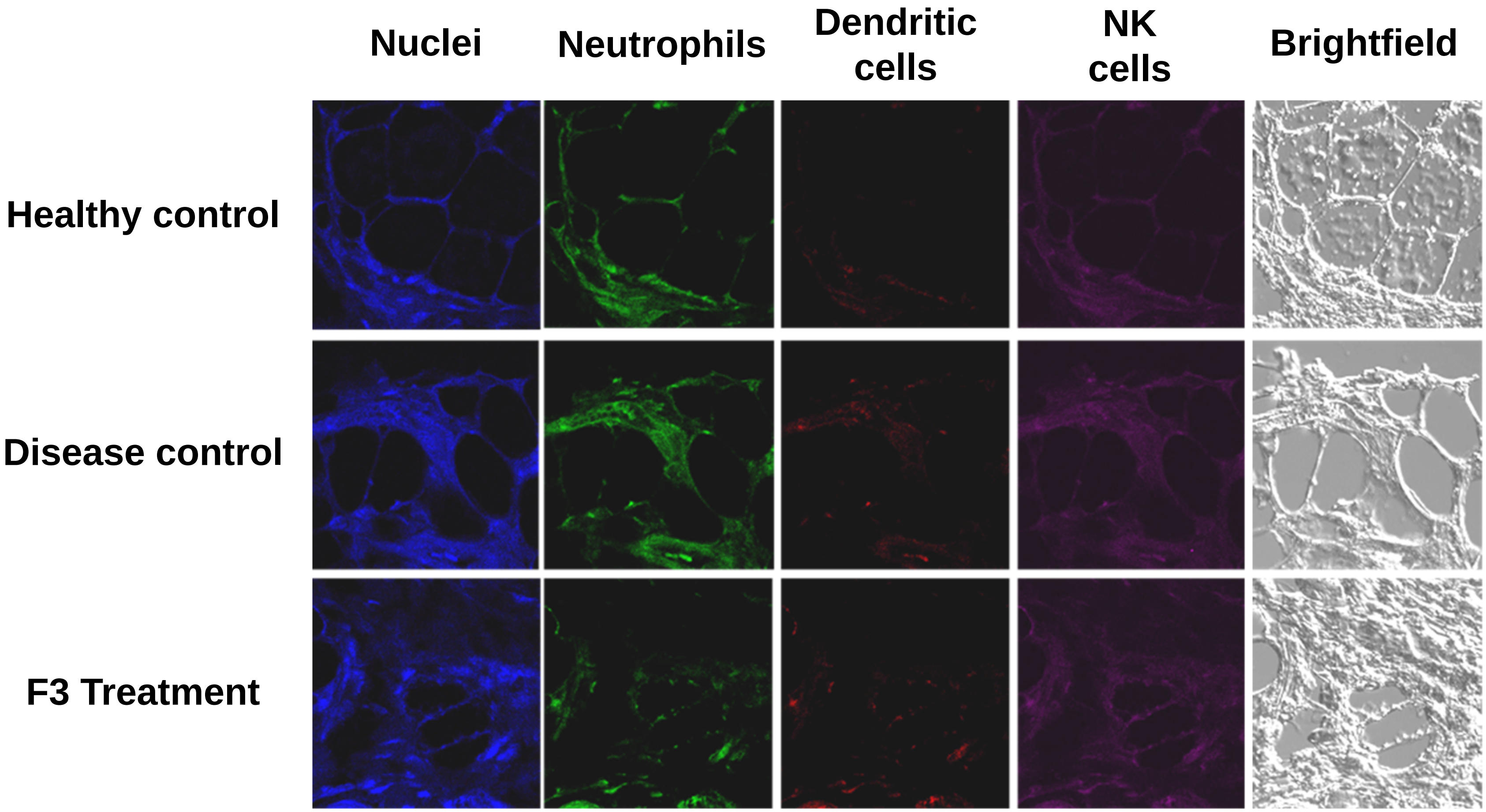

SI Figure 55. Immunofluorescent staining of different immune cell populations in thin sections of joints following F3 treatment in mice induced to develop arthritis (from **Fig. 6K**). Arthritis was induced as described in methods and F3 (20 mg/kg) was injected intraperitoneally (day 1) only after arthritis scores reached ~4. At the end of the study (day 18), sections of joint tissue were stained with cell specific markers and imaged via fluorescent microscopy.

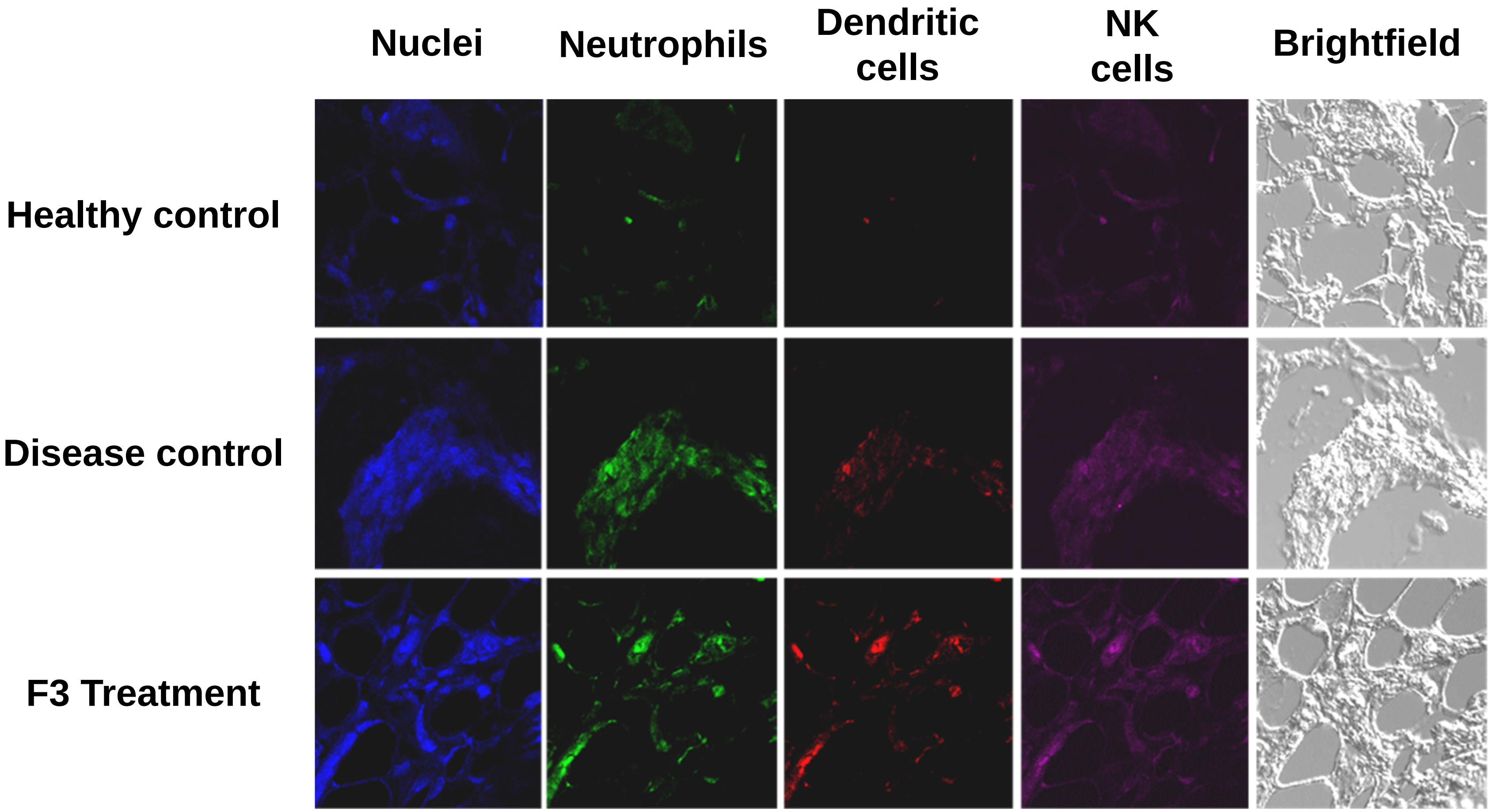

SI Figure 56. Immunofluorescent staining of different immune cell populations in thin sections of joints following F3 treatment in mice induced to develop arthritis (from **Fig. 6K**). Arthritis was induced as described in methods and F3 (20 mg/kg) was injected intraperitoneally (day 1) only after arthritis scores reached ~4. At the end of the study (day 18), sections of joint tissue were stained with cell specific markers and imaged via fluorescent microscopy.

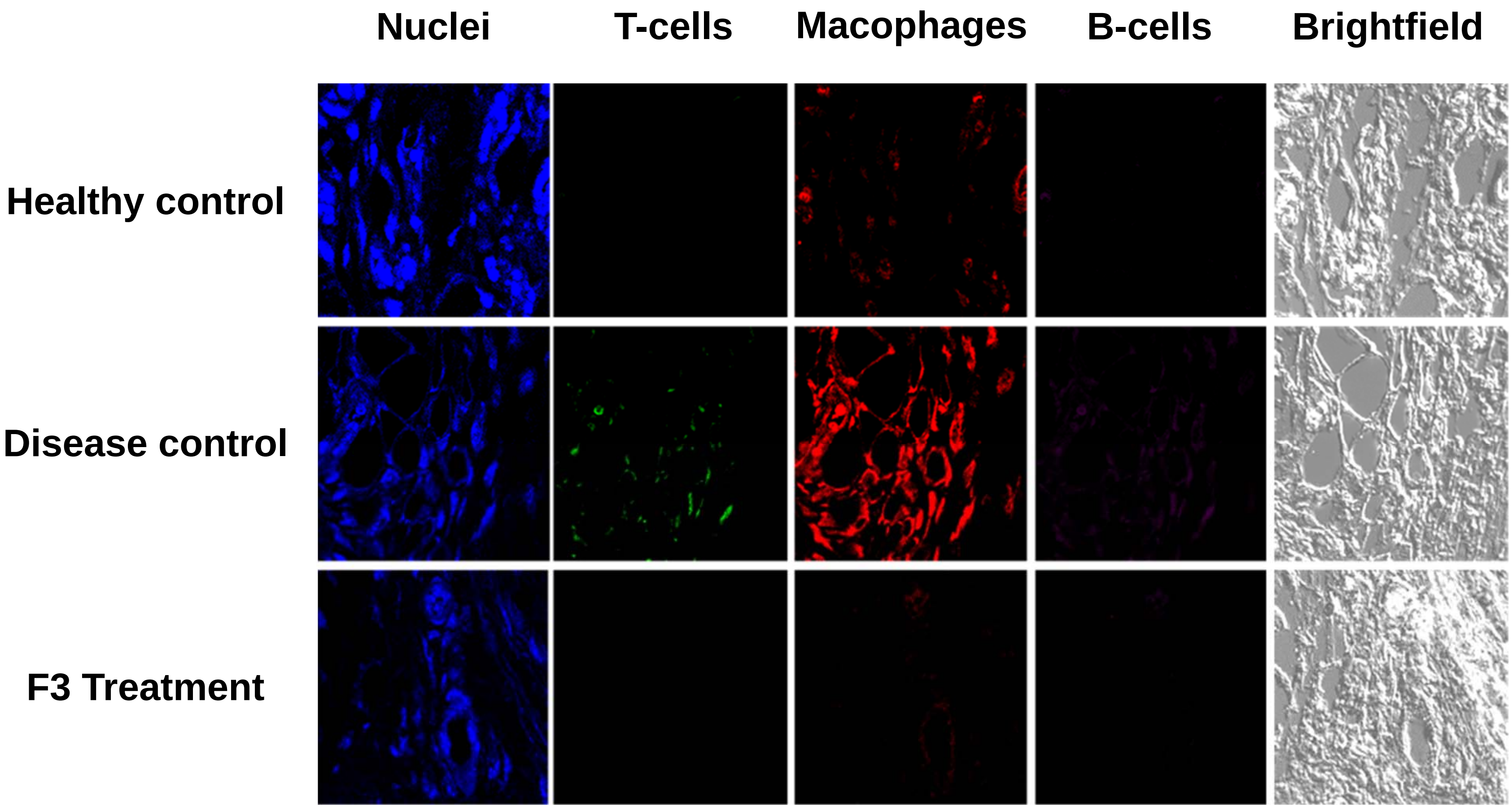

SI Figure 57. Immunofluorescent staining of different immune cell populations in thin sections of joints following F3 treatment in mice induced to develop arthritis (from **Fig. 6K**). Arthritis was induced as described in methods and F3 (20 mg/kg) was injected intraperitoneally (day 1) only after arthritis scores reached ~4. At the end of the study (day 18), sections of joint tissue were stained with cell specific markers and imaged via fluorescent microscopy.

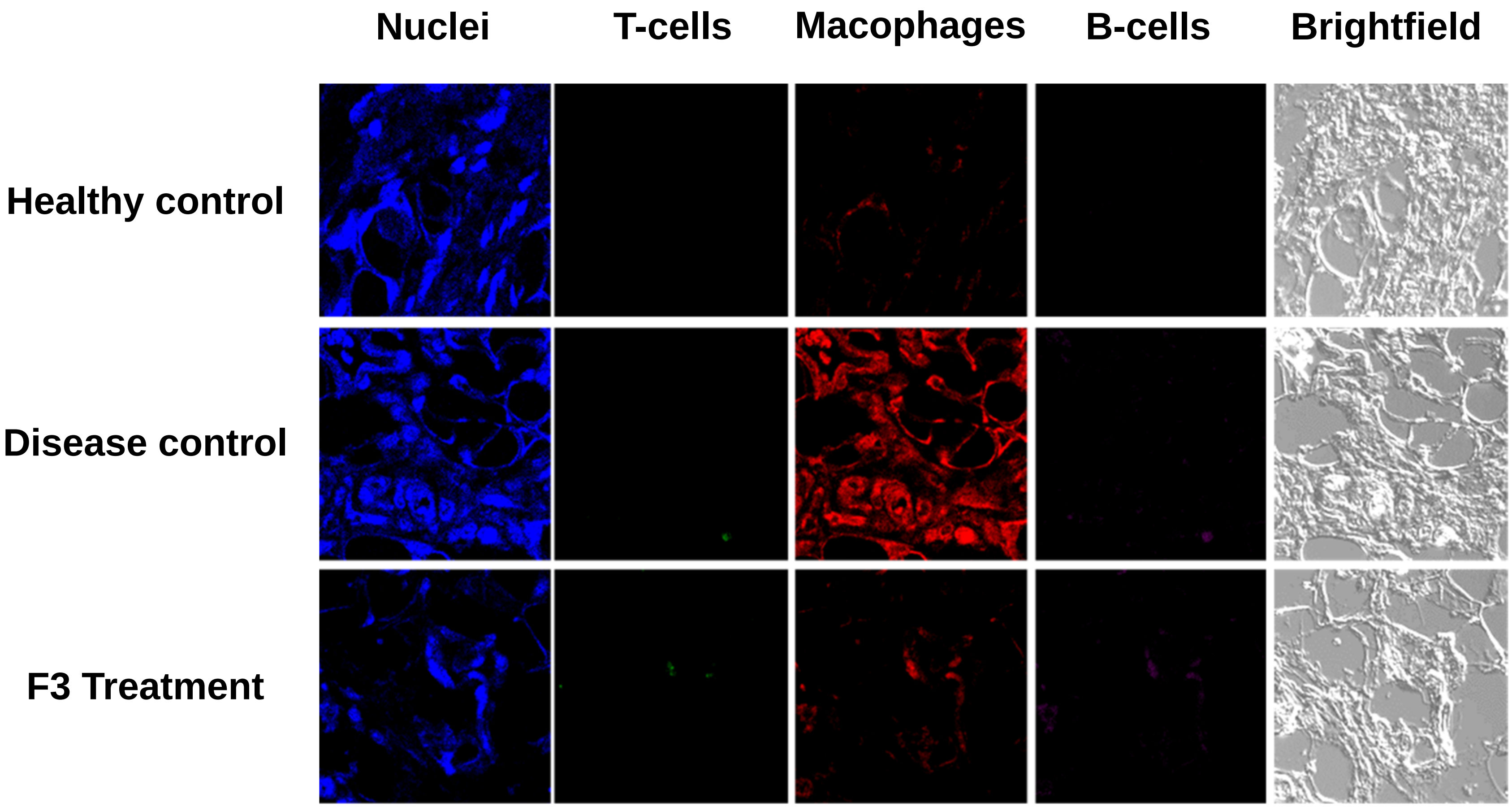

SI Figure 58. Immunofluorescent staining of different immune cell populations in thin sections of joints following F3 treatment in mice induced to develop arthritis (from **Fig. 6K**). Arthritis was induced as described in methods and F3 (20 mg/kg) was injected intraperitoneally (day 1) only after arthritis scores reached ~4. At the end of the study (day 18), sections of joint tissue were stained with cell specific markers and imaged via fluorescent microscopy.

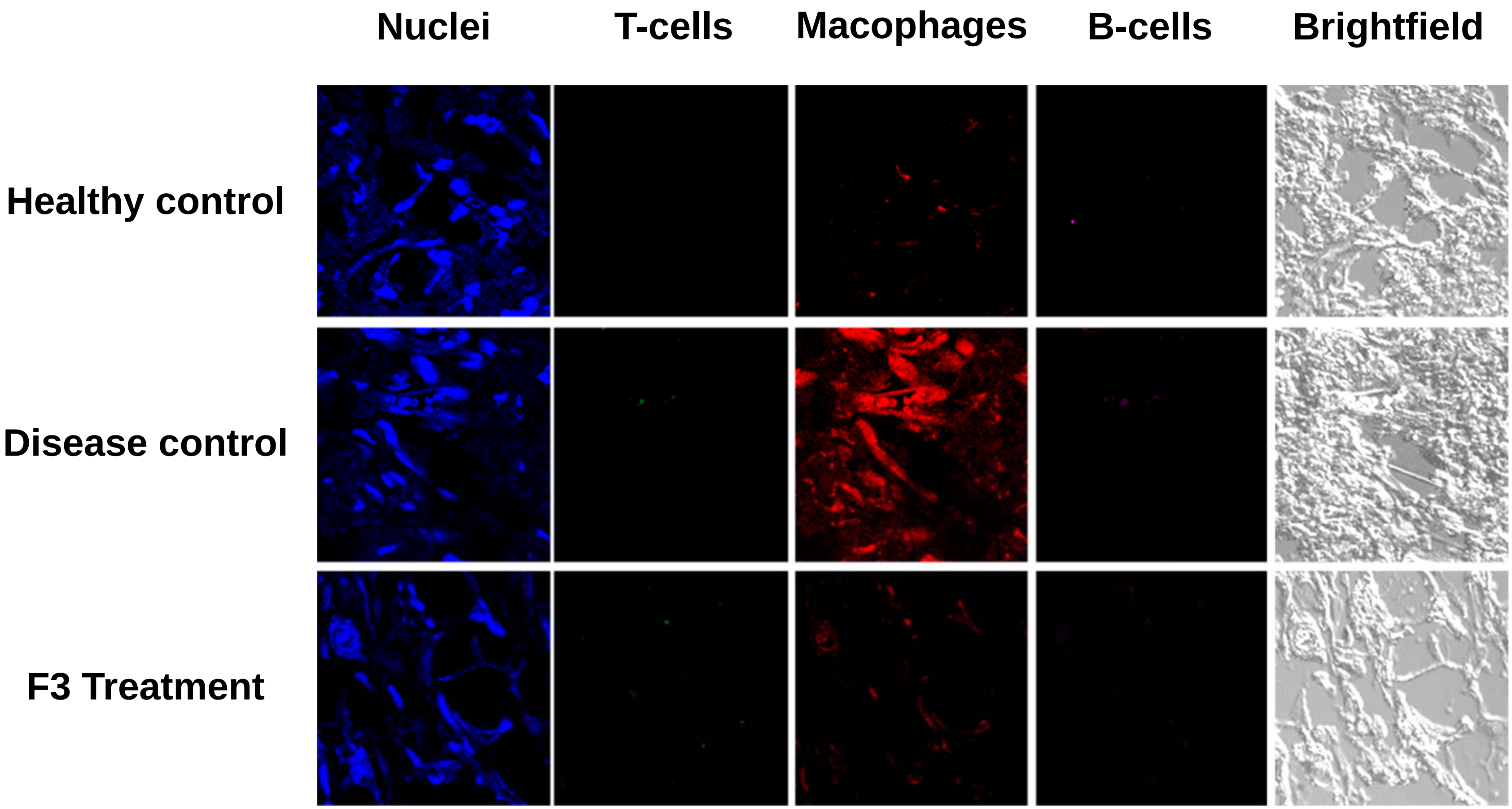

SI Figure 59. Immunofluorescent staining of different immune cell populations in thin sections of joints following F3 treatment in mice induced to develop arthritis (from **Fig. 6K**). Arthritis was induced as described in methods and F3 (20 mg/kg) was injected intraperitoneally (day 1) only after arthritis scores reached ~4. At the end of the study (day 18), sections of joint tissue were stained with cell specific markers and imaged via fluorescent microscopy.

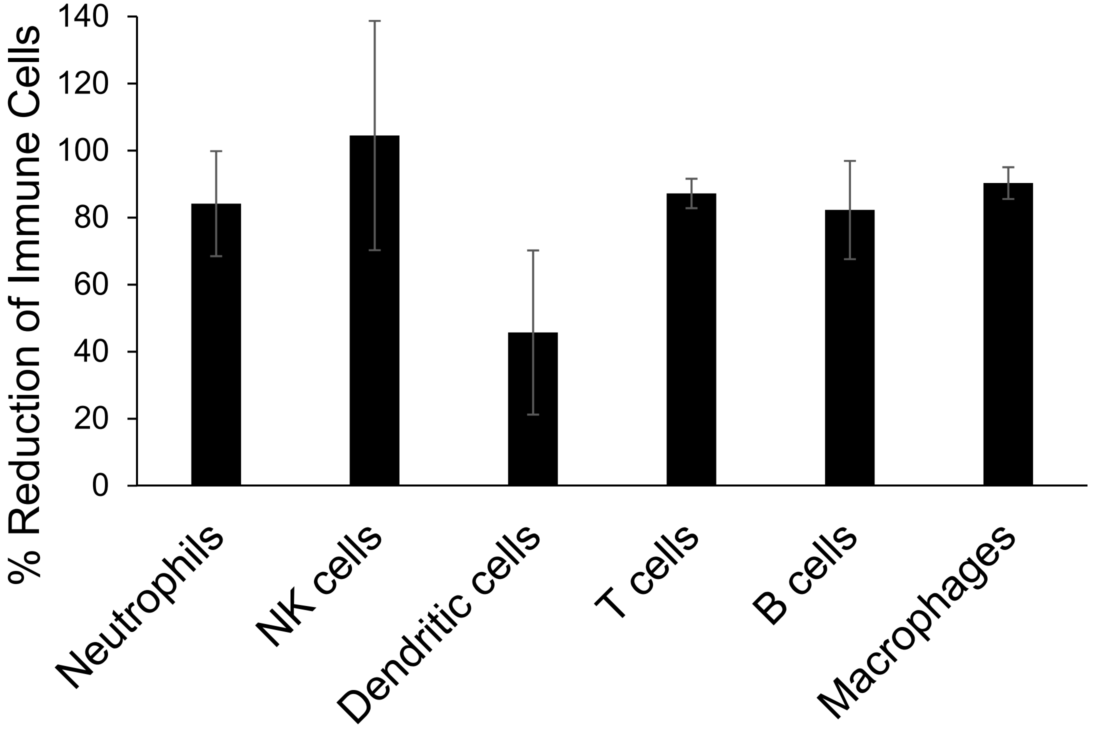

SI Figure 60. Percent reduction of different immune cell types in joint sections following F3 treatment in mice induced to develop arthritis (from **Fig. 6L**). Arthritis was induced as described in methods and F3 (20 mg/kg) was injected intraperitoneally (day 1) only after arthritis scores reached ~4. At the end of the study (day 18), sections of joint tissue were stained with cell specific markers and imaged via fluorescent microscopy. Staining intensity for each immune cell type was quantitated and the percent reduction in the number of each subpopulation of immune cells after treatment with F3 mAb was calculated where the average intensity in healthy and disease tissue was set at 100% and 0% reduction, respectively. Error bars represent standard error.

Macrophages

1-Way Anova

	SS	df	MS	F	p
Between	564.51	2	282.25	11.18	0.0095
Within	151.47	6	25.25		
Total	715.98	8			

Tukey Test (p-value)

	Healthy	Disease	F3
Healthy	-	0.0122	0.9000
Disease		-	0.01925
F3			-

Neutrophils

1-Way Anova

	SS	df	MS	F	p
Between	21.67	2	10.84	8.68	0.0170
Within	7.49	6	1.25		
Total	29.16	8			

Tukey Test (p-value)

	Healthy	Disease	F3
Healthy	-	0.0192	0.8050
Disease		-	0.0393
F3			-

NK cells

1-Way Anova

	SS	df	MS	F	p
Between	0.30	2	0.15	3.97	0.0796
Within	0.23	6	0.04		
Total	0.52	8			

T cells

1-Way Anova

	SS	df	MS	F	p
Between	13,142	2	6571	33.15	0.0006
Within	1,190	6	198		
Total	14,332	8			

Tukey Test (p-value)

	Healthy	Disease	F3
Healthy	-	0.0010	0.6241
Disease		-	0.0015
F3			-

B cells

1-Way Anova

	SS	df	MS	F	p
Between	41.81	2	20.91	9.77	0.0130
Within	12.84	6	2.14		
Total	54.65	8			

Tukey Test (p-value)

	Healthy	Disease	F3
Healthy	-	0.0142	0.7413
Disease		-	0.03296
F3			-

Dendritic cells

1-Way Anova

	SS	df	MS	F	p
Between	8.56	2	4.28	5.92	0.0381
Within	4.34	6	0.72		
Total	12.91	8			

Tukey Test (p-value)

	Healthy	Disease	F3
Healthy	-	0.0319	0.2282
Disease		-	0.32698
F3			-

SI Figure 61. Statistical analysis of differences in immune cell types in joint sections following F3 treatment in mice induced to develop arthritis (from **Fig. 6L**). Arthritis was induced as described in methods and F3 (20 mg/kg) was injected intraperitoneally (day 1) only after arthritis scores reached ~4. At the end of the study (day 18), sections of joint tissue were stained with cell specific markers and imaged via fluorescent microscopy. Staining intensity for each immune cell type was quantitated and a 1-way ANOVA followed by a post-hoc Tukey test was used to determine if there were any significant differences between groups. A p-value < 0.05 was considered significant and highlighted in yellow.

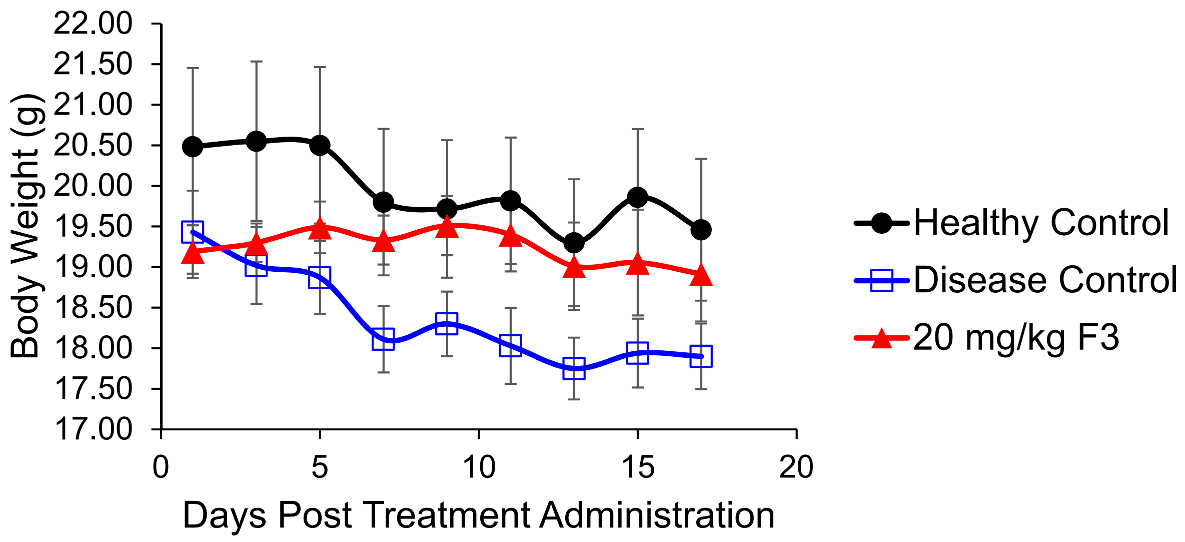

SI Figure 62. Changes in body weight following F3 treatment in mice induced to develop arthritis (from **Fig. 6**). Arthritis was induced as described in methods and F3 (20 mg/kg) was injected intraperitoneally (day 1) only after arthritis scores reached ~4. Body weight was recorded throughout the study. Error bars represent standard error.

Healthy (Vehicle Control) - Body Weight (g)

	Days								
	1	3	5	7	9	11	13	15	17
n	6	6	6	6	6	6	6	6	6
average	20.48	20.55	20.50	19.80	19.72	19.82	19.30	19.86	19.46
standard deviation	2.38	2.41	2.36	2.21	2.07	1.91	1.92	2.06	2.14
standard error	0.97	0.98	0.96	0.90	0.85	0.78	0.78	0.84	0.87

RA (Vehicle Control) - Body Weight (g)

	Days								
	1	3	5	7	9	11	13	15	17
n	10	10	10	10	10	10	10	10	10
average	19.43	19.02	18.87	18.11	18.30	18.03	17.75	17.94	17.90
standard deviation	1.62	1.50	1.42	1.29	1.26	1.48	1.20	1.34	1.28
standard error	0.51	0.47	0.45	0.41	0.40	0.47	0.38	0.42	0.40

20 mg/kg F3 - Body Weight (g)

	Days								
	1	3	5	7	9	11	13	15	17
n	10	10	10	10	10	10	10	10	10
average	19.19	19.30	19.49	19.33	19.51	19.40	19.01	19.06	18.91
standard deviation	1.03	0.75	1.01	0.96	1.16	1.43	1.70	2.06	1.85
standard error	0.33	0.24	0.32	0.30	0.37	0.45	0.54	0.65	0.58

SI Figure 63. Comparison of body weight following F3 treatment in mice induced to develop arthritis (from **Fig. 6**). Arthritis was induced as described in methods and F3 (20 mg/kg) was injected intraperitoneally (day 1) only after arthritis scores reached ~4. Table provides body weight measurements as a function of time.

1-way ANOVA - Body Weight

	Day 1			Day 3			Day 5			Day 7			Day 9			Day 11			Day 13			Day 15			Day 17		
SS (Between | Within | Total)	7.6	61.4	68.9	10.5	58.4	68.8	10.4	60.2	70.6	11.3	53.5	64.8	8.6	52.6	61.2	13.0	62.0	75.0	9.1	59.6	68.8	12.5	75.0	87.5	8.2	61.5	69.6
df (Between | Within | Total)	2	23	25	2	23	25	2	23	25	2	23	25	2	23	25	2	23	25	2	23	25	2	23	25	2	23	25
MS (Between | Within)	3.788	2.668		5.233	2.538		5.176	2.619		5.663	2.326		4.303	2.287		6.503	2.696		4.568	2.710		6.241	3.409		4.092	3.073	
F	1.420			2.062			1.976			2.435			1.882			2.412			1.685			1.831			1.332		
p-value	0.2622			0.1501			0.1614			0.1099			0.1751			0.1119			0.2085			0.1839			0.2864		

post-hoc Tukey Test (p-value)

	1	3	5	7	9	11	13	15	17
Healthy vs. Disease	-	-	-	-	-	-	-	-	-
Healthy vs. F3 (20 mg/kg)	-	-	-	-	-	-	-	-	-
Disease vs. F3 (20 mg/kg)	-	-	-	-	-	-	-	-	-

SI Figure 64. Statistical analysis of differences in body weight following F3 treatment in mice induced to develop arthritis (from **Fig. 6**). Arthritis was induced as described in methods and F3 (20 mg/kg) was injected intraperitoneally (day 1) only after arthritis scores reached ~4. A 1-way ANOVA followed by a post-hoc Tukey test was used to determine if there were any significant differences in body weight between groups. A p-value < 0.05 was considered significant and highlighted in yellow.

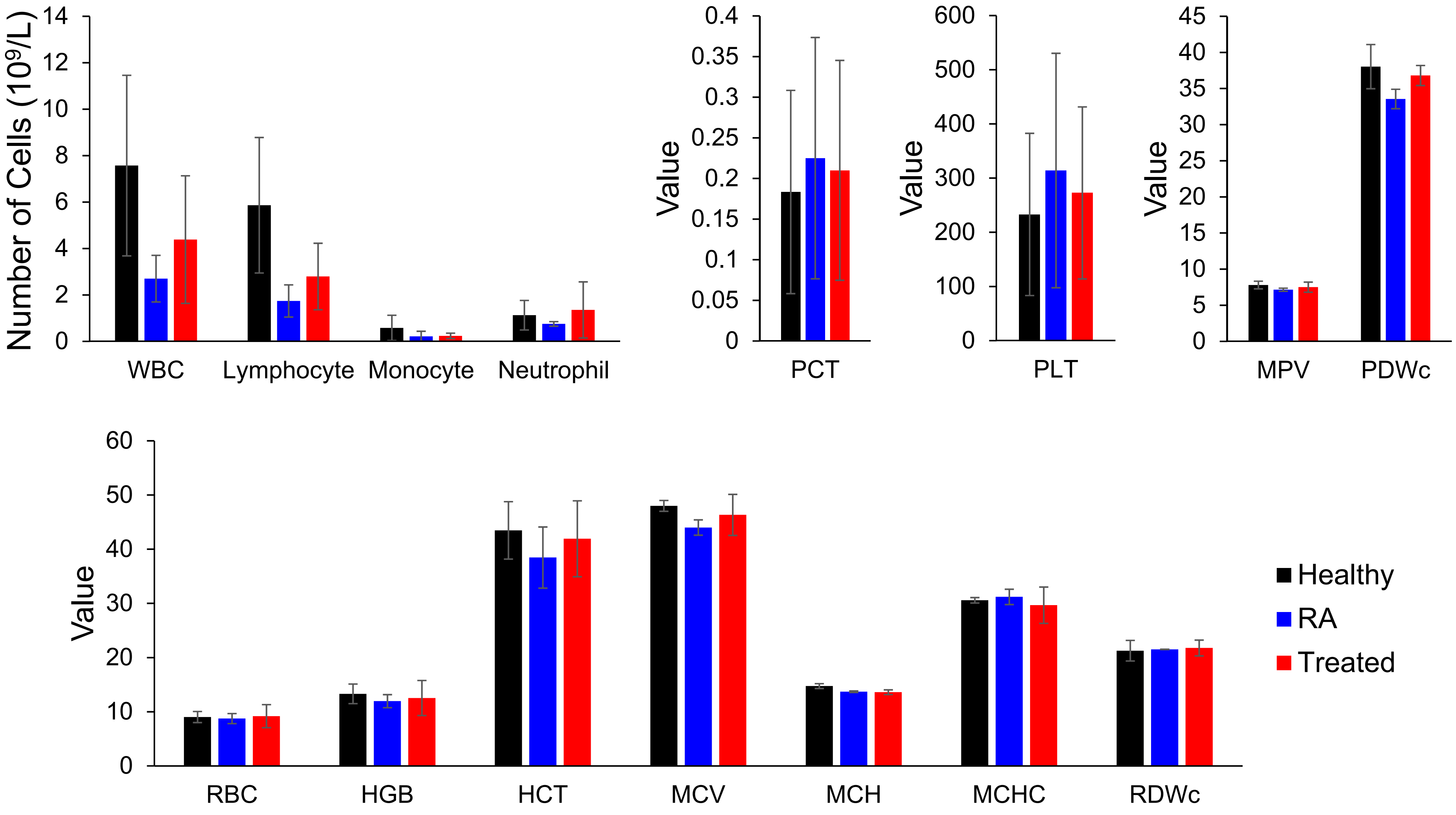

SI Figure 65. Changes in complete blood count values following F3 treatment in mice induced to develop arthritis (from **Fig. 6**). Arthritis was induced as described in methods and F3 (20 mg/kg) was injected intraperitoneally (day 1) only after arthritis scores reached ~4. On Day 18, mice were sacrificed and a complete blood count was performed including total white blood cells, lymphocytes, monocytes, neutrophils, red blood cells (RBC), hemoglobin (HGB), hematocrit (HCT), mean corpuscular volume (MCV), mean corpuscular hemoglobin (MCH), mean corpuscular hemoglobin concentration (MCHC), red cell distribution width (RDWc), platelets (PLT), platelet hematocrit (PCT), mean platelet volume (MPV), and platelet distribution width (PDWc). Error bars represent standard deviation.

White Blood Cell Number
1-Way Anova

	SS	df	MS	F	p
Between	31.34	2	15.67	1.69	0.2750
Within	46.36	6	9.27		
Total	77.69	8			

Lymphocyte Number
1-Way Anova

	SS	df	MS	F	p
Between	24.19	2	12.09	2.80	0.1530
Within	21.61	6	4.32		
Total	45.80	8			

Monocyte Number
1-Way Anova

	SS	df	MS	F	p
Between	0.23	2	0.12	0.87	0.4723
Within	0.67	6	0.13		
Total	0.90	8			

Neutrophile Number
1-Way Anova

	SS	df	MS	F	p
Between	0.44	2	0.22	0.29	0.7587
Within	3.74	6	0.75		
Total	4.18	8			

RBC
1-Way Anova

	SS	df	MS	F	p
Between	0.25	2	0.12	0.05	0.9505
Within	11.97	6	2.39		
Total	12.21	8			

HGB
1-Way Anova

	SS	df	MS	F	p
Between	2.28	2	1.14	0.20	0.8268
Within	28.89	6	5.78		
Total	31.18	8			

HCT
1-Way Anova

	SS	df	MS	F	p
Between	30.60	2	15.30	0.41	0.6835
Within	186.13	6	37.23		
Total	216.73	8			

MCV
1-Way Anova

	SS	df	MS	F	p
Between	19.21	2	6.90	1.47	0.3147
Within	32.67	6	6.53		
Total	51.88	8			

MCH
1-Way Anova

	SS	df	MS	F	p
Between	2.25	2	1.13	6.98	0.0357
Within	0.81	6	0.16		
Total	3.06	8			

Tukey

	Healthy	RA	Treated
Healthy	-	0.0803	0.0402
RA		-	0.9000
Treated			-

MCHC
1-Way Anova

	SS	df	MS	F	p
Between	2.98	2	1.49	0.30	0.7546
Within	24.95	6	4.99		
Total	27.93	8			

RDWc
1-Way Anova

	SS	df	MS	F	p
Between	0.38	2	0.19	0.08	0.9227
Within	11.47	6	2.30		
Total	11.85	8			

PLT
1-Way Anova

	SS	df	MS	F	p
Between	7,972	2	3986	0.14	0.8724
Within	142,068	6	28413		
Total	150,041	8			

PCT
1-Way Anova

	SS	df	MS	F	p
Between	0.0023	2	0.0011	0.06	0.9396
Within	0.0899	6	0.0180		
Total	0.0922	8			

MPV
1-Way Anova

	SS	df	MS	F	p
Between	0.51	2	0.26	0.80	0.4979
Within	1.59	6	0.32		
Total	2.10	8			

PDWc
1-Way Anova

	SS	df	MS	F	p
Between	24.71	2	12.35	2.53	0.1738
Within	24.37	6	4.87		
Total	49.08	8			

SI Figure 66. Statistical analysis of differences in complete blood count values following F3 treatment in mice induced to develop arthritis (from **Fig. 6**). Arthritis was induced as described in methods and F3 (20 mg/kg) was injected intraperitoneally (day 1) only after arthritis scores reached ~4. On Day 18, mice were sacrificed and a complete blood count was performed including total white blood cells, lymphocytes, monocytes, neutrophils, red blood cells (RBC), hemoglobin (HGB), hematocrit (HCT), mean corpuscular volume (MCV), mean corpuscular hemoglobin (MCH), mean corpuscular hemoglobin concentration (MCHC), red cell distribution width (RDWc), platelets (PLT), platelet hematocrit (PCT), mean platelet volume (MPV), and platelet distribution width (PDWc). A 1-way ANOVA followed by a post-hoc Tukey test was used to determine if there were any significant differences between groups. A p-value < 0.05 was considered significant and highlighted in yellow.
